# Design, Synthesis, and Evaluation of a New Series of Thiazole-Based Anticancer Agents as Potent Akt Inhibitors

**DOI:** 10.3390/molecules23061318

**Published:** 2018-05-31

**Authors:** Mehlika Dilek Altıntop, Belgin Sever, Gülşen Akalın Çiftçi, Ahmet Özdemir

**Affiliations:** 1 Department of Pharmaceutical Chemistry, Faculty of Pharmacy, Anadolu University, 26470 Eskişehir, Turkey; belginsever@anadolu.edu.tr (B.S.); ahmeto@anadolu.edu.tr (A.Ö.); 2 Department of Biochemistry, Faculty of Pharmacy, Anadolu University, 26470 Eskişehir, Turkey; gakalin@anadolu.edu.tr

**Keywords:** apoptosis, Akt, cancer, Lipinski’s rule of five, molecular docking, thiazole

## Abstract

In an attempt to develop potent anticancer agents targeting Akt, new thiazole derivatives (**1**–**10**) were synthesized and investigated for their cytotoxic effects on A549 human lung adenocarcinoma, C6 rat glioma, and NIH/3T3 (healthy) mouse embryonic fibroblast cell lines. The most potent compounds were also investigated for their effects on apoptosis and Akt pathway. The most promising anticancer agent was found to be 2-[2-((4-(4-cyanophenoxy)phenyl)methylene)hydrazinyl]-4-(4-cyanophenyl)thiazole (**6**), due to its selective inhibitory effects on A549 and C6 cells with IC_50_ values of 12.0 ± 1.73 µg/mL and 3.83 ± 0.76 µg/mL, respectively. Furthermore, compound **6** increased early and late apoptotic cell population (32.8%) in C6 cell line more than cisplatin (28.8%) and significantly inhibited the Akt enzyme. The molecular docking study was performed to predict the possible binding modes of compounds **A**, **6,** and **8** inside the active site of Akt (PDB code: 4EJN). Molecular docking simulations were found to be in accordance with in vitro studies and, hence, supported the biological activity. A computational study for the prediction of absorption, distribution, metabolism and excretion (ADME) properties of all compounds was also performed. On the basis of Lipinski’s rule of five, the compounds were expected to be potential orally bioavailable agents.

## 1. Introduction

Lung cancer is the leading cause of cancer-related deaths worldwide in both men and women [1]. As the most common lung cancer type, non-small cell lung cancer (NSCLC) poses a continuous and serious threat to public health. Despite significant advances in both diagnostic and therapeutic approaches, the overall survival for NSCLC patients still remains poor [2]. One main impediment for the treatment of NSCLC is that most patients are diagnosed at a late stage when the prognosis is poor and therapeutic options are limited [2,3,4].

Gliomas are the most common primary malignant brain tumors and ranked among the most aggressive human cancers [5,6]. Despite advances in standard therapy, the prognosis for patients with gliomas remains poor [6]. Current standard therapy for glioma patients is surgery followed by radiotherapy and adjuvant chemotherapy [5,6]. Efficacy of chemotherapy is limited due to poor drug delivery and inherent chemo- and radio-resistance [6].

Phosphatidylinositol-3-kinase (PI3K)/Akt pathway is one of the most frequently deregulated signaling pathways in human cancers. Akt, a key component of this pathway, is overexpressed or activated in a variety of human cancers, including gliomas, lung, breast, ovarian, gastric, and pancreatic carcinomas. Inhibition of Akt signalling results in apoptosis and growth inhibition of tumor cells with elevated Akt activity. As a result, Akt has been validated as a viable and feasible target for anticancer agents and several promising Akt inhibitors are in various stages of clinical evaluation [7,8,9,10].

Thiazole has attracted a great deal of interest as a versatile scaffold due to its pivotal role in the lead identification and optimization [11]. Diversely substituted thiazole derivatives embedded with a variety of functional groups are found in a large number of well-known naturally occurring compounds such as thiamine, and commercial synthetic drugs [11,12,13,14]. The myriad spectrum of therapeutic applications associated with thiazoles has encouraged medicinal chemists to design and synthesize a large number of new thiazole-based therapeutic agents. Several thiazole derivatives have been reported to show significant antimicrobial, antitumor, antiviral, antimalarial, anthelmintic, antidiabetic, antioxidant, anticonvulsant, antiasthmatic, analgesic, anti-inflammatory, and other activities [11,12,13,14,15,16,17,18,19,20]. In particular, the clinical efficacy of tiazofurin, bleomycins (BLMs), and dasatinib has pointed out the medicinal significance of thiazole scaffold for the treatment of cancer [14,15,16]. Recent studies have indicated that thiazole derivatives show significant antitumor activity against different cancer cell lines through the inhibition of histone deacetylases (HDACs), pro-matrix metalloproteinase activation, signal transducer and activator of transcription 3 (STAT3), Bcl-2 family, and kinases including Akt isoforms, aurora kinases, and cyclin-dependent kinases (CDKs) [17,18,19,20]. Besides, hydrazides–hydrazones have been identified as an important class of lead compounds for the development of new chemical entities to treat various diseases due to their unique structural features and diverse biological activities [21,22,23]. In particular, many studies have pointed out the pivotal role of the hydrazone moiety for anticancer drug development. Hydrazone derivatives have been reported to show potent antitumor activity against various cancer cell lines such as A549 human lung adenocarcinoma, MCF-7 human breast adenocarcinoma, U-373 MG human glioblastoma, SK-OV-3 human ovary carcinoma, SK-MEL-2 human melanoma, HCT15 human colon carcinoma, MIA PaCa-2 human pancreas carcinoma, and HepG2 human hepatocellular carcinoma cell lines [22,23,24,25,26,27,28].

Prompted by the aforementioned findings [29,30,31] and in the continuation of our ongoing research on thiazolyl hydrazones [32,33,34] related to their anticancer activity, herein we described the design and synthesis of a new series of thiazolyl hydrazone derivatives as potential anticancer agents targeting Akt.

## 2. Results and Discussion

**Scheme 1:**
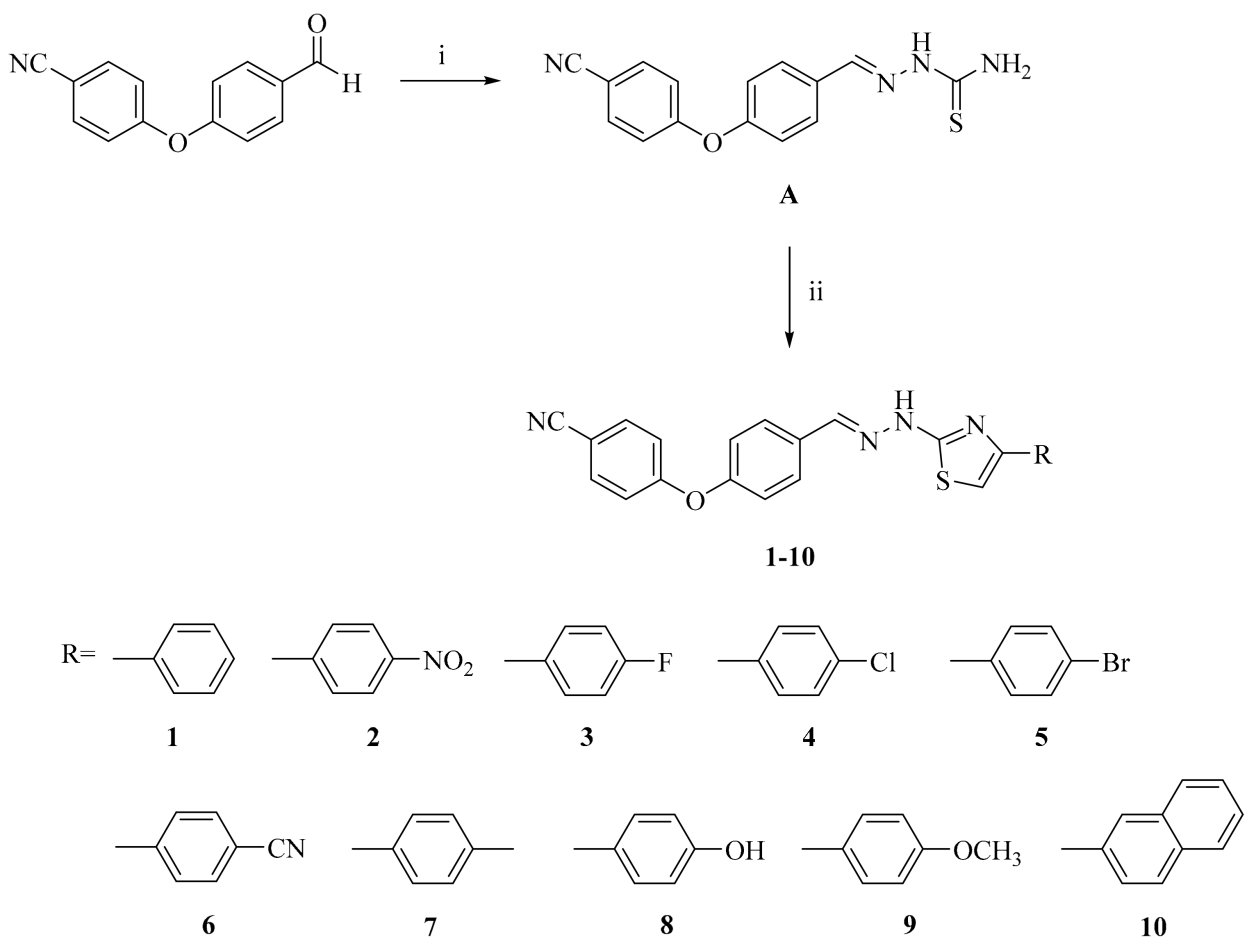
The synthetic route for the preparation of the thiazole derivatives (**1**–**10**). Reagents and conditions: (i) NH_2_CSNHNH_2_, ethanol, reflux, 12 h; (ii) RCOCH_2_Br, ethanol, reflux, 6 h.

The synthesis of the hitherto unreported compounds (**1**–**10**) was performed as outlined in Scheme 1. 4-(4-Cyanophenoxy)benzaldehyde thiosemicarbazone (**A**) was obtained via the treatment of 4-(4-cyanophenoxy)benzaldehyde with thiosemicarbazide. The ring closure reaction of compound **A** with substituted arylacyl bromides afforded final compounds (**1**–**10**). The structures of the compounds were confirmed by IR, ^1^H-NMR, ^13^C-NMR, and mass spectral data (see Supplementary Materials for further details).

MTT assay was performed to determine the antiproliferative effects of the compounds on A549 human lung adenocarcinoma and C6 rat glioma cell lines (Table 1). In order to evaluate whether the compounds were toxic or non-toxic to healthy cells, the cytotoxic effects of compounds **1**–**10** on NIH/3T3 mouse embryonic fibroblast cell line were also investigated using MTT assay.

Compound **6** was found to be the most promising anticancer agent in this series due to its significant inhibitory effects on C6 and A549 cell lines with IC_50_ values of 3.83 ± 0.76 µg/mL and 12 ± 1.73 µg/mL, respectively. This compound was also more effective than cisplatin on both cell lines. This outcome indicated that the cyano substituent on the phenyl ring at the 4th position of the thiazole scaffold significantly enhanced anticancer activity against both cell lines. IC_50_ value of compound **6** for NIH/3T3 cell line was found to be higher than 500 µg/mL indicating that anticancer activity of compound **6** was selective.

Compounds **A**, **2**, and **8** exhibited cytotoxic activity against A549 cell line with IC_50_ values of 92.5 ± 3.54, 100 ± 20 and 106.67 ± 5.77 µg/mL, respectively. Among these compounds, the anticancer activity of compound **2** was not selective (IC_50_ value for NIH/3T3 cell line = 71.67 ± 24.66 µg/mL). On the other hand, compounds **A**, **7**, and **8** showed notable anticancer activity against C6 cell line with IC_50_ values of 26.33 ± 1.53, 16 ± 5.66 and 5.83 ± 0.76 µg/mL, respectively. In particular, the hydroxy substituent on the phenyl ring at the 4th position of the thiazole scaffold significantly increased anticancer activity against C6 cell line.

**Figure 1:**
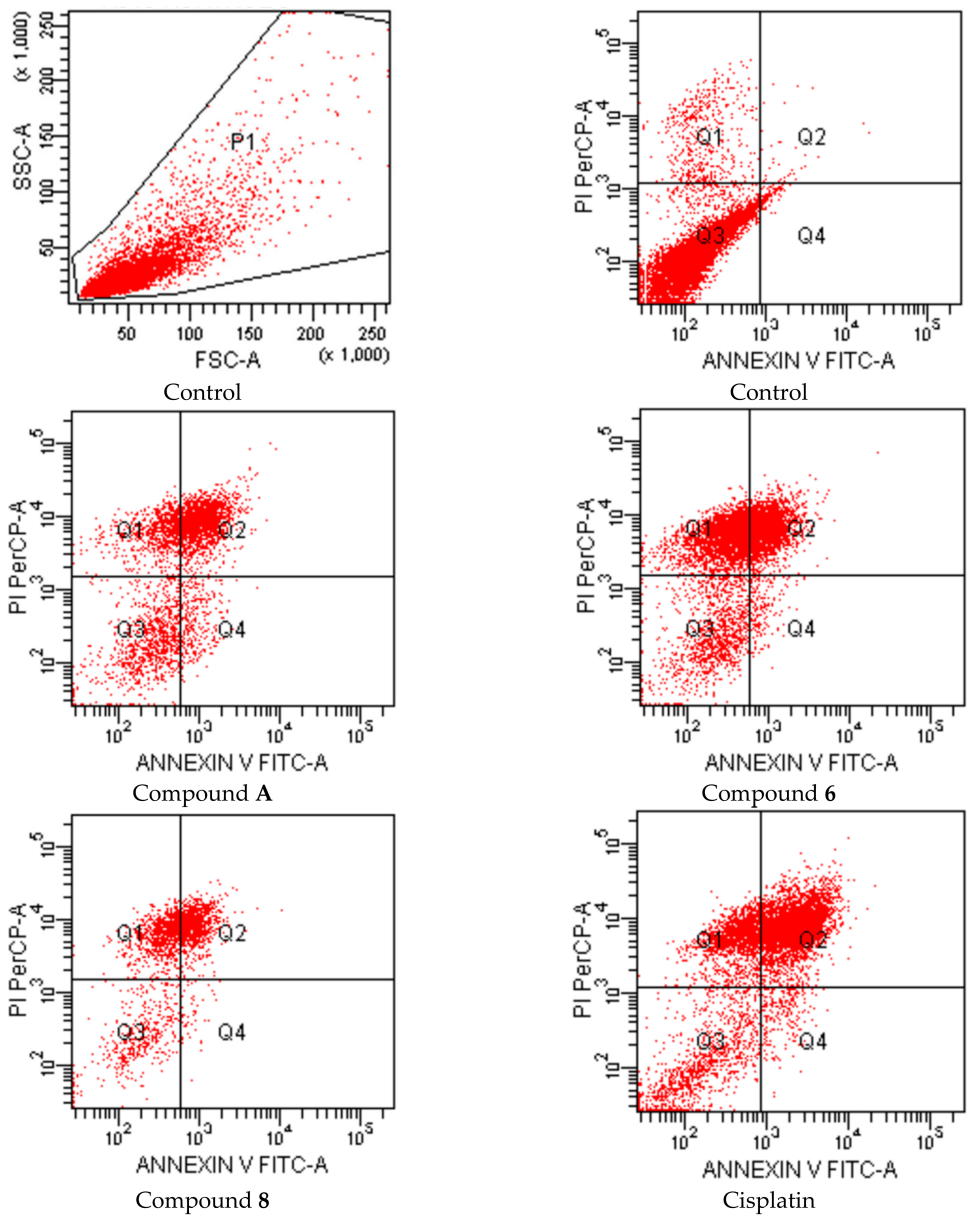
Flow cytometric analysis of A549 cells treated with IC_50_ values of compounds **A**, **6**, **8**, and cisplatin for 24 h. At least 10,000 cells were analyzed per sample, and quadrant analysis was performed.

The apoptotic effects of the most active anticancer agents in this series were analyzed for A549 and C6 cell lines based on Annexin V-PI binding capacities in flow cytometry. The early apoptotic effects of compounds **A**, **6**, **8**, and cisplatin on A549 cell line were determined as 7.6, 2.3, 2.1, and 2.6% respectively, whereas their late apoptotic effects were determined as 45.9, 36.5, 38.7 and 63.5%, respectively (Table 2 and Figure 1). According to these findings, compound **A** showed more apoptotic activity against A549 cell line than compounds **6** and **8**. This outcome pointed out the importance of the thiosemicarbazone moiety for apoptotic activity against A549 cell line.

**Figure 2:**
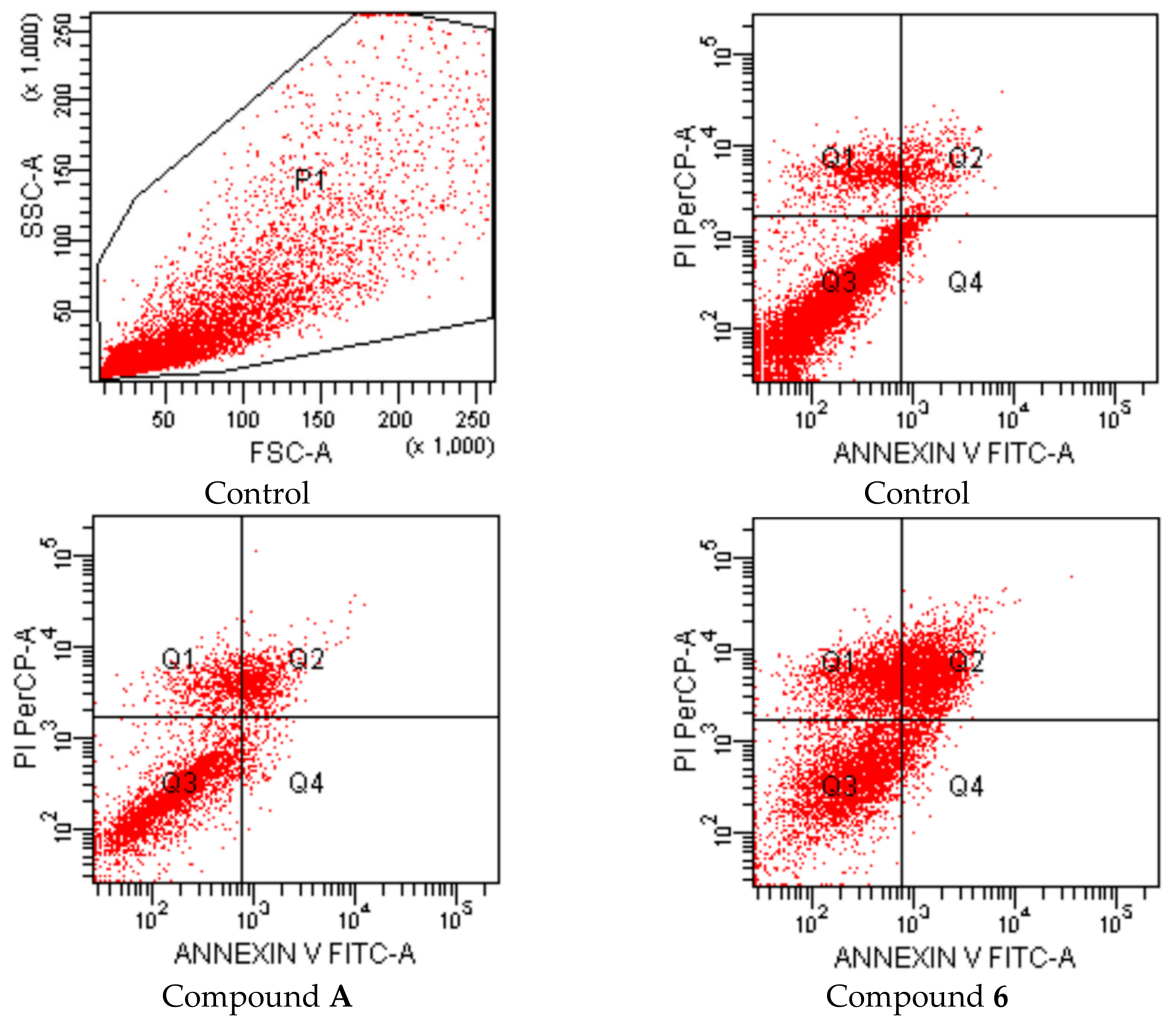

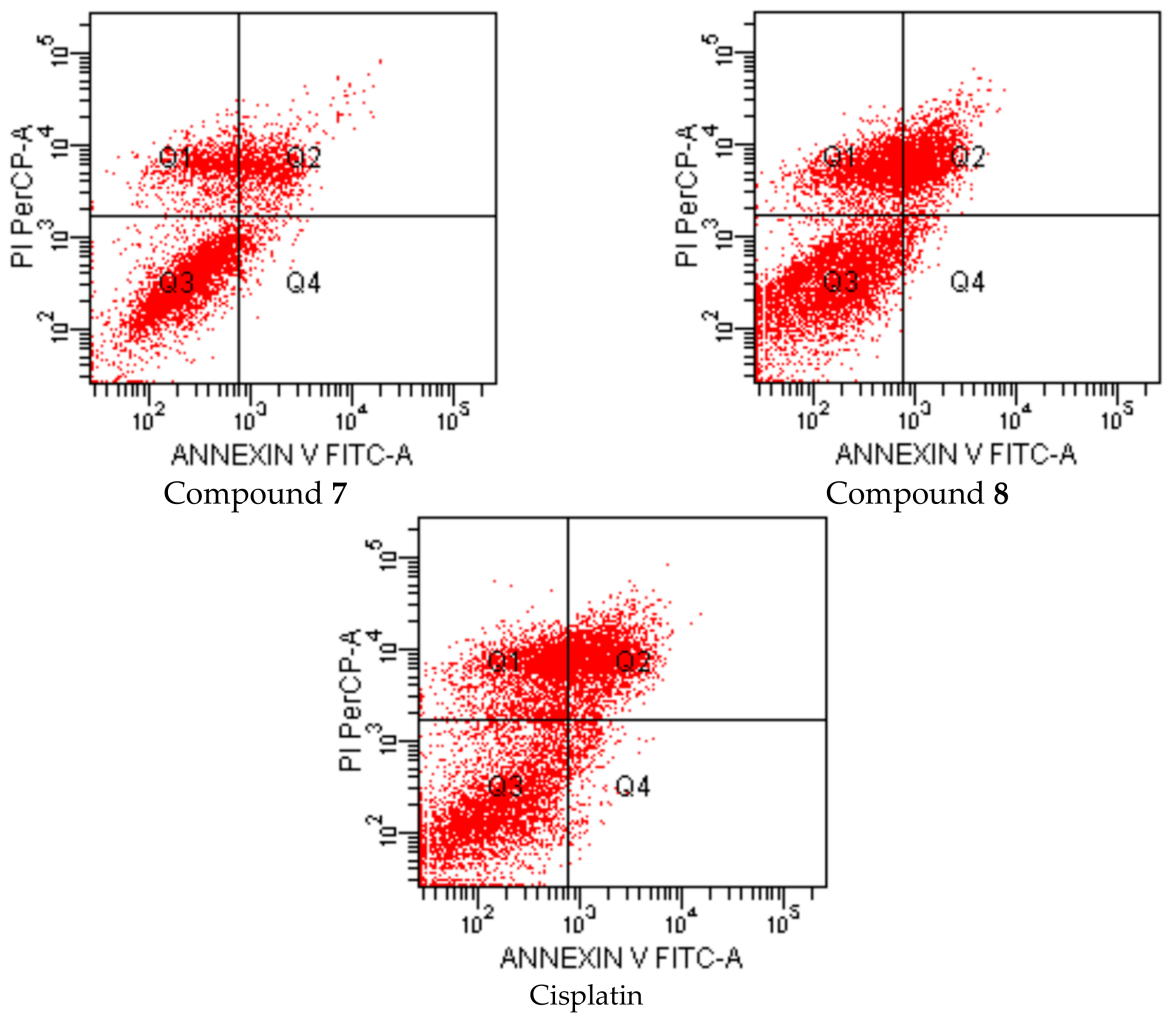
Flow cytometric analysis of C6 cells treated with IC_50_ values of compounds **A**, **6**, **7**, **8** and cisplatin for 24 h. At least 10,000 cells were analyzed per sample, and quadrant analysis was performed.

The early apoptotic effects of compounds **A**, **6**, **7**, **8**, and cisplatin on C6 cell line were determined as 3.2, 4.6, 3.2, 1.9, and 3.3% respectively, whilst their late apoptotic effects were determined as 13.1, 28.2, 14.3, 23.8, and 25.5%, respectively (Table 3 and Figure 2). According to flow cytometric analyses, compound **6** showed more apoptotic activity against C6 cell line than cisplatin. The late apoptotic effects of compound **8** were better than compounds **A** and **7**. The results indicated that the cyano substituent on the phenyl ring at the 4th position of the thiazole scaffold significantly enhanced apoptotic activity against C6 cell line.

As a consequence of the pivotal role of Akt in regulating diverse cellular functions including cell growth, proliferation, and survival [7,8,9,10], the most potent anticancer agents were investigated for their inhibitory effects on Akt activity (Table 4). The most potent Akt inhibitor in A549 cell line was found to be compound **A** (68.08 ± 2.48%) followed by compounds **8** (57.37 ± 17.30%) and **6** (45.77 ± 10.58%), whereas the most potent Akt inhibitor in C6 cell line was found to be compound **6** (71.66 ± 4.09%) followed by compound **7** (70.42 ± 10.37%).

**Figure 3:**
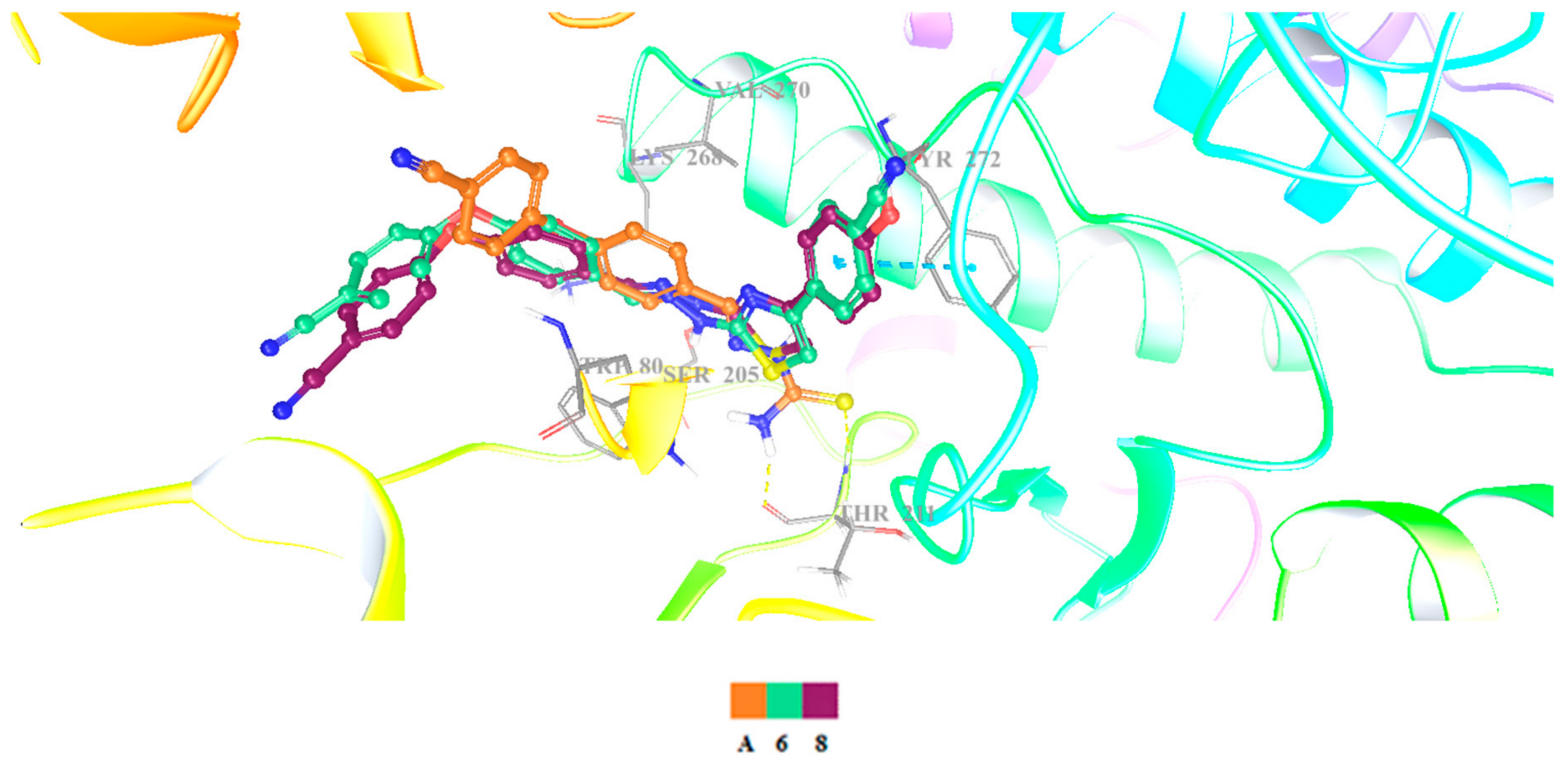
Docked poses of compounds **A**, **6** and **8** in the active site of Akt (Yellow dashes: Hydrogen bonding; Blue dashes: π–π interactions).

**Figure 4:**
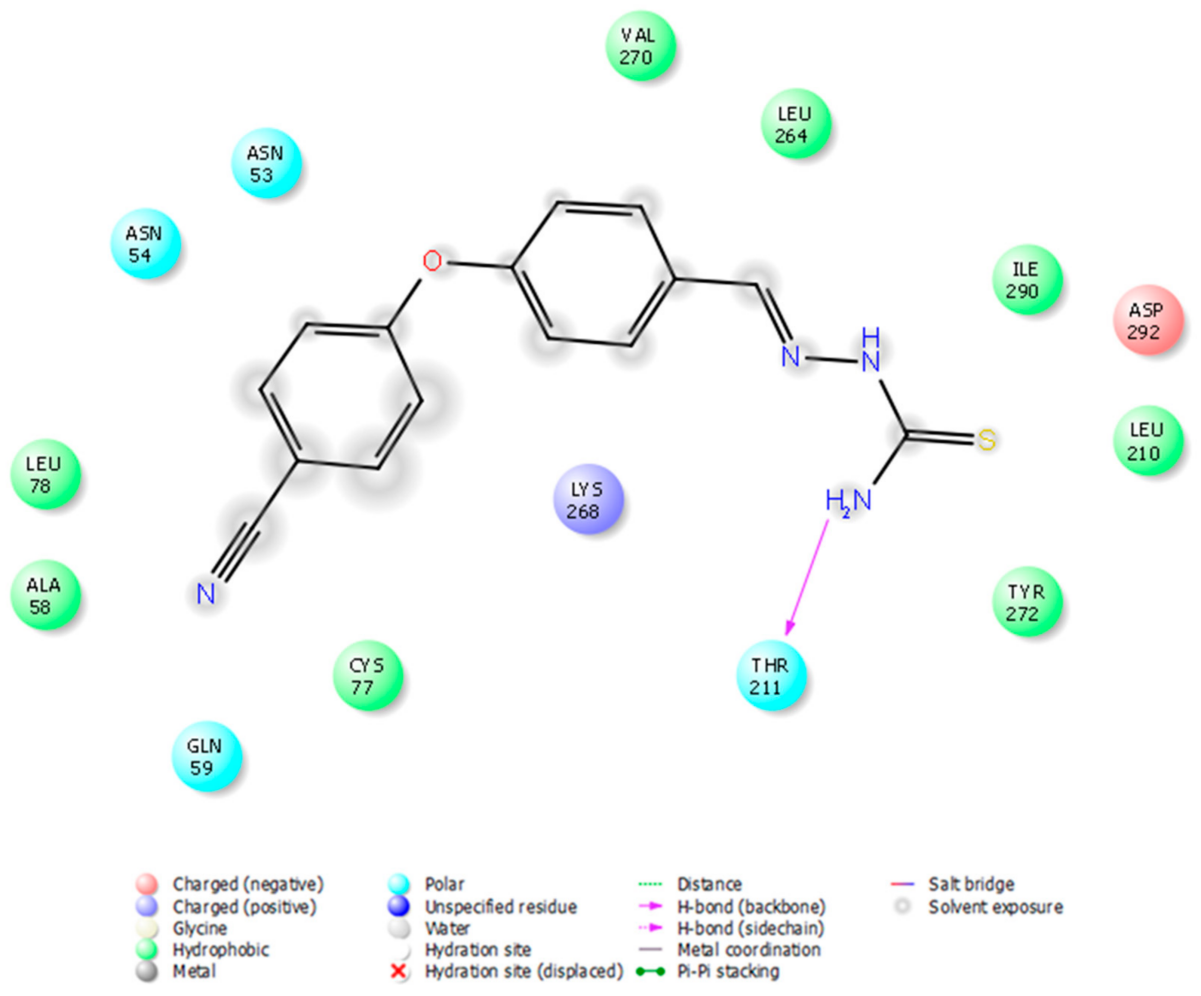
Docking interactions of compound **A** with proper residues in the active site of Akt.

**Figure 5:**
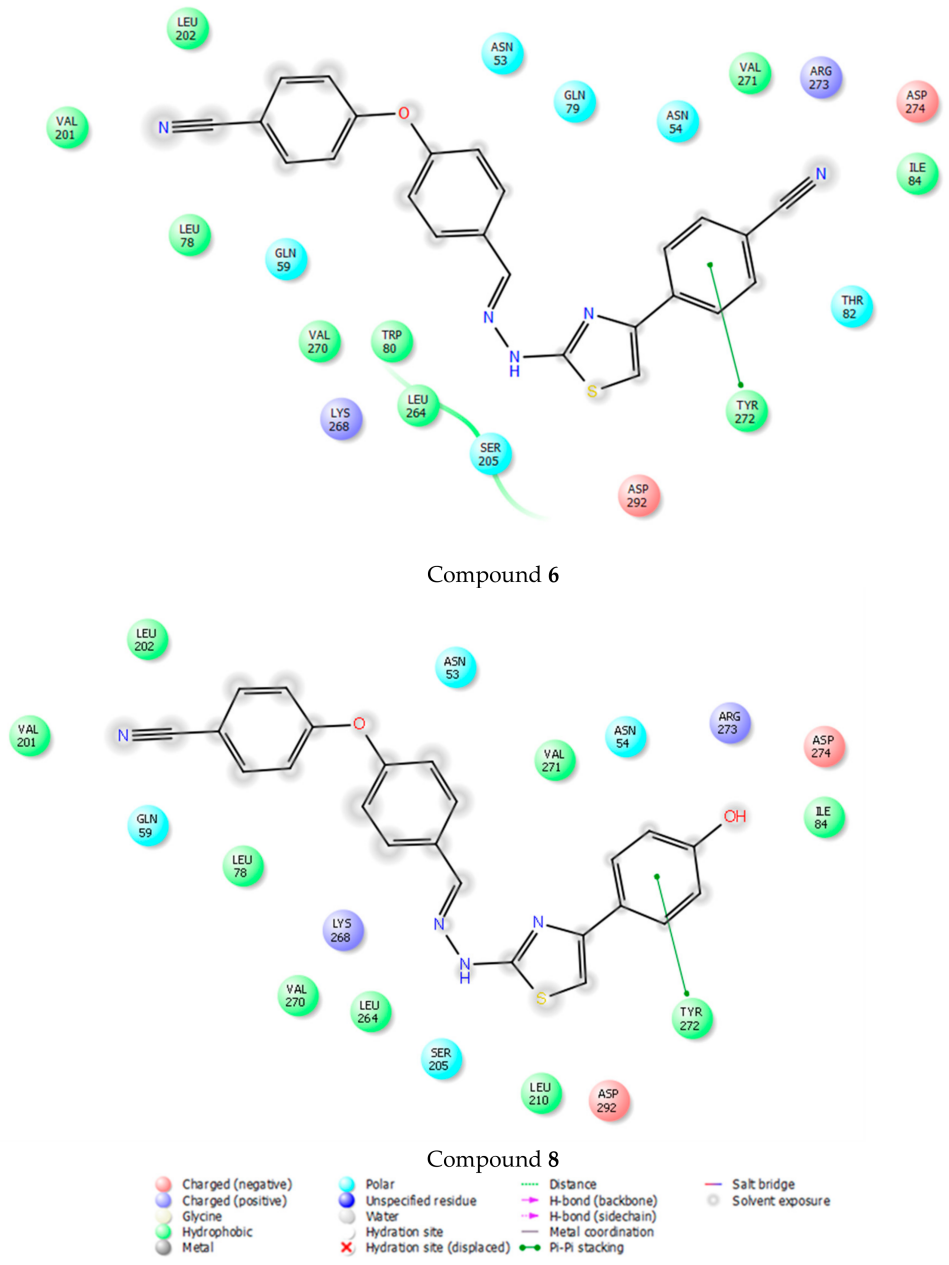
Docking interactions of compounds **6** and **8** with proper residues in the active site of Akt.

Based on their Akt inhibitory potencies in A549 cell line, molecular docking was carried out for evaluating and analyzing interactions of compounds **A**, **6**, and **8** in the active site of Akt. Therefore, Homo sapiens Akt (PDB code: 4EJN) was chosen for docking studies related to its affinity with azole groups as reported by Ashwell et al. [35]. According to docking results, the cyanophenyl ring of compound **6** and the hydroxyphenyl ring of compound **8** presented π–π stacking interaction with the conserved Tyr272 residue of the binding site of Akt, just as previously determined as perpendicular to the core, forming a direct hydrophobic interaction with the same residue by Ashwell et al. [35]. Moreover, the thiosemicarbazone moiety of compound **A** was detected binding to Thr211 residue through hydrogen bonding interactions in the active site of Akt. Docked poses and docking interactions of compounds **A**, **6**, and **8** in the active site of Akt were depicted in Figure 3, Figure 4 and Figure 5, respectively.

All docking score (kcal/mol), glide gscore (kcal/mol), and glide emodel (kcal/mol) results of compounds **A**, **6**, and **8** were evaluated according to Schrodinger’s Maestro molecular modeling package programme (Schrödinger Release 2016-2: Schrödinger, LLC, New York, NY, USA) and these results are given in Table 5. Although the eModel score is appropriate for the comparison of different conformations of the same ligand but not for different ligands, the docking score is generally used for comparing different ligands [36]. Docking scores of compounds were calculated as −6.54, −5.08, and −5.45 kcal/mol for compounds **A**, **6**, and **8**, respectively. These docking results were fundamentally in agreement with the biological data.

As a part of this study, Molinspiration software was used to determine the physicochemical parameters (log P, TPSA, nrotb, molecular weight, number of hydrogen bond donors and acceptors, molecular volume) of the compounds for the evaluation of their compliance to Lipinski’s rule of five [37]. This rule states that most “drug like” molecules have logP ≤5, molecular weight ≤500, number of hydrogen bond acceptors ≤10, and number of hydrogen bond donors ≤5 [37,38,39,40]. Compounds violating more than one of these rules may have bioavailability problems. According to in silico studies, compounds **A** and **8** did not violate Lipinski’s rule, whereas other compounds only violated one parameter of Lipinski’s rule of five (Table 6). On the basis of Lipinski’s rule of five, they were expected to have good oral bioavailability.

## 3. Materials and Methods

### 3.1. Chemistry

All reagents purchased from commercial suppliers were used without further purification. The melting points (M.p.) of the compounds were determined on a MP90 digital melting point apparatus (Mettler Toledo, OH, USA) and are uncorrected. IR spectra were recorded on an IRPrestige-21 Fourier Transform Infrared spectrophotometer (Shimadzu, Tokyo, Japan). ^1^H-NMR and ^13^C-NMR spectra were recorded on a Varian Mercury-400 FT-NMR spectrometer (Agilent, Palo Alto, CA, USA). Splitting patterns are designated as follows: broad singlet (brs), singlet (s), doublet (d), doublet of doublets (dd), and multiplet (m). Mass spectra were recorded on an Agilent 6530 LC-MS/Q-TOF system (Agilent, Palo Alto, CA, USA). Thin Layer Chromatography (TLC) was performed on TLC Silica gel 60 F_254_ aluminium sheets (Merck, Darmstadt, Germany) to monitor the progress of the chemical reactions and check the purity of the synthesized compounds using petroleum ether: ethyl acetate (1:1) as an eluent.

### 3.2. General Procedure for the Synthesis of the Compounds

#### *4-(4-Cyanophenoxy)benzaldehyde thiosemicarbazone* (**A**)

A mixture of 4-(4-cyanophenoxy)benzaldehyde (0.025 mol) and thiosemicarbazide (0.025 mol) in ethanol (40 mL) was refluxed for 12 h. The reaction mixture was cooled and filtered. The product was crystallized from ethanol.

Ivory powder. M.p. 235–236 °C. Yield: 90%. IR ν_max_ (cm^−1^): 3446.79, 3319.49 (N-H stretching), 3149.76, 3097.68, 3008.95 (Aromatic C-H stretching), 2980.02 (Aliphatic C-H stretching), 2225.85 (C≡N stretching), 1585.49, 1537.27, 1490.97, 1458.18 (N-H bending, C=N and C=C stretching), 1413.82, 1361.74 (C-H bending), 1292.31, 1247.94, 1201.65, 1163.08, 1109.07, 1085.92, 1053.13, 1010.70 (C-N, C-O stretching and aromatic C-H in plane bending), 954.76, 927.76, 881.47, 869.90, 839.03, 819.75, 785.03, 707.88, 696.30, 646.15 (Aromatic C-H out of plane bending and C-S stretching). ^1^H-NMR (400 MHz, DMSO-*d_6_*) δ (ppm): 7.15 (d, *J* = 9.2 Hz, 4H), 7.87 (d, *J* = 8.8 Hz, 2H), 7.91 (d, *J* = 9.2 Hz, 2H), 8.05 (brs, 1H), 8.07 (s, 1H), 8.23 (brs, 1H), 11.46 (s, 1H). ^13^C-NMR (100 MHz, DMSO-*d_6_*) δ (ppm): 106.26 (C), 119.25 (2CH), 119.34 (C), 120.79 (2CH), 130.10 (2CH), 131.73 (C), 135.41 (2CH), 141.94 (CH), 156.51 (C), 161.22 (C), 178.64 (C).

#### *2-[2-((4-(4-Cyanophenoxy)phenyl)methylene)hydrazinyl]-4-arylthiazole derivatives* (**1**–**10**)

A mixture of compound **A** (0.001 mol) and aryl acyl bromide (0.001 mol) in ethanol (20 mL) was refluxed for 6 h. The reaction mixture was cooled and filtered. The product was crystallized from ethanol.

*2-[2-((4-(4-Cyanophenoxy)phenyl)methylene)hydrazinyl]-4-phenylthiazole* (**1**). Dark goldenrod powder. M.p. 214–215 °C. Yield: 74%. IR ν_max_ (cm^−1^): 3452.58 (N-H stretching), 3089.96 (Aromatic C-H stretching), 2987.74, 2900.94, 2883.58 (Aliphatic C-H stretching), 2229.71 (C≡N stretching), 1625.99, 1591.27, 1490.97, 1444.68 (N-H bending, C=N and C=C stretching), 1409.96, 1361.74 (C-H bending), 1303.88, 1240.23, 1205.51, 1165.00, 1089.78, 1047.35, 1028.06 (C-N, C-O stretching and aromatic C-H in plane bending), 879.54, 864.11, 835.18, 773.46, 729.09, 715.59, 688.59, 648.08 (Aromatic C-H out of plane bending and C-S stretching). ^1^H-NMR (400 MHz, DMSO-*d_6_*) δ (ppm): 7.08–7.41 (m, 8H), 7.72–7.86 (m, 6H), 8.06 (s, 1H), 12.01 (s, 1H). ^13^C-NMR (100 MHz, DMSO-*d_6_*) δ (ppm): 104.43 (CH), 106.20 (C), 119.17 (2CH), 119.36 (C), 121.11 (2CH), 126.24 (2CH), 128.62 (CH), 129.02 (2CH), 129.32 (2CH), 131.95 (C), 135.16 (C), 135.41 (2CH), 141.25 (CH), 150.90 (C), 156.03 (C), 161.31 (C), 168.90 (C). HRMS (ESI) (*m/z*): [M + H]^+^ 397.1121.

*2-[2-((4-(4-Cyanophenoxy)phenyl)methylene)hydrazinyl]-4-(4-nitrophenyl)thiazole* (**2**). Orange powder. M.p. 250–251 °C. Yield: 92%. IR ν_max_ (cm^−1^): 3305.99 (N-H stretching), 3113.11, 3062.96 (Aromatic C-H stretching), 2987.74, 2927.94 (Aliphatic C-H stretching), 2229.71 (C≡N stretching), 1593.20, 1573.91, 1494.83 (N-H bending, C=N and C=C stretching), 1431.18, 1408.04, 1355.96 (C-H bending), 1338.60 (NO_2_ stretching), 1315.45, 1278.81, 1236.37, 1201.65, 1168.86, 1124.50, 1111.00, 1097.50, 1047.35, 1010.70 (C-N, C-O stretching and aromatic C-H in plane bending), 929.69, 879.54, 858.32, 837.11, 815.89, 715.59 (Aromatic C-H out of plane bending). ^1^H-NMR (400 MHz, DMSO-*d_6_*) δ (ppm): 7.17 (d, *J* = 9.2 Hz, 2H), 7.21 (d, *J* = 9.2 Hz, 2H), 7.73–7.77 (m, 3H), 7.87 (d, *J* = 9.2 Hz, 2H), 8.09 (s, 1H), 8.11 (d, *J* = 9.2 Hz, 2H), 8.28 (d, *J* = 9.2 Hz, 2H), 12.33 (s, 1H). ^13^C-NMR (100 MHz, DMSO-*d_6_*) δ (ppm): 106.23 (C), 109.31 (CH), 119.18 (2CH), 119.35 (C), 121.10 (2CH), 124.81 (2CH), 127.02 (2CH), 129.07 (2CH), 131.82 (C), 135.41 (2CH), 141.34 (C), 141.54 (CH), 146.90 (C), 149.27 (C), 156.13 (C), 161.28 (C), 169.32 (C). HRMS (ESI) (*m/z*): [M + H]^+^ 442.0969.

*2-[2-((4-(4-Cyanophenoxy)phenyl)methylene)hydrazinyl]-4-(4-fluorophenyl)thiazole* (**3**). Cinnamon-colored powder. M.p. 204–205 °C. Yield: 77%. IR ν_max_ (cm^−1^): 3419.79 (N-H stretching), 3057.17 (Aromatic C-H stretching), 2914.44 (Aliphatic C-H stretching), 2225.85 (C≡N stretching), 1622.13, 1593.20, 1568.13, 1558.48, 1494.83, 1456.26 (N-H bending, C=N and C=C stretching), 1411.89 (C-H bending), 1290.38, 1238.30, 1203.58, 1166.93, 1105.21, 1083.99, 1037.70, 1014.56 (C-N, C-O stretching and aromatic C-H in plane bending), 881.47, 833.25, 804.32, 794.67, 759.95, 746.45, 715.59, 692.44 (Aromatic C-H out of plane bending and C-S stretching). ^1^H-NMR (400 MHz, DMSO-*d_6_*) δ (ppm): 7.10–7.34 (m, 8H), 7.71–7.77 (m, 2H), 7.83–7.91 (m, 3H), 8.08 (s, 1H), 12.31 (s, 1H). ^13^C-NMR (100 MHz, DMSO-*d_6_*) δ (ppm): 104.18 (CH), 106.19 (C), 116.13 (d, *J* = 21.3 Hz, 2CH), 119.15 (2CH), 119.34 (C), 121.10 (2CH), 128.21 (d, *J* = 7.6 Hz, 2CH), 129.01 (2CH), 131.91 (2C), 135.40 (2CH), 141.28 (CH), 149.91 (C), 156.03 (C), 161.28 (C), 163.52 (C), 168.98 (C). HRMS (ESI) (*m/z*): [M + H]^+^ 415.1047.

*2-[2-((4-(4-Cyanophenoxy)phenyl)methylene)hydrazinyl]-4-(4-chlorophenyl)thiazole* (**4**). Goldenrod powder. M.p. 227–228 °C. Yield: 83%. IR ν_max_ (cm^−1^): 3402.43 (N-H stretching), 3055.24 (Aromatic C-H stretching), 2912.51, 2858.51 (Aliphatic C-H stretching), 2225.85 (C≡N stretching), 1614.42, 1606.70, 1593.20, 1492.90 (N-H bending, C=N and C=C stretching), 1244.09, 1201.65, 1166.93, 1093.64, 1012.63 (C-N, C-O stretching and aromatic C-H in plane bending), 879.54, 829.39, 759.95, 746.45 (Aromatic C-H out of plane bending). ^1^H-NMR (400 MHz, DMSO-*d_6_*) δ (ppm): 7.17 (d, *J* = 9.2 Hz, 2H), 7.21 (d, *J* = 8.8 Hz, 2H), 7.41 (s, 1H), 7.47 (d, *J* = 8.4 Hz, 2H), 7.76 (d, *J* = 8.8 Hz, 2H), 7.88 (d, *J* = 8.8 Hz, 4H), 8.09 (s, 1H), 12.31 (s, 1H). ^13^C-NMR (100 MHz, DMSO-*d_6_*) δ (ppm): 105.23 (CH), 106.19 (C), 119.15 (2CH), 119.34 (C), 121.10 (2CH), 127.93 (2CH), 129.02 (2CH), 129.31 (2CH), 131.89 (C), 132.66 (C), 134.06 (C), 135.40 (2CH), 141.33 (CH), 149.78 (C), 156.04 (C), 161.28 (C), 169.02 (C). HRMS (ESI) (*m/z*): [M + H]^+^ 431.0751.

*2-[2-((4-(4-Cyanophenoxy)phenyl)methylene)hydrazinyl]-4-(4-bromophenyl)thiazole* (**5**). Dark beige powder. M.p. 217–218 °C. Yield: 88%. IR ν_max_ (cm^−1^): 3284.77 (N-H stretching), 3116.97, 3047.53 (Aromatic C-H stretching), 2227.78 (C≡N stretching), 1593.20, 1571.99, 1556.55, 1494.83, 1473.62 (N-H bending, C=N and C=C stretching), 1415.75, 1396.46, 1352.10 (C-H bending), 1284.59, 1244.09, 1203.58, 1166.93, 1114.86, 1097.50, 1072.42, 1045.42, 1006.84 (C-N, C-O stretching and aromatic C-H in plane bending), 954.76, 933.55, 908.47, 881.47, 854.47, 833.25, 825.53, 729.09, 696.30, 686.66, 663.51, 632.65 (Aromatic C-H out of plane bending and C-S stretching). ^1^H-NMR (400 MHz, DMSO-*d_6_*) δ (ppm): 7.17 (d, *J* = 9.2 Hz, 2H), 7.20 (d, *J* = 9.2 Hz, 2H), 7.41 (s, 1H), 7.61 (d, *J* = 8.4 Hz, 2H), 7.76 (d, *J* = 9.2 Hz, 2H), 7.81 (d, *J* = 8.4 Hz, 2H), 7.87 (d, *J* = 8.8 Hz, 2H), 8.08 (s, 1H), 12.31 (s, 1H). ^13^C-NMR (100 MHz, DMSO-*d_6_*) δ (ppm): 105.30 (CH), 106.19 (C), 119.14 (2CH), 119.34 (C), 121.09 (2CH), 121.24 (C), 128.23 (2CH), 129.01 (2CH), 131.90 (C), 132.21 (2CH), 134.46 (C), 135.39 (2CH), 141.27 (CH), 149.91 (C), 156.03 (C), 161.28 (C), 169.03 (C). HRMS (ESI) (*m/z*): [M + 2H]^+^ 477.0225.

*2-[2-((4-(4-Cyanophenoxy)phenyl)methylene)hydrazinyl]-4-(4-cyanophenyl)thiazole* (**6**). Metallic gold powder. M.p. 232–233 °C. Yield: 90%. IR ν_max_ (cm^−1^): 3329.14 (N-H stretching), 3122.75, 3095.75, 3045.60 (Aromatic C-H stretching), 2978.09 (Aliphatic C-H stretching), 2218.14 (C≡N stretching), 1604.77, 1593.20, 1560.41, 1492.90, 1483.26 (N-H bending, C=N and C=C stretching), 1409.96, 1354.03 (C-H bending), 1284.59, 1238.30, 1193.94, 1168.86, 1122.57, 1101.35, 1049.28, 1014.56 (C-N, C-O stretching and aromatic C-H in plane bending), 908.47, 881.47, 867.97, 839.03, 815.89, 794.67, 769.60, 736.81, 715.59, 696.30, 657.73, 642.30, 605.65, 543.93 (Aromatic C-H out of plane bending and C-S stretching). ^1^H-NMR (400 MHz, DMSO-*d_6_*) δ (ppm): 7.17 (d, *J* = 9.2 Hz, 2H), 7.21 (d, *J* = 8.8 Hz, 2H), 7.65 (s, 1H), 7.76 (d, *J* = 8.8 Hz, 2H), 7.87 (d, *J* = 9.2 Hz, 4H), 8.04 (d, *J* = 8.8 Hz, 2H), 8.09 (s, 1H), 12.29 (brs, 1H). ^13^C-NMR (100 MHz, DMSO-*d_6_*) δ (ppm): 106.22 (C), 108.27 (CH), 110.29 (C), 119.16 (2CH), 119.35 (C), 119.68 (C), 121.10 (2CH), 126.80 (2CH), 129.05 (2CH), 131.86 (C), 133.38 (2CH), 135.40 (2CH), 139.43 (C), 141.46 (CH), 149.53 (C), 156.08 (C), 161.28 (C), 169.22 (C). HRMS (ESI) (*m/z*): [M + H]^+^ 422.1092.

*2-[2-((4-(4-Cyanophenoxy)phenyl)methylene)hydrazinyl]-4-(4-methylphenyl)thiazole* (**7**). Pale yellow powder. M.p. 228–229 °C. Yield: 74%. IR ν_max_ (cm^−1^): 3412.08 (N-H stretching), 3051.39 (Aromatic C-H stretching), 2918.30, 2864.29 (Aliphatic C-H stretching), 2223.92 (C≡N stretching), 1593.20, 1564.27, 1494.83 (N-H bending, C=N and C=C stretching), 1435.04 (C-H bending), 1246.02, 1201.65, 1166.93, 1085.92, 1035.77, 1014.56 (C-N, C-O stretching and aromatic C-H in plane bending), 879.54, 856.39, 831.32, 819.75, 759.95, 746.45, 715.59, 694.37 (Aromatic C-H out of plane bending and C-S stretching). ^1^H-NMR (400 MHz, DMSO-*d_6_*) δ (ppm): 2.33 (s, 3H), 7.05–7.26 (m, 7H), 7.73–7.89 (m, 6H), 8.12 (s, 1H), 12.27 (s, 1H). ^13^C-NMR (100 MHz, DMSO-*d_6_*) δ (ppm): 21.50 (CH_3_), 103.61 (CH), 106.20 (C), 119.16 (2CH), 119.34 (C), 121.07 (2CH), 126.25 (2CH), 129.09 (2CH), 129.88 (2CH), 131.82 (C), 132.10 (C), 135.39 (2CH), 137.75 (C), 141.74 (CH), 150.27 (C), 156.09 (C), 161.25 (C), 168.85 (C). HRMS (ESI) (*m/z*): [M + H]^+^ 411.1300.

*2-[2-((4-(4-Cyanophenoxy)phenyl)methylene)hydrazinyl]-4-(4-hydroxyphenyl)thiazole* (**8**). Brown powder. M.p. 249–250 °C. Yield: 73%. IR ν_max_ (cm^−1^): 3444.87, 3361.93 (N-H stretching), 3165.19, 3115.04, 3037.89 (Aromatic C-H stretching), 2877.79 (Aliphatic C-H stretching), 2223.92 (C≡N stretching), 1624.06, 1593.20, 1512.19, 1492.90 (N-H bending, C=N and C=C stretching), 1435.04, 1371.39 (C-H bending), 1249.87, 1203.58, 1184.29, 1166.93, 1078.21, 1041.56, 1012.63 (C-N, C-O stretching and aromatic C-H in plane bending), 956.69, 879.54, 852.54, 829.39, 802.39, 736.81, 702.09, 651.94, 632.65, 599.86 (Aromatic C-H out of plane bending and C-S stretching). ^1^H-NMR (400 MHz, DMSO-*d_6_*) δ (ppm): 6.79 (d, *J* = 8.8 Hz, 2H), 7.05 (s, 1H), 7.15 (dd, *J* = 16.0, 8.4 Hz, 4H), 7.61 (d, *J* = 8.0 Hz, 2H), 7.74 (d, *J* = 8.8 Hz, 2H), 7.83 (d, *J* = 8.4 Hz, 2H), 8.13-8.14 (m, 1H), 9.21 (s, 1H), 12.30 (s, 1H). ^13^C-NMR (100 MHz, DMSO-*d_6_*) δ (ppm): 101.76 (CH), 106.24 (C), 116.08 (2CH), 119.21 (2CH), 119.34 (C), 121.04 (2CH), 125.33 (C), 127.91 (2CH), 129.29 (2CH), 131.56 (C), 135.40 (2CH), 143.30 (CH), 149.50 (C), 156.32 (C), 158.18 (C), 161.18 (C), 168.82 (C). HRMS (ESI) (*m/z*): [M + H]^+^ 413.1096.

*2-[2-((4-(4-Cyanophenoxy)phenyl)methylene)hydrazinyl]-4-(4-methoxyphenyl)thiazole* (**9**). Mustard-colored powder. M.p. 210–211 °C. Yield: 75%. IR ν_max_ (cm^−1^): 3151.69, 3061.03 (Aromatic C-H stretching), 2958.80, 2835.36 (Aliphatic C-H stretching), 2223.92 (C≡N stretching), 1593.20, 1562.34, 1494.83 (N-H bending, C=N and C=C stretching), 1435.04, 1413.82, 1365.60 (C-H bending), 1288.45, 1244.09, 1201.65, 1166.93, 1085.92, 1028.06, 1014.56 (C-N, C-O stretching and aromatic C-H in plane bending), 879.54, 858.32, 833.25, 748.38, 711.73, 692.44 (Aromatic C-H out of plane bending and C-S stretching). ^1^H-NMR (400 MHz, DMSO-*d_6_*) δ (ppm): 3.80 (s, 3H), 6.83 (d, *J* = 8.8 Hz, 1H), 6.99 (d, *J* = 8.4 Hz, 2H), 7.12–7.27 (m, 4H), 7.74–7.89 (m, 6H), 8.14 (s, 1H), 12.30 (s, 1H). ^13^C-NMR (100 MHz, DMSO-*d_6_*) δ (ppm): 55.06 (CH_3_), 101.67 (CH), 105.45 (C), 113.91 (2CH), 118.39 (2CH), 118.57 (C), 120.28 (2CH), 126.93 (2CH), 128.35 (2CH), 129.34 (C), 131.02 (C), 134.62 (2CH), 141.46 (CH), 149.27 (C), 155.80 (C), 159.08 (C), 160.60 (C), 168.03 (C). HRMS (ESI) (*m/z*): [M + H]^+^ 427.1254.

*2-[2-((4-(4-Cyanophenoxy)phenyl)methylene)hydrazinyl]-4-(naphthalen-2-yl)thiazole* (**10**). Vanilla powder. M.p. 204–205 °C. Yield: 88% IR ν_max_ (cm^−1^): 3381.21 (N-H stretching), 3118.90, 3059.10 (Aromatic C-H stretching), 2960.73, 2914.44, 2872.01 (Aliphatic C-H stretching), 2223.92 (C≡N stretching), 1620.21, 1595.13, 1566.20, 1492.90 (N-H bending, C=N and C=C stretching), 1440.83, 1361.74 (C-H bending), 1278.81, 1242.16, 1197.79, 1166.93, 1124.50, 1095.57, 1047.35, 1008.77 (C-N, C-O stretching and aromatic C-H in plane bending), 968.27, 958.62, 947.05, 918.12, 900.76, 866.04, 825.53, 802.39, 754.17, 713.66, 680.87, 650.01, 636.51, 609.51, 542.00 (Aromatic C-H out of plane bending and C-S stretching). ^1^H-NMR (400 MHz, DMSO-*d_6_*) δ (ppm): 7.16–7.24 (m, 4H), 7.48–7.58 (m, 3H), 7.76–7.82 (m, 2H), 7.84–7.98 (m, 5H), 8.02 (m, 1H), 8.12 (s, 1H), 8.40 (s, 1H), 12.30 (s, 1H). ^13^C-NMR (100 MHz, DMSO-*d_6_*) δ (ppm): 105.22 (CH), 106.21 (C), 119.17 (2CH), 119.37 (C), 121.12 (2CH), 124.63 (CH), 124.78 (CH), 126.71 (C), 127.14 (CH), 128.27 (CH), 128.80 (CH), 128.85 (CH), 129.02 (2CH), 131.98 (C), 132.73 (CH), 133.14 (C), 133.85 (C), 135.41 (2CH), 141.19 (CH), 151.10 (C), 156.03 (C), 161.31 (C), 168.98 (C). HRMS (ESI) (*m/z*): [M + H]^+^ 447.1317.

### 3.3. Biochemistry

#### 3.3.1. Cell Culture and Drug Treatment

C6 Rat glioma and NIH/3T3 mouse embryonic fibroblast cells were incubated in Dulbecco’s Modified Eagle’s Medium (DMEM) (Sigma, Deisenhofen, Germany) supplemented with 10% fetal calf serum (Gibco, Paisley, UK). A549 Human lung adenocarcinoma cells were incubated in 90% RPMI supplemented with 10% fetal bovine serum (Gibco, Paisley, UK). All media were supplemented with 100 IU/mL penicillin-streptomycin (Gibco, Paisley, UK) and the cells were incubated at 37 °C in a humidified atmosphere of 95% air and 5% CO_2_. Exponentially growing cells were plated at 2 × 10^4^ cells/mL into 96-well microtiter tissue culture plates (Nunc, Roskilde, Denmark) and incubated for 24 h before the addition of the drugs (the optimum cell number for cytotoxicity assays was determined in preliminary experiments). The stock solutions of the compounds were prepared in dimethyl sulfoxide (DMSO; Sigma Aldrich, Poole, UK) and further dilutions were made with fresh culture medium (the concentration of DMSO in the final culture medium was <0.1% which had no effect on the cell viability).

#### 3.3.2. MTT Assay

The level of cellular 3-(4,5-dimethylthiazol-2-yl)-2,5-diphenyltetrazolium bromide (MTT) (Sigma-Aldrich, St. Louis, MO, USA) reduction was quantified as previously described in the literature [41] with small modifications [42]. After 24 h of preincubation, the tested compounds and cisplatin (positive control) were added to give final concentration in the range 3.9–500 µg/mL and the cells were incubated for 24 h. At the end of this period, MTT was added to a final concentration of 0.5 mg/mL and the cells were incubated for 4 h at 37 °C. After the medium was removed, the formazan crystals formed by MTT metabolism were solubilized by addition of 200 µL DMSO to each well and absorbance was read at 540 nm with a microtiter plate spectrophotometer (BioTek Plate Reader, Winooski, VT, USA). Every concentration was repeated in three wells. IC_50_ values were defined as the drug concentrations that reduced absorbance to 50% of control values.

#### 3.3.3. Flow Cytometric Analyses of Apoptosis

After the cells were incubated with the most potent antiproliferative agents in this series at IC_50_ concentrations, phosphatidylserine externalization, which indicates early apoptosis, was measured by the FITC Annexin V apoptosis detection kit (BD Pharmingen, San Jose, CA, USA) on a BD FACSAria flow cytometer for 24 h. Annexin V staining protocol was applied according to the manufacturer’s instructions (BD Pharmingen, San Jose, CA, USA). The cells were then briefly washed with cold phosphate buffer saline (PBS) and suspended in a binding buffer at a concentration of 1 × 10^6^ cells/mL. Then, 100 µL of this solution containing 1 × 10^5^ cells was transferred to a 5 mL test tube. After 5 µL of Annexin V and PI was added, the cells were incubated for 15 min at room temperature in the dark. Then 400 µL of 1× binding buffer was added to each tube and the cells were processed for data acquisition, and analyzed on a BD FACSAria flow cytometer using FACSDiva version 6.1.1 software (BD Biosciences, San Jose, CA, USA).

#### 3.3.4. Determination of Akt Inhibition

After 10,000 cells/well were incubated with compounds **A**, **6**, **7**, and **8** and cisplatin at IC_50_ concentrations for 24 h, in cell ELISA colorimetric AKT activity protocol was applied according to the manufacturer’s instructions (Thermo Fisher Scientific, Waltham, MA, USA). Briefly, the media was removed and 100 µL of 4% formaldehyde was added to each well. The plate was incubated in a fume hood at room temperature for 15 min. Formaldehyde was removed and the plate was washed twice with 100 µL/well of 1× TBS. Then, 1× TBS was removed, 100 µL/well of 1× permeabilization buffer was added and incubated for 15 min at room temperature. The permeabilization buffer was removed and the plate was washed once with 100 µL/well of 1× TBS. Then, 1× TBS was removed, 100 µL/well quenching solution was added and incubated at room temperature for 20 min. Quenching solution was removed and the plate was washed once with 100 µL/well of 1× TBS. Then, 1× TBS was removed and 100 µL/well of blocking buffer was added and incubated at room temperature for 30 min. After the blocking buffer was removed, 50 µL/well of primary antibody was added. A plate sealer was applied and incubated overnight at 4 °C. The primary antibody solution was removed and the plate was washed three times with 100 µL/well of 1× wash Buffer. After the wash buffer was removed, 100 µL/well of diluted HRP conjugate was added and incubated for 30 min at room temperature. The wash buffer was removed and 100 µL/well of TMB substrate was added. The plate was then incubated at room temperature, protected from light. 100 µL/well of TMB stop solution was added and the absorbance was measured at 450 nm within 30 min of stopping the reaction. The experiment was performed in triplicate wells. The values of blank wells were subtracted from each well of treated and control cells. Percent Akt activity was defined as the relative absorbance of treated versus untreated control cells.

#### 3.3.5. Statistical Analyses

Statistical Package for the Social Sciences (SPSS) for Windows 15.0 was used for statistical analysis. Data was expressed as Mean ± SD. Comparisons were performed by one way ANOVA test for normally distributed continuous variables and post hoc analyses of group differences were expressed by the Tukey test.

### 3.4. Molecular Docking Studies

Compounds **A**, **6**, and **8** were docked to the active site of Akt enzyme. Ligands were set to the physiological pH (pH = 7.4) at the protonation step and the crystal structure of Akt enzyme was retrieved from Protein Data Bank server (PDB code: 4EJN). The structures of compounds **A**, **6**, and **8** were submitted in protein preparation module of Schrodinger’s Maestro molecular modeling package (Schrödinger Release 2016-2: Schrödinger, LLC, New York, NY, USA). In molecular docking simulations, Glide/XP docking protocols were applied for the prediction of topologies of compounds **A**, **6**, and **8** in the active site of Akt [35].

### 3.5. Molinspiration Calculations

The physicochemical parameters (log P, TPSA, nrotb, molecular weight, number of hydrogen bond donors and acceptors, molecular volume) of the compounds were calculated using Molinspiration software [37,38,39,40].

## 4. Conclusions

In the current work, the design, synthesis, and in vitro and in silico evaluation of a series of thiazolyl hydrazone derivatives as potential antitumor agents targeting Akt were described. Compound **6** was identified as the most potent and selective anticancer agent on A549 and C6 cells in this series. Moreover, compound **6** induced early and late apoptosis in C6 cell line more than cisplatin and showed significant Akt inhibitory activity. According to in vitro and in silico studies, compound **6** stands out as a potential orally bioavailable anticancer agent for further in vivo studies.

## Figures and Tables

**Table 1:** IC_50_ values of the compounds against A549, C6, and NIH/3T3 cells for 24 h.

Compound	IC_50_ (µg/mL)		
	A549 Cell Line	C6 Cell Line	NIH/3T3 Cell Line
**A**	92.5 ± 3.54	26.33 ± 1.53	225 ± 18.03
**1**	410 ± 14.14	>500	196.67 ± 20.82
**2**	100 ± 20	>500	71.67 ± 24.66
**3**	193.33 ± 15.28	>500	170 ± 36.06
**4**	206.67 ± 23.09	>500	180 ± 10
**5**	198.33 ± 10.41	>500	163.33 ± 5.77
**6**	12 ± 1.73	3.83 ± 0.76	>500
**7**	270 ± 95.39	16 ± 5.66	185 ± 5
**8**	106.67 ± 5.77	5.83 ± 0.76	226.67 ± 11.55
**9**	233.33 ± 57.74	>500	433.33 ± 28.87
**10**	>500	>500	>500
**Cisplatin**	17.33 ± 2.08	12.67 ± 3.06	ND

ND: Not determined.

**Table 2:** Percents of typical quadrant analysis of Annexin V FITC/Propidium iodide flow cytometry of A549 cells treated with compounds **A**, **6**, **8**, and cisplatin.

Groups	Early Apoptotic Cells%	Late Apoptotic Cells%	Viable Cells%
Control (untreated)	0.9	0.3	94.0
Compound **A** treated cells	7.6	45.9	24.2
Compound **6** treated cells	2.3	36.5	14.4
Compound **8** treated cells	2.1	38.7	18.5
Cisplatin treated cells	2.6	63.5	14.1

A549 cells were cultured for 24 h in medium with compounds **A**, **6**, **8**, and cisplatin at IC_50_ values. At least 10,000 cells were analyzed per sample, and quadrant analysis was performed.

**Table 3:** Percentages of typical quadrant analysis of Annexin V FITC/Propidium iodide flow cytometry of C6 cells treated with compounds **A**, **6**, **7**, **8**, and cisplatin.

Groups	Early Apoptotic Cells%	Late Apoptotic Cells%	Viable Cells%
Control (untreated)	2.5	5.5	82.7
Compound **A** treated cells	3.2	13.1	70.2
Compound **6** treated cells	4.6	28.2	42.2
Compound **7** treated cells	3.2	14.3	69.2
Compound **8** treated cells	1.9	23.8	49.7
Cisplatin treated cells	3.3	25.5	47.6

C6 cells were cultured for 24 h in medium with compounds **A**, **6**, **7**, **8**, and cisplatin at IC_50_ values. At least 10,000 cells were analyzed per sample, and quadrant analysis was performed.

**Table 4:** Akt inhibitory effects of compounds **A, 6, 7** and **8**.

Compound	Inhibition %	
	A549 Cell Line	C6 Cell Line
**A**	68.08 ± 2.48	25.23 ± 8.62
**6**	45.77 ± 10.58	71.66 ± 4.09
**7**	-	70.42 ± 10.37
**8**	57.37 ± 17.30	24.99 ± 0.52
**Cisplatin**	31.01 ± 3.18	77.25 ± 5.75

**Table 5:** Docking score (kcal/mol), glide gscore (kcal/mol), and glide emodel (kcal/mol) results of compounds **A**, **6**, and **8** for Akt enzyme (PDB code: 4EJN).

Compound	4EJN		
	Docking Score	Glide Gscore	Glide Emodel
**A**	−6.54	−6.54	−55.88
**6**	−5.08	−7.00	−71.40
**8**	−5.45	−6.81	−69.00

**Table 6:** Pharmacokinetic parameters important for bioavailability of compounds **1**-**10.**

Compound	Molecular Properties ^a^							
	MW	logP	TPSA	nrotb	HBA	HBD	Volume	Violation
**A**	296.36	3.39	83.44	5	5	3	256.11	0
**1**	396.48	5.47	70.31	6	5	1	346.33	1
**2**	441.47	5.42	116.13	7	8	1	369.67	1
**3**	414.46	5.63	70.31	6	5	1	351.26	1
**4**	430.92	6.14	70.31	6	5	1	359.87	1
**5**	475.37	6.28	70.31	6	5	1	364.22	1
**6**	421.49	5.22	94.10	6	6	1	363.19	1
**7**	410.50	5.92	70.31	6	5	1	362.89	1
**8**	412.47	4.99	90.54	6	6	2	354.35	0
**9**	426.50	5.52	79.54	7	6	1	371.88	1
**10**	446.54	6.65	70.31	6	5	1	390.32	1

^a^ Molecular properties were calculated using Molinspiration software. MW: Molecular weight; logP: The logarithm of octanol/water partition coefficient, TPSA: Topological polar surface area; nrotb: Number of rotatable bonds, HBA: Number of hydrogen bond acceptors, HBD: Number of hydrogen bond donors.

## Supplementary Materials

The following are available online. Figure S1: IR Spectrum of compound **A**; Figure S2: ^1^H NMR Spectrum of compound **A**; Figure S3: ^13^C NMR Spectrum of compound **A**; Figure S4: ^13^C NMR (DEPT) Spectrum of compound **A**; Figure S5: IR Spectrum of compound **1**; Figure S6: ^1^H NMR Spectrum of compound **1**; Figure S7: ^13^C NMR Spectrum of compound **1**; Figure S8: HRMS Spectrum of compound **1**; Figure S9: IR Spectrum of compound **2**; Figure S10: ^1^H NMR Spectrum of compound **2**; Figure S11: ^13^C NMR Spectrum of compound **2**; Figure S12: HRMS Spectrum of compound **2**; Figure S13: IR Spectrum of compound **3**; Figure S14: ^1^H NMR Spectrum of compound **3**; Figure S15: ^13^C NMR Spectrum of compound **3**; Figure S16: HRMS Spectrum of compound **3**; Figure S17: IR Spectrum of compound **4**; Figure S18: ^1^H NMR Spectrum of compound **4**; Figure S19: ^13^C NMR Spectrum of compound **4**; Figure S20: HRMS Spectrum of compound **4**; Figure S21: IR Spectrum of compound **5**; Figure S22: ^1^H NMR Spectrum of compound **5**; Figure S23: ^13^C NMR Spectrum of compound **5**; Figure S24: HRMS Spectrum of compound **5**; Figure S25: IR Spectrum of compound **6**; Figure S26: ^1^H NMR Spectrum of compound **6**; Figure S27: ^13^C NMR Spectrum of compound **6**; Figure S28: ^13^C NMR (DEPT) Spectrum of compound **6**; Figure S29: HRMS Spectrum of compound **6**; Figure S30: IR Spectrum of compound **7**; Figure S31: ^1^H NMR Spectrum of compound **7**; Figure S32: ^13^C NMR Spectrum of compound **7**; Figure S33: ^13^C NMR (DEPT) Spectrum of compound **7**; Figure S34: HRMS Spectrum of compound **7**; Figure S35: IR Spectrum of compound **8**; Figure S36: ^1^H NMR Spectrum of compound **8**; Figure S37: ^13^C NMR Spectrum of compound **8**; Figure S38: HRMS Spectrum of compound **8**; Figure S39: IR Spectrum of compound **9**; Figure S40: ^1^H NMR Spectrum of compound **9**; Figure S41: ^13^C NMR Spectrum of compound **9**; Figure S42: HRMS Spectrum of compound **9**; Figure S43: IR Spectrum of compound **10**; Figure S44: ^1^H NMR Spectrum of compound **10**; Figure S45: ^13^C NMR Spectrum of compound **10**; Figure S46: HRMS Spectrum of compound **10**.
