# Supplementary material for: Design, Synthesis, and Evaluation of a New Series of Thiazole-Based Anticancer Agents as Potent Akt Inhibitors
Source: Molecules. 2018 May 31;23(6):1318. doi: 10.3390/molecules23061318 (PMC6100633; doi:10.3390/molecules23061318)
Supplement: Supplementary file 1 [file molecules-23-01318-s001.pdf]

Supplementary Materials

Design, Synthesis and Evaluation of A New Series of Thiazole-Based Anticancer Agents as Potent Akt Inhibitors

Mehlika Dilek Altıntop [1,*], Belgin Sever [1], Gülşen Akalın Çiftçi [2], Ahmet Özdemir [1]

[1] Department of Pharmaceutical Chemistry, Faculty of Pharmacy, Anadolu University, 26470 Eskişehir, Turkey; belginsever@anadolu.edu.tr (B.S.); ahmeto@anadolu.edu.tr (A.Ö.)
[2] Department of Biochemistry, Faculty of Pharmacy, Anadolu University, 26470 Eskişehir, Turkey; gakalin@anadolu.edu.tr (G.A.Ç.)
* Correspondence: mdaltintop@anadolu.edu.tr; Tel.: +90-222-335-0580 (ext. 3772) (M.D.A.)

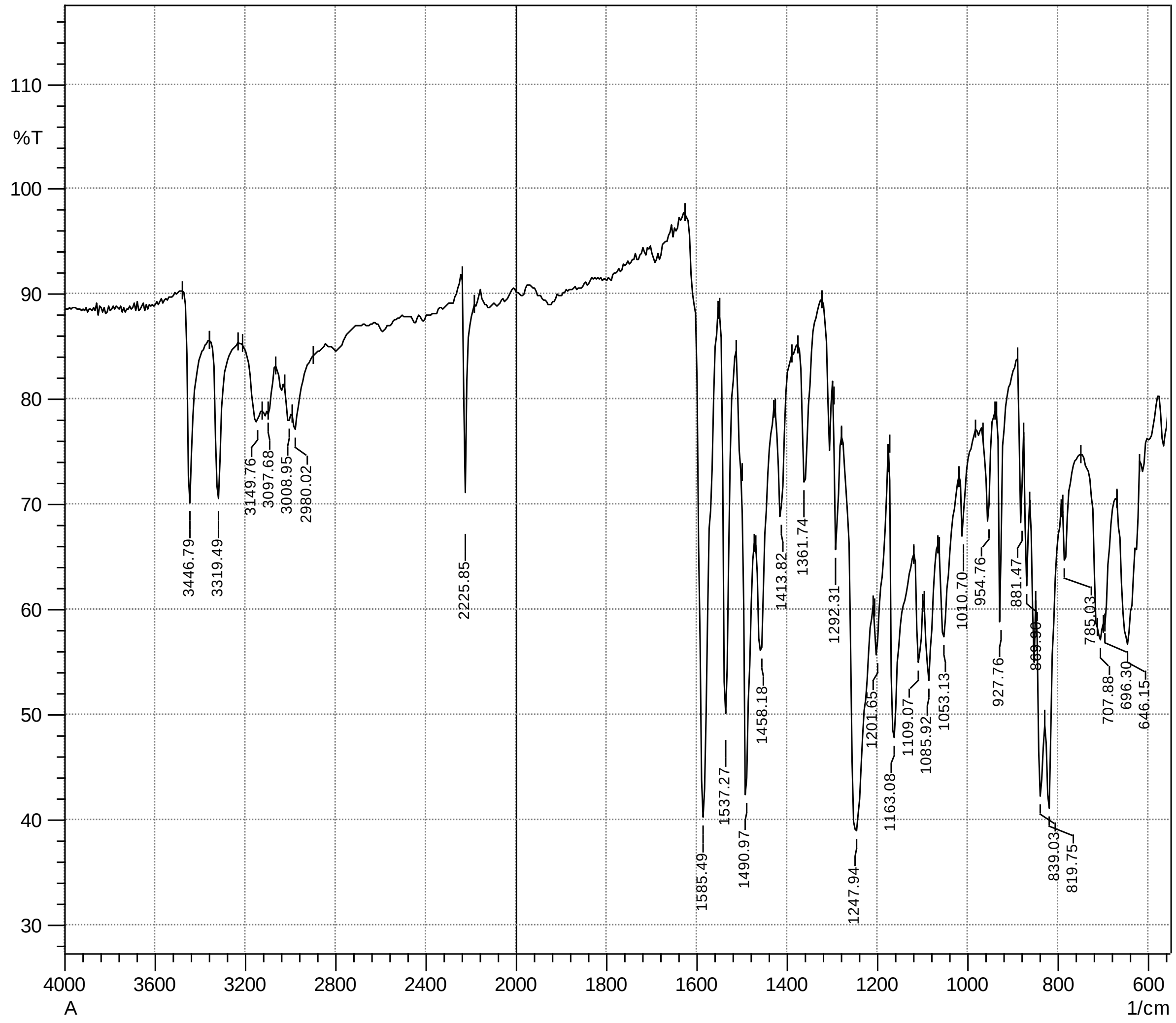

Figure S1. IR Spectrum of compound A

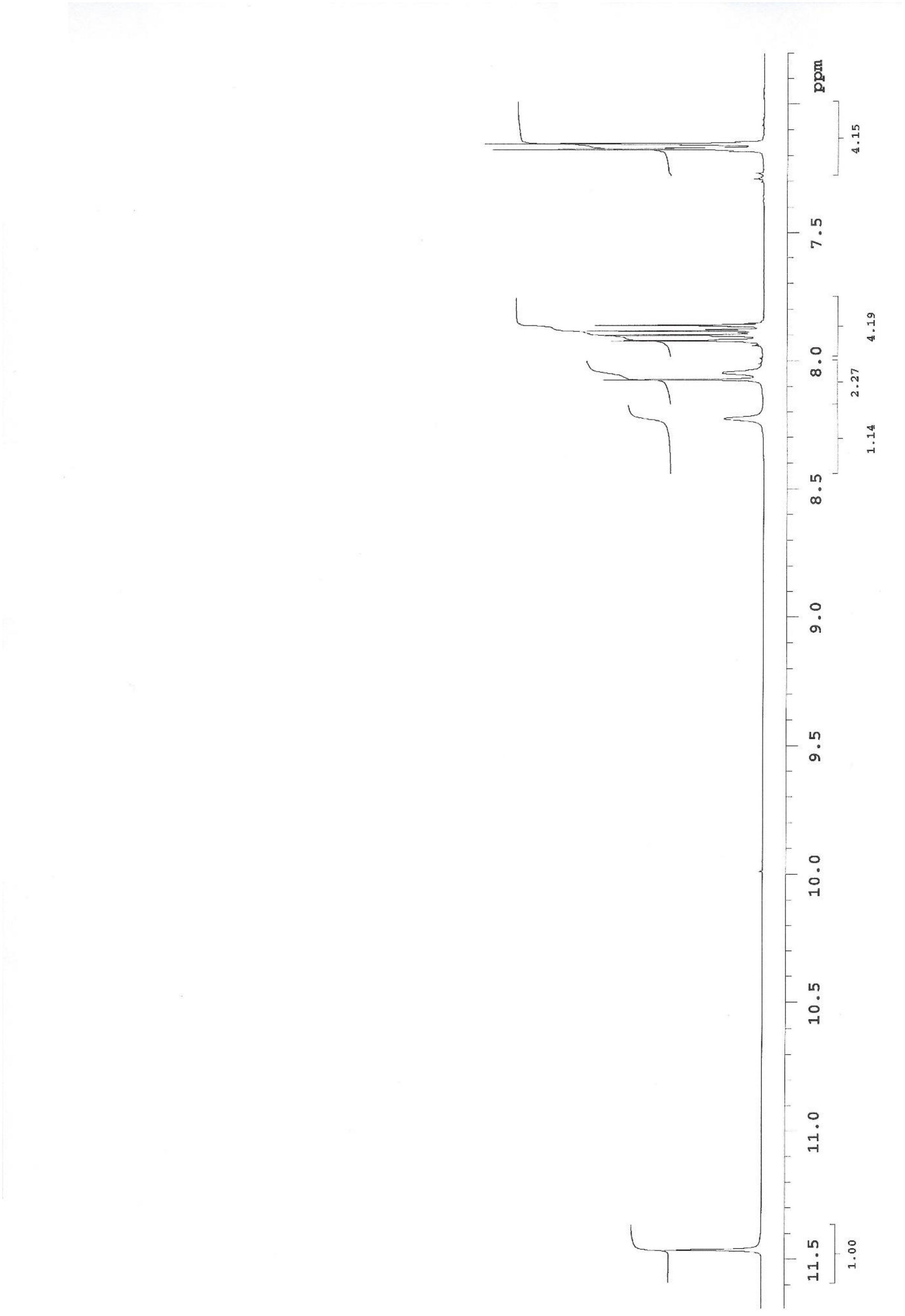

Figure S2. ^{1}H NMR Spectrum of compound **A**

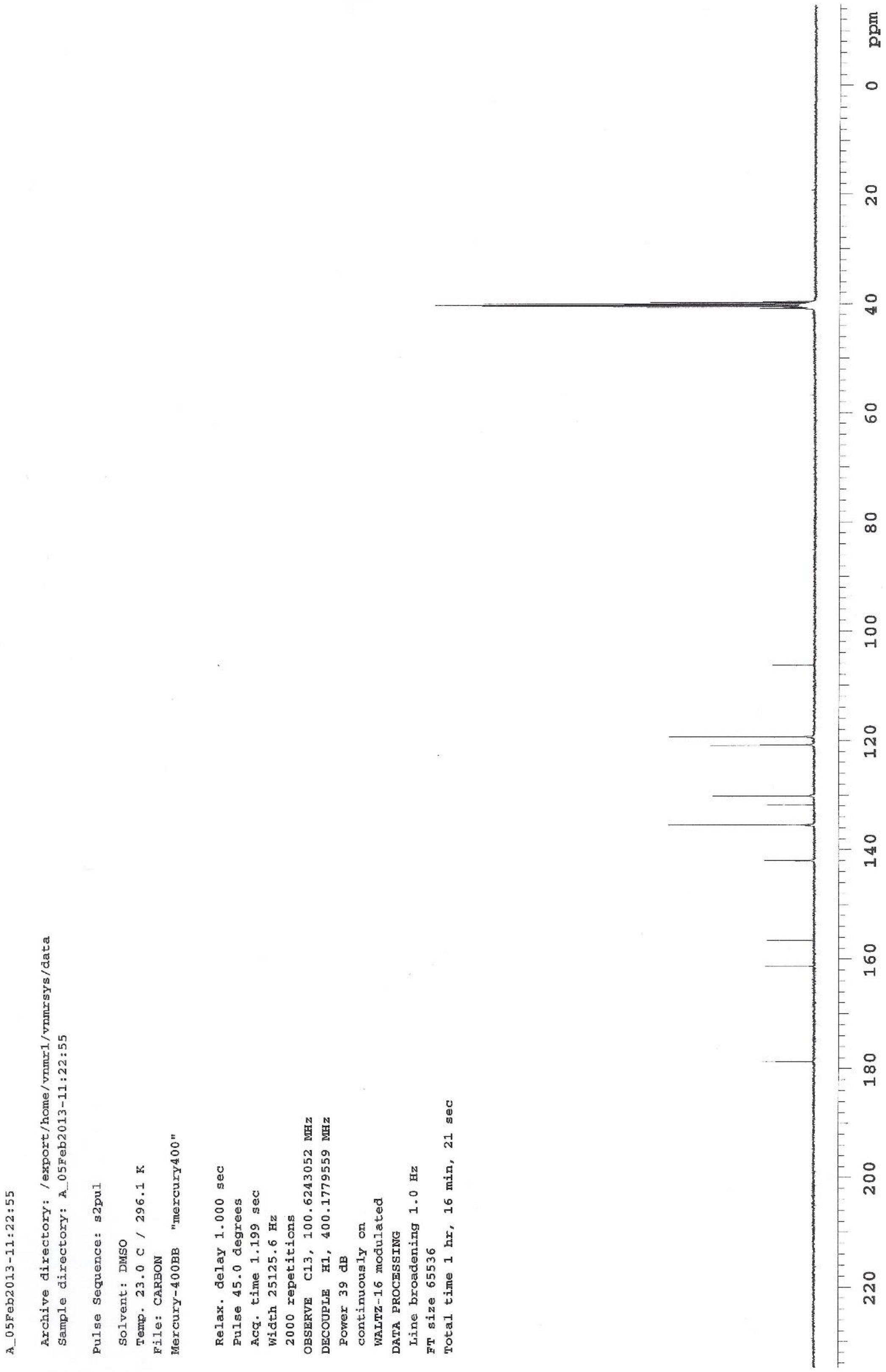

Figure S3. ^{13}C NMR Spectrum of compound **A**

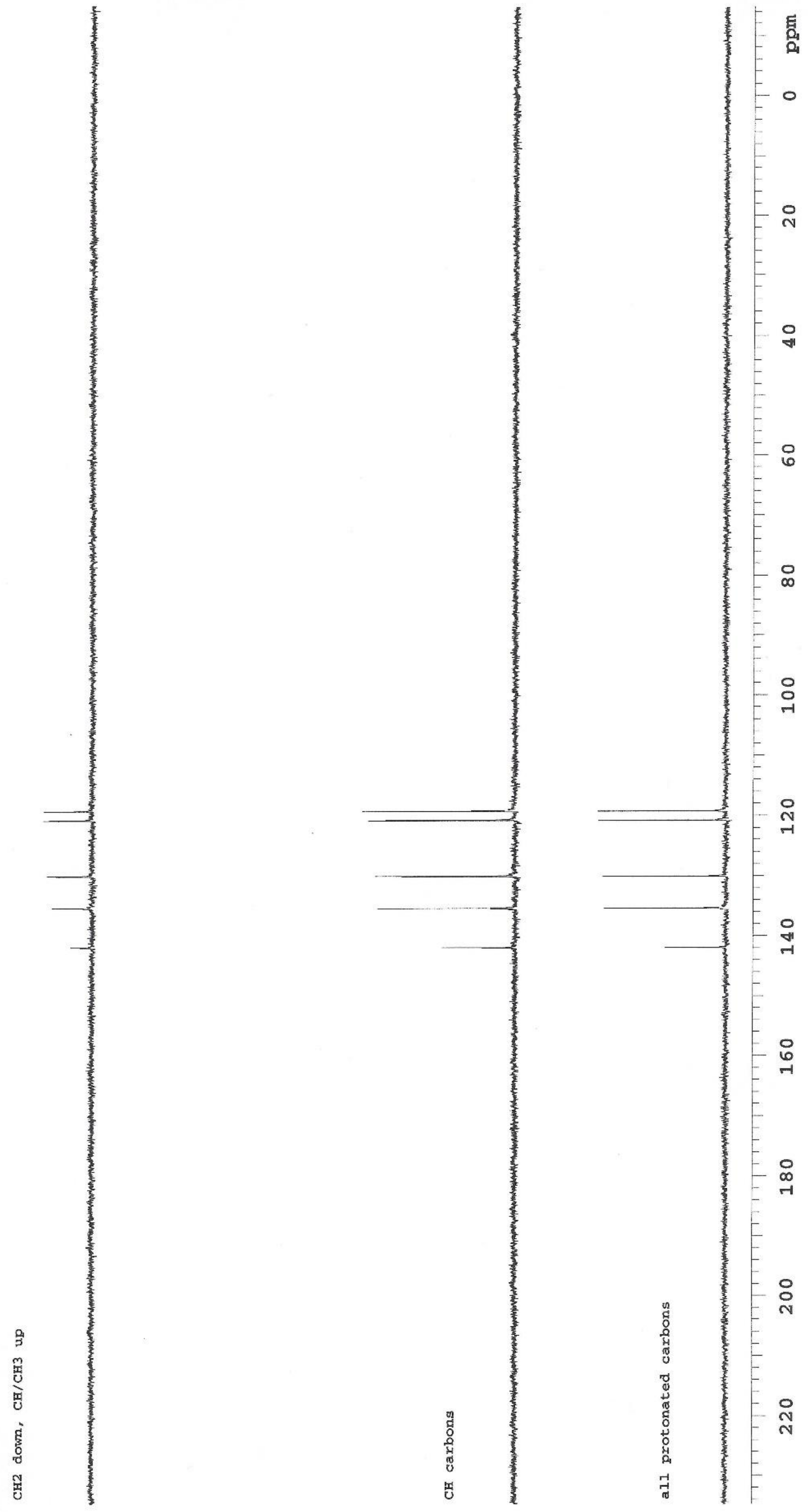

Figure S4. ^{13}C NMR (DEPT) Spectrum of compound **A**

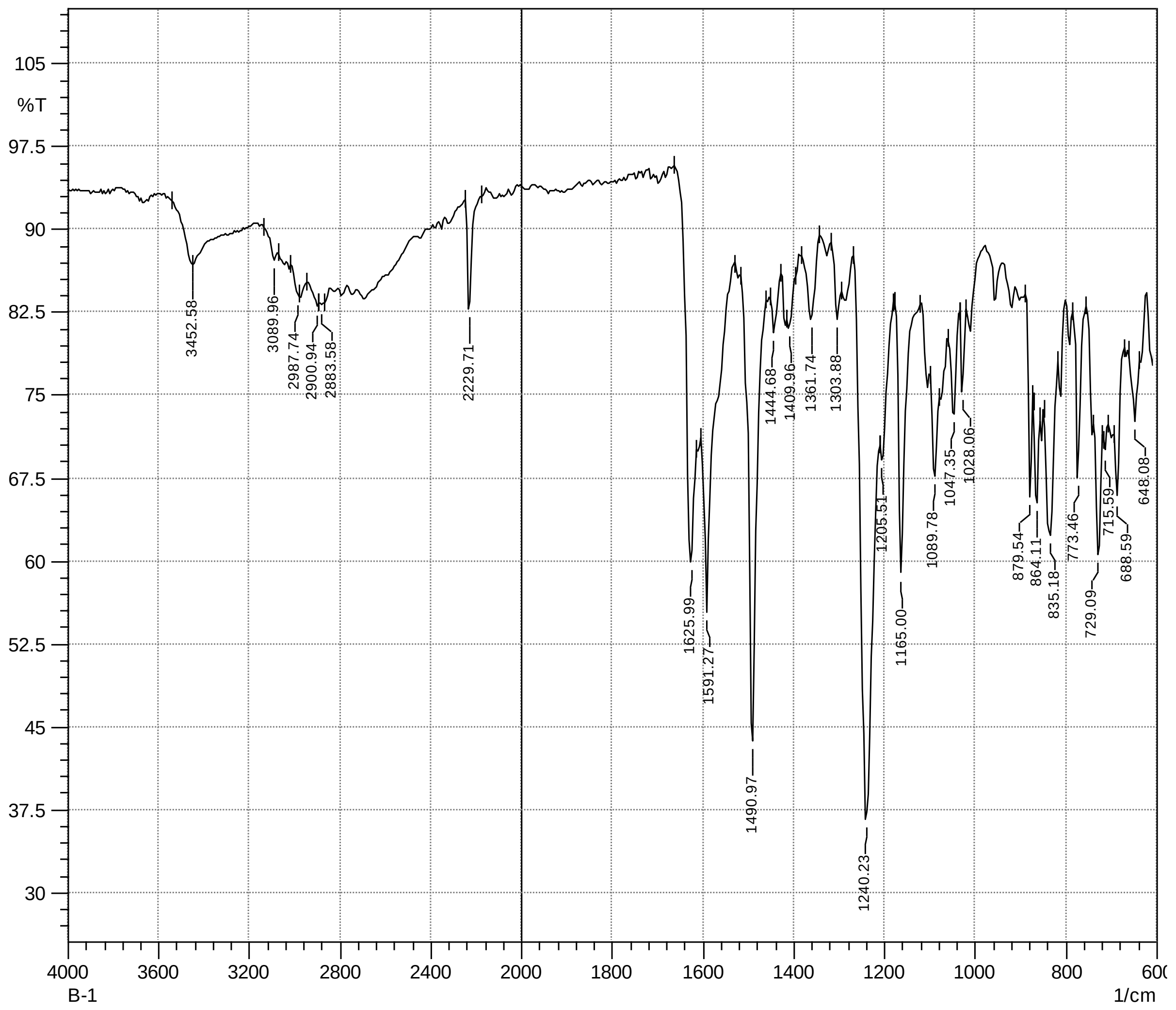

Figure S5. IR Spectrum of compound **1**

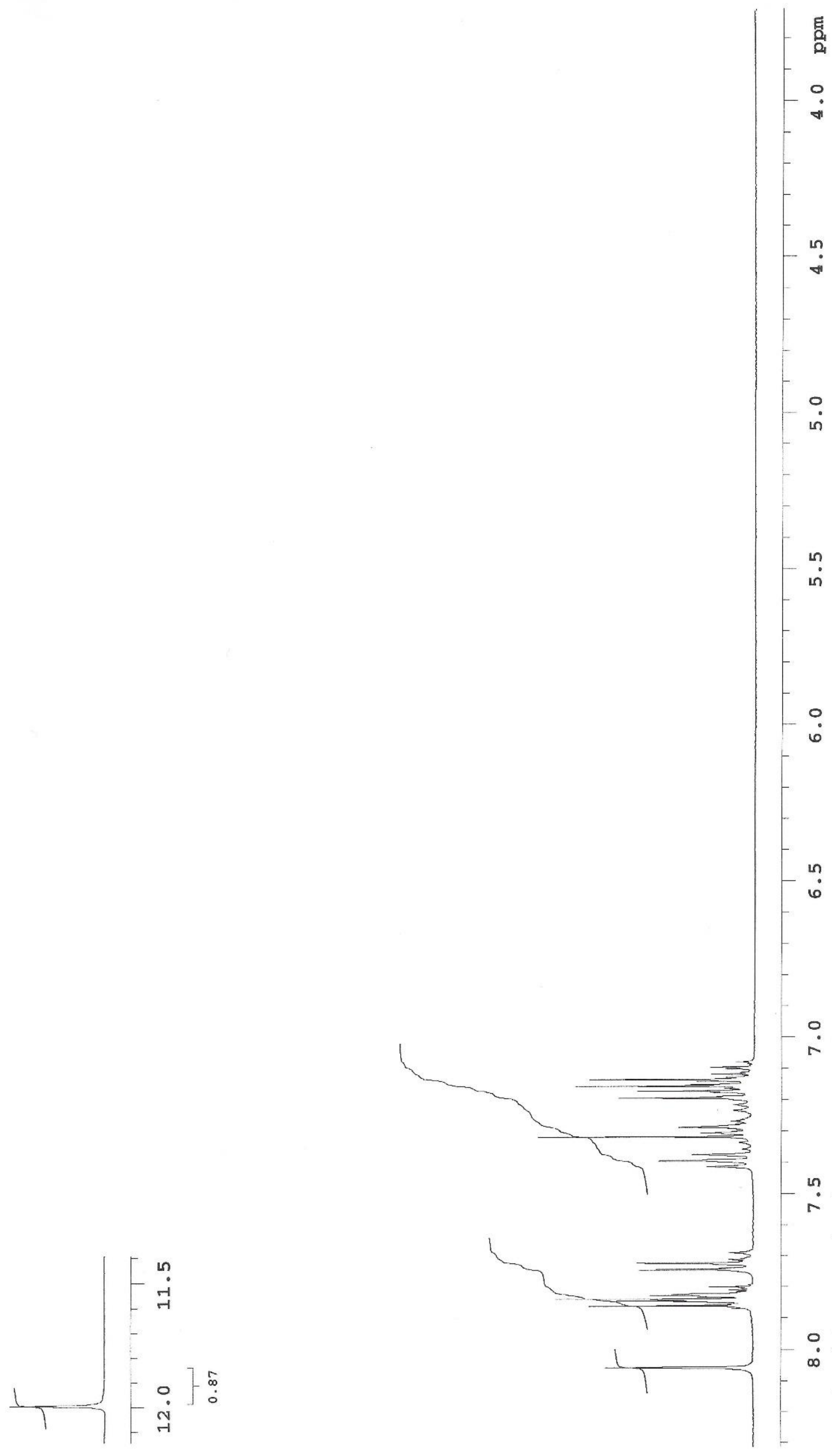

Figure S6. ^{1}H NMR Spectrum of compound **1**

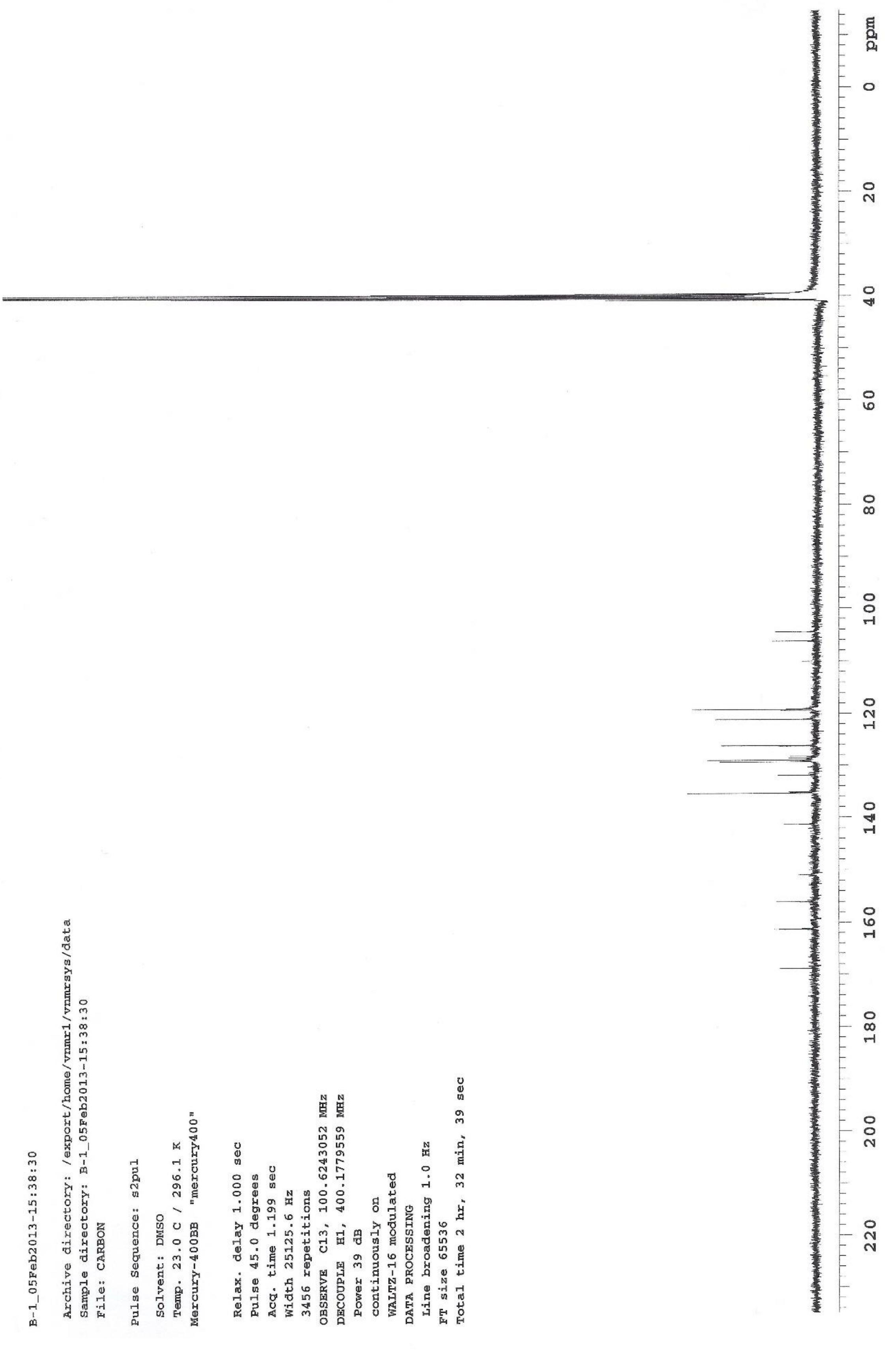

Figure S7. ^{13}C NMR Spectrum of compound **1**

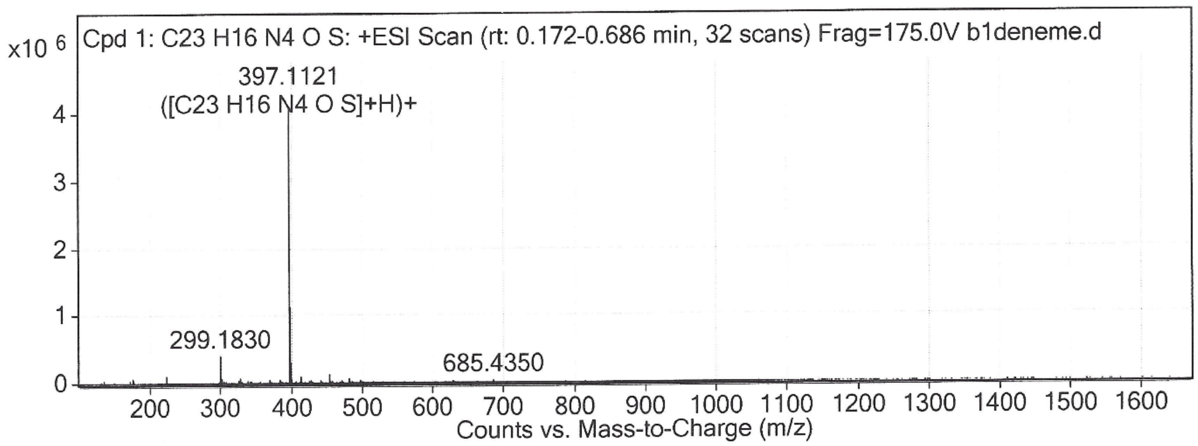

Figure S8. HRMS Spectrum of compound **1**

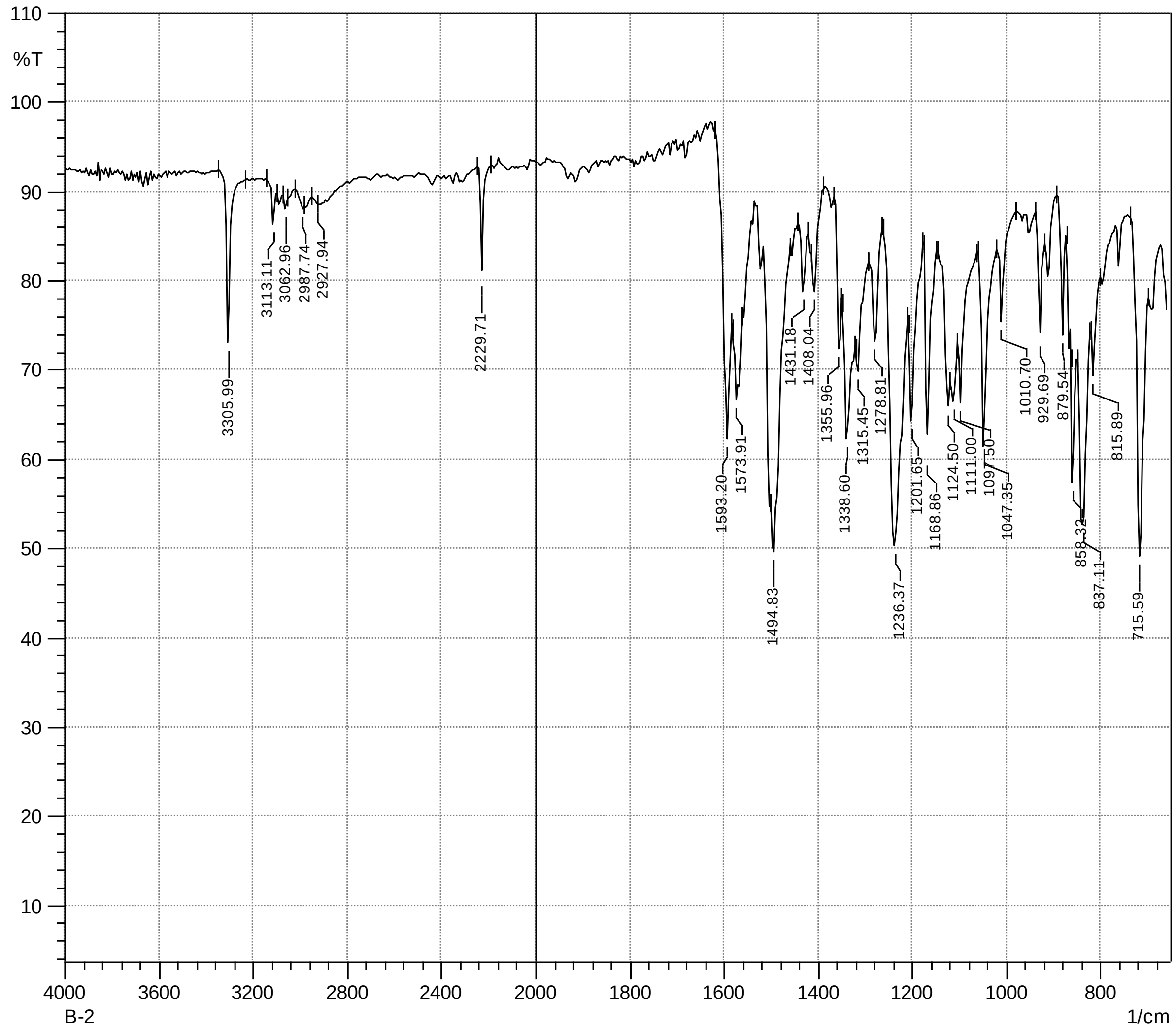

Figure S9. IR Spectrum of compound **2**

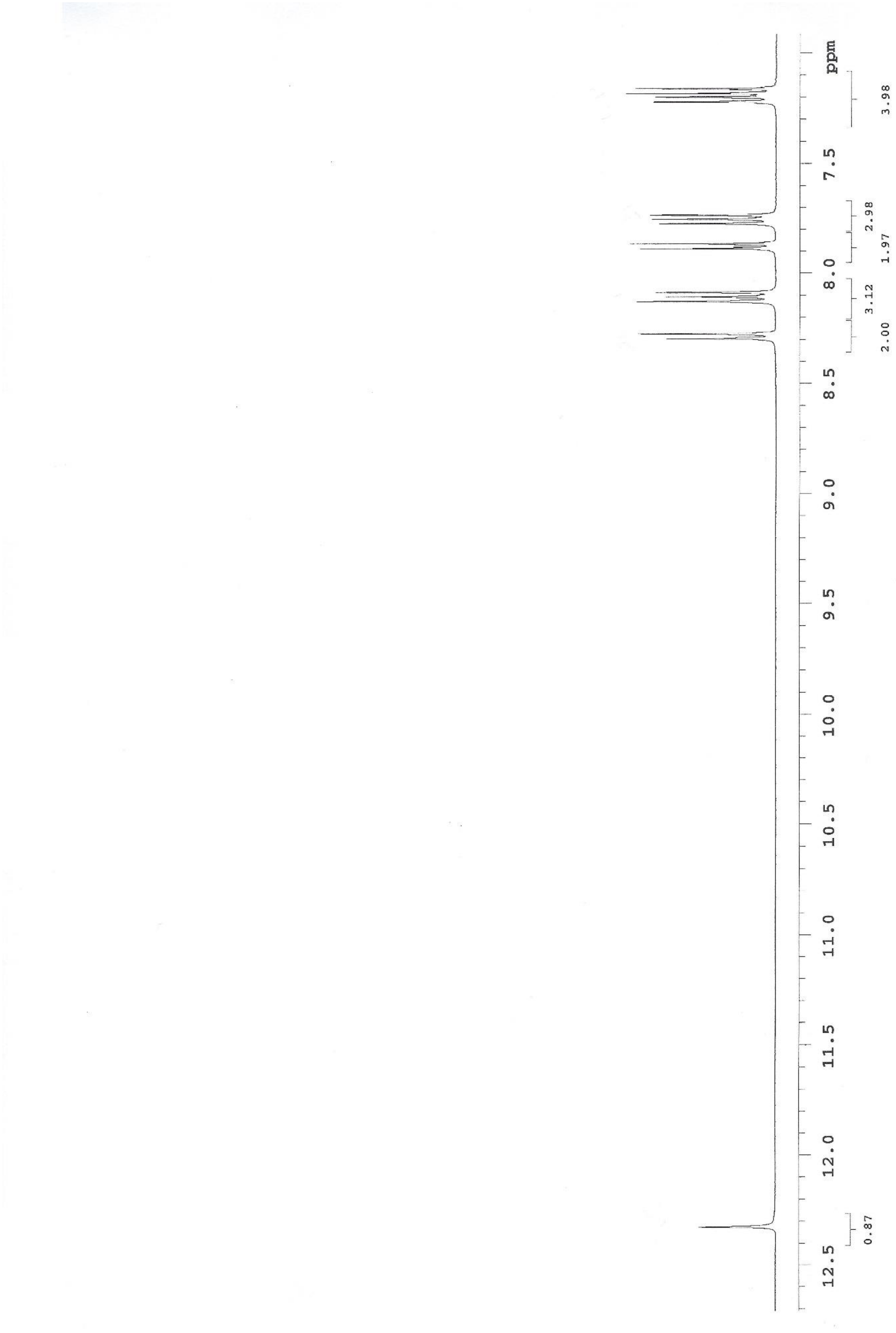

Figure S10. ^{1}H NMR Spectrum of compound **2**

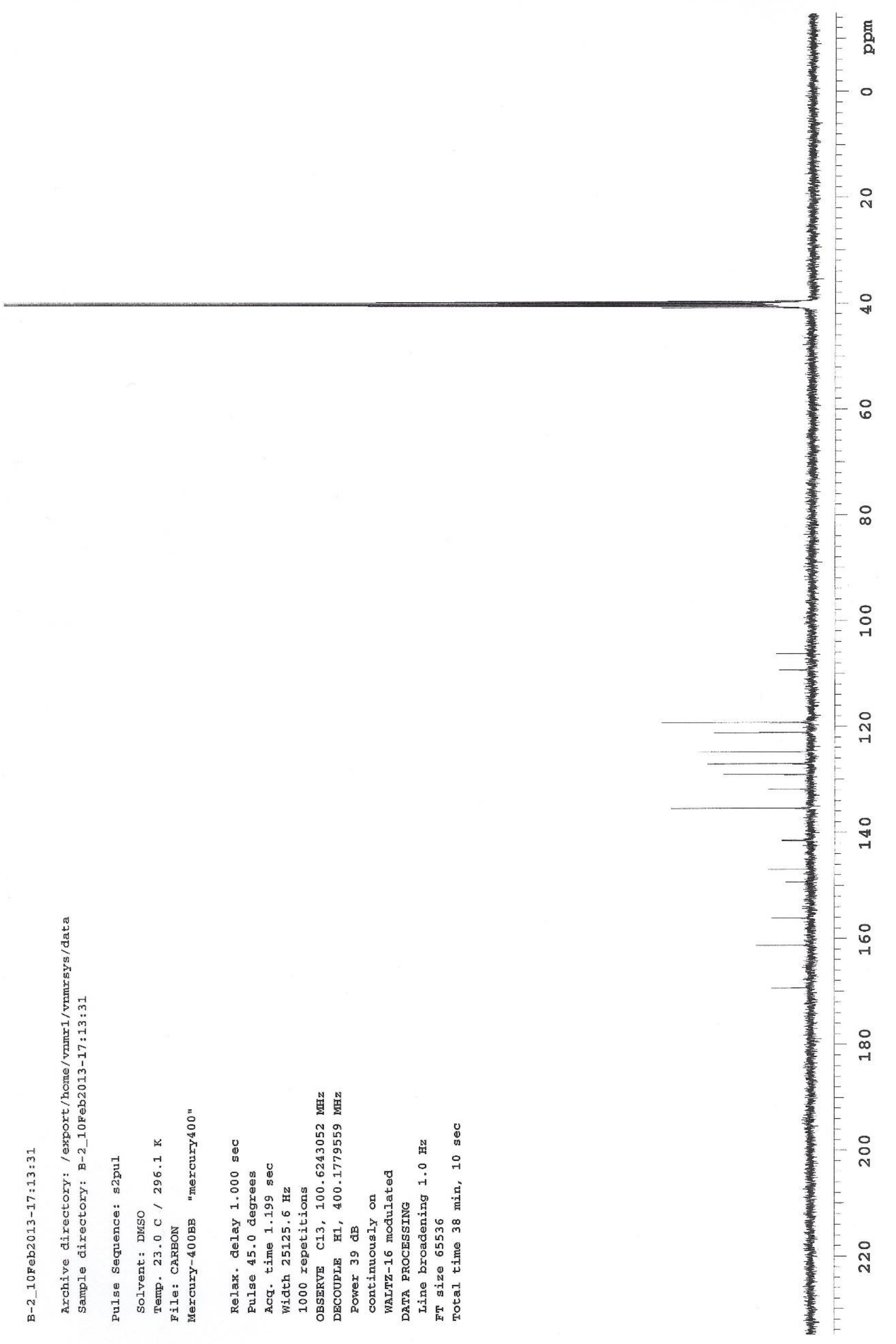

Figure S11. ^{13}C NMR Spectrum of compound **2**

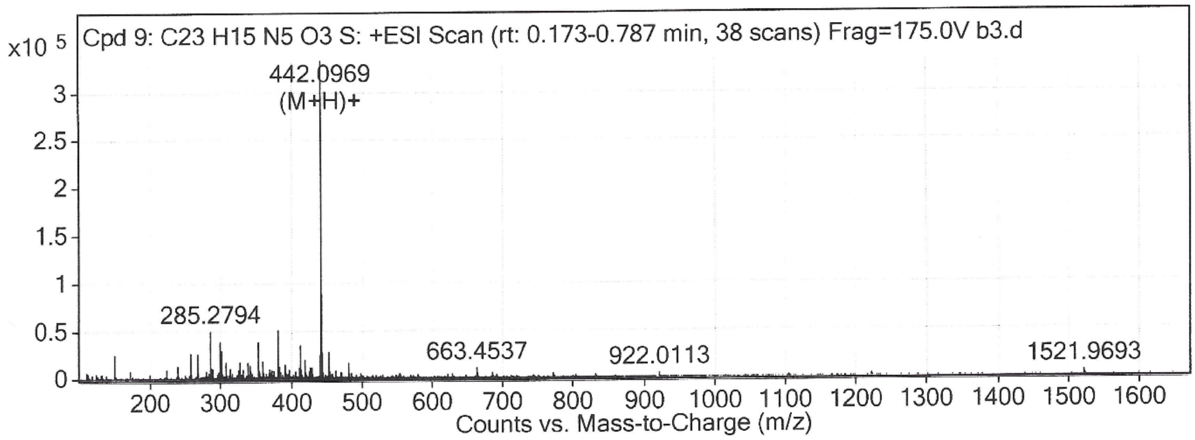

Figure S12. HRMS Spectrum of compound **2**

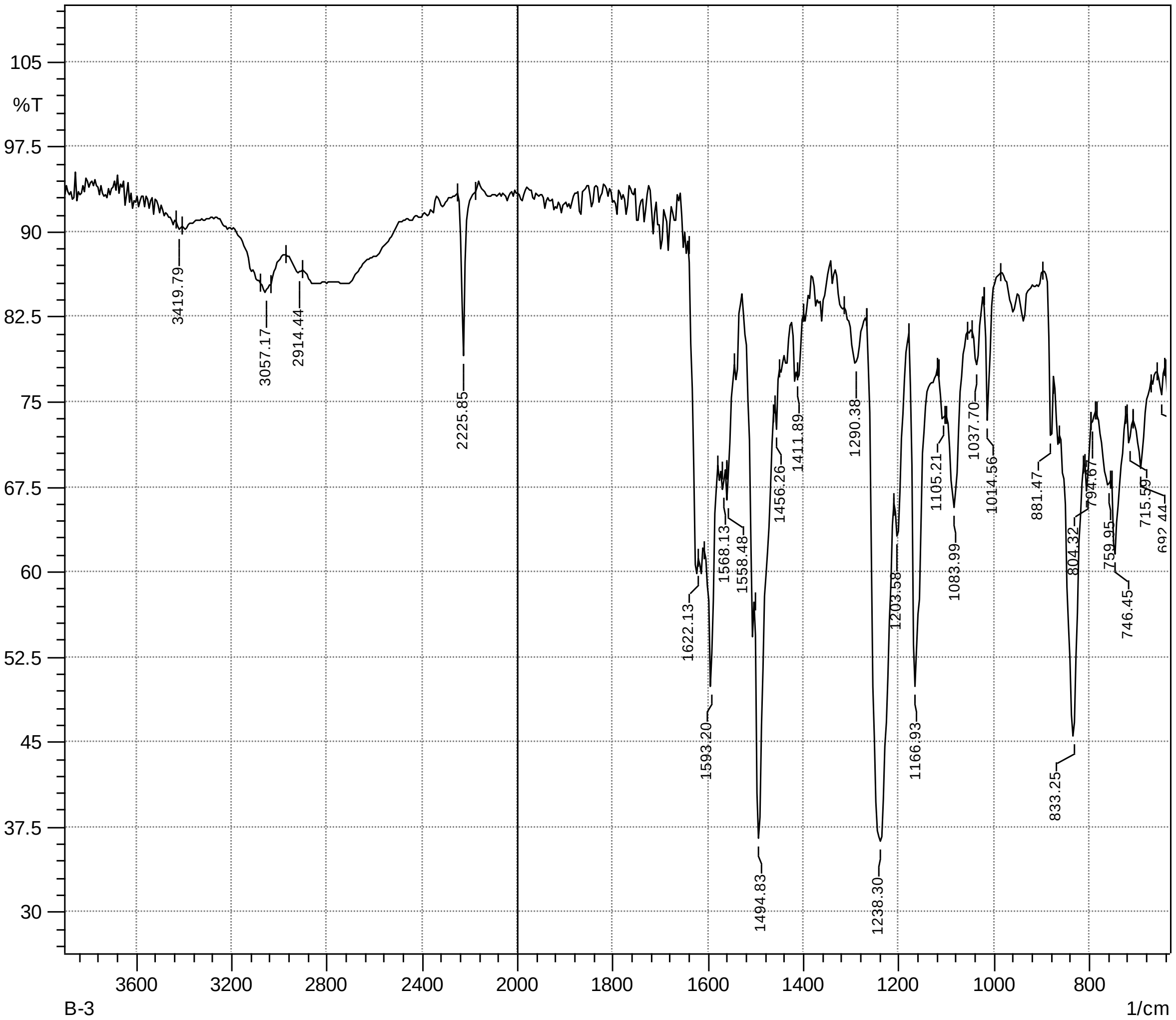

Figure S13. IR Spectrum of compound **3**

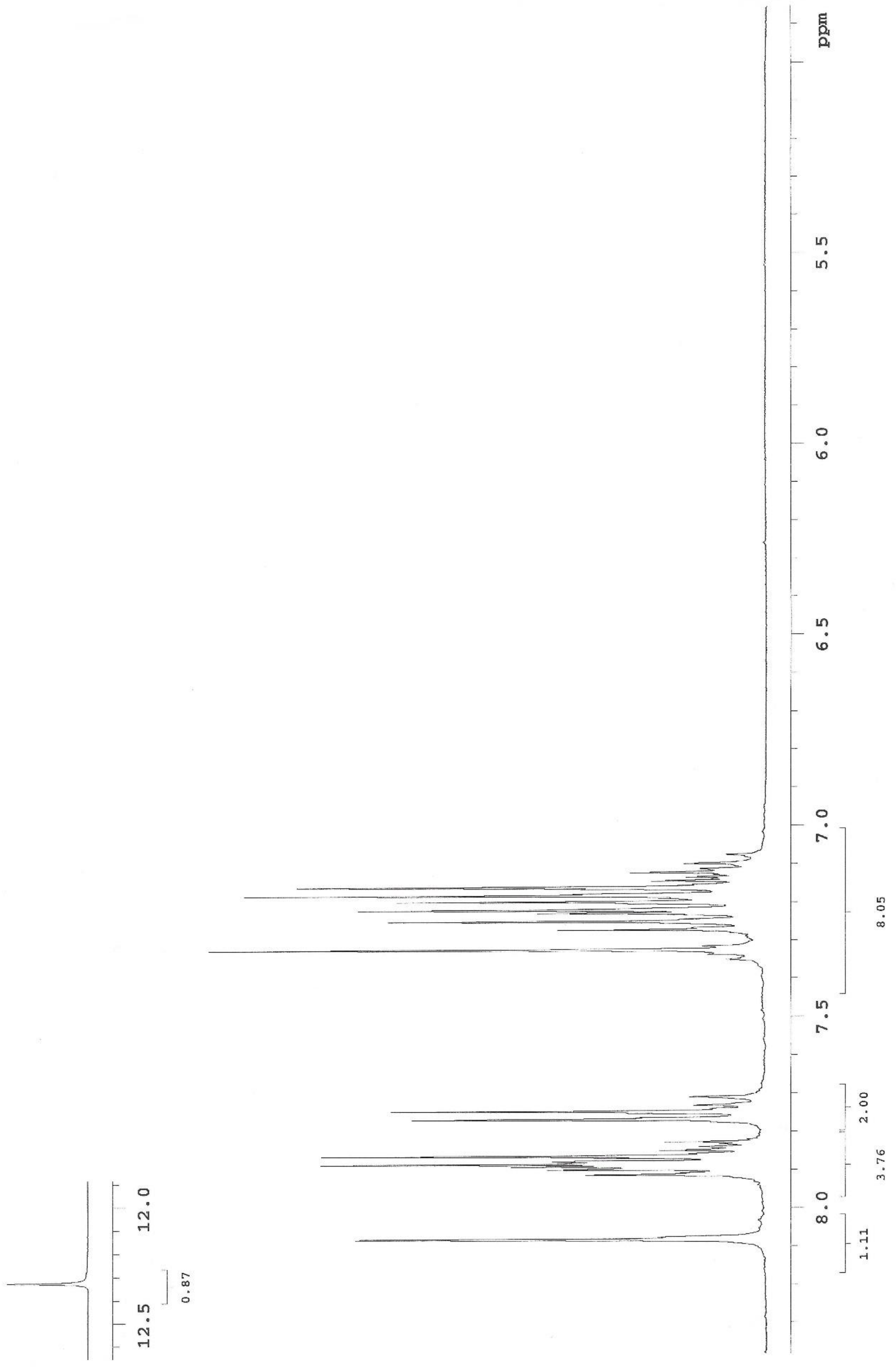

Figure S14. ^{1}H NMR Spectrum of compound **3**

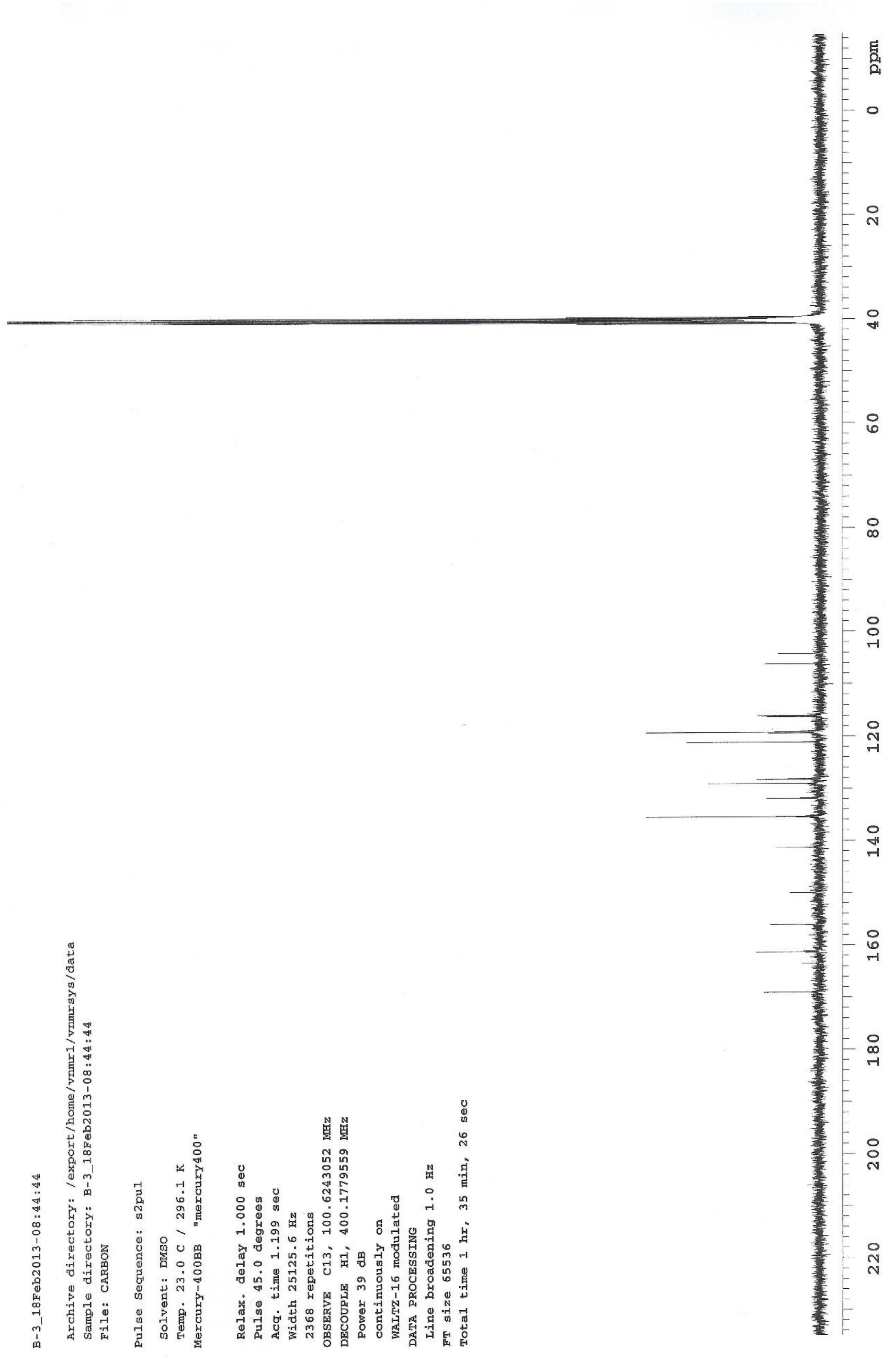

Figure S15. ^{13}C NMR Spectrum of compound **3**

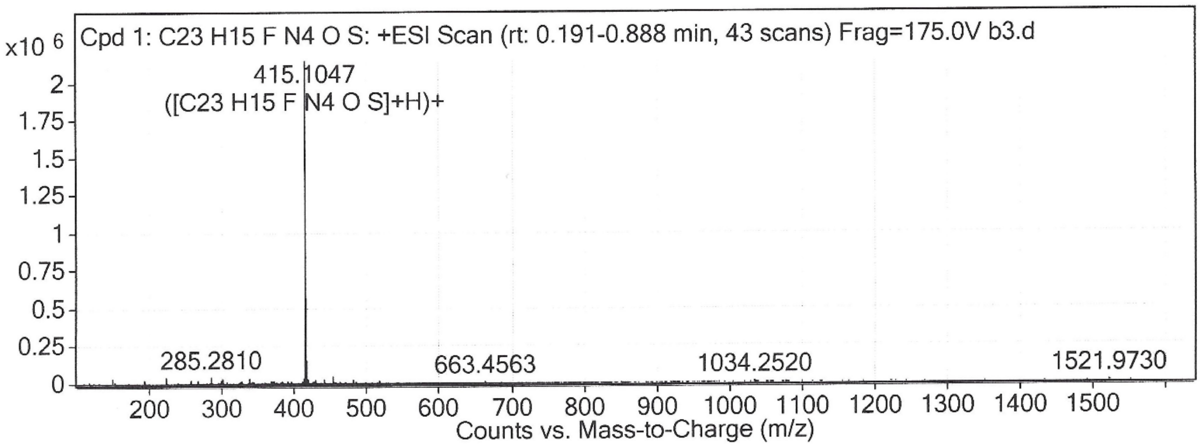

Figure S16. HRMS Spectrum of compound **3**

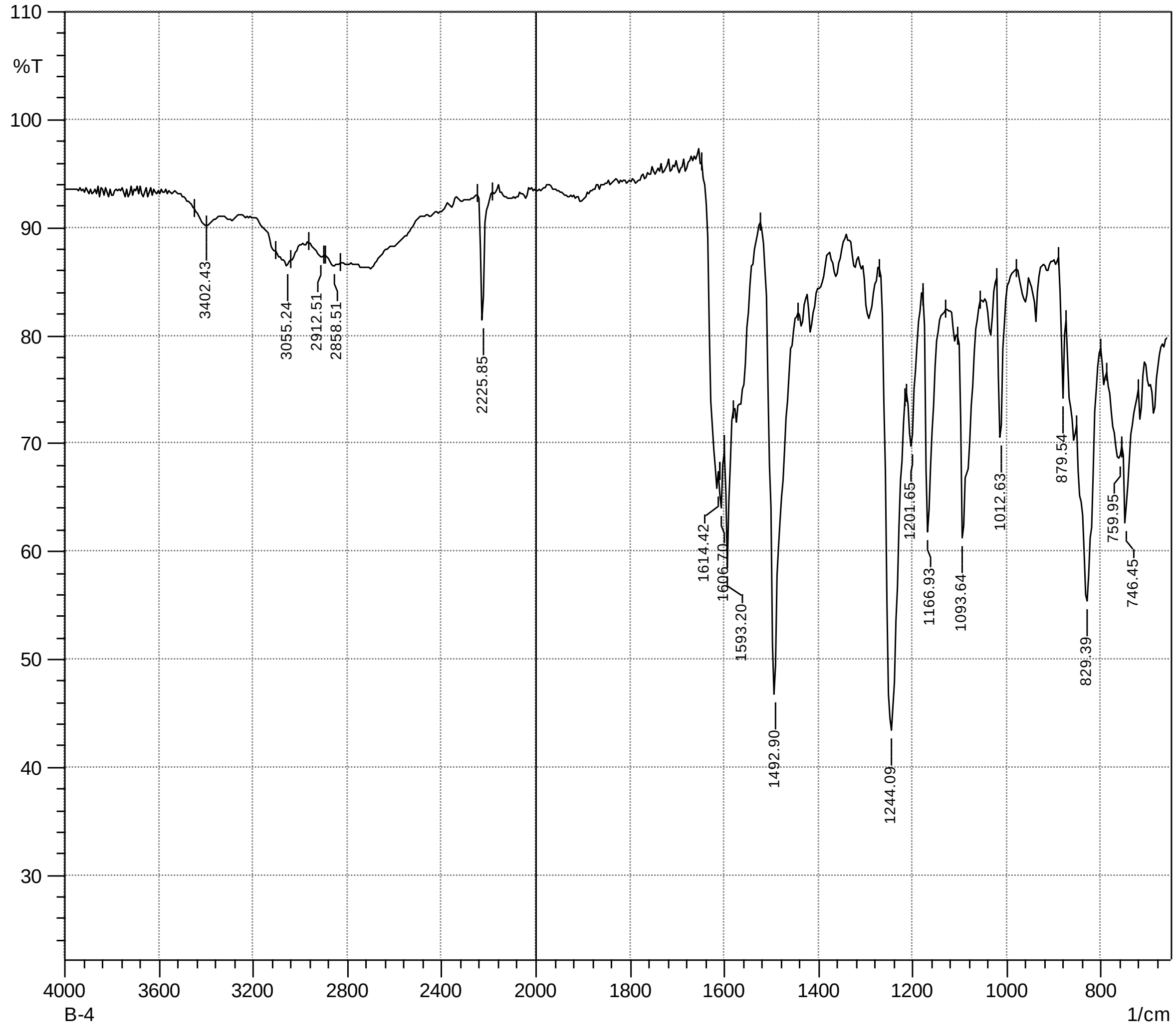

Figure S17. IR Spectrum of compound **4**

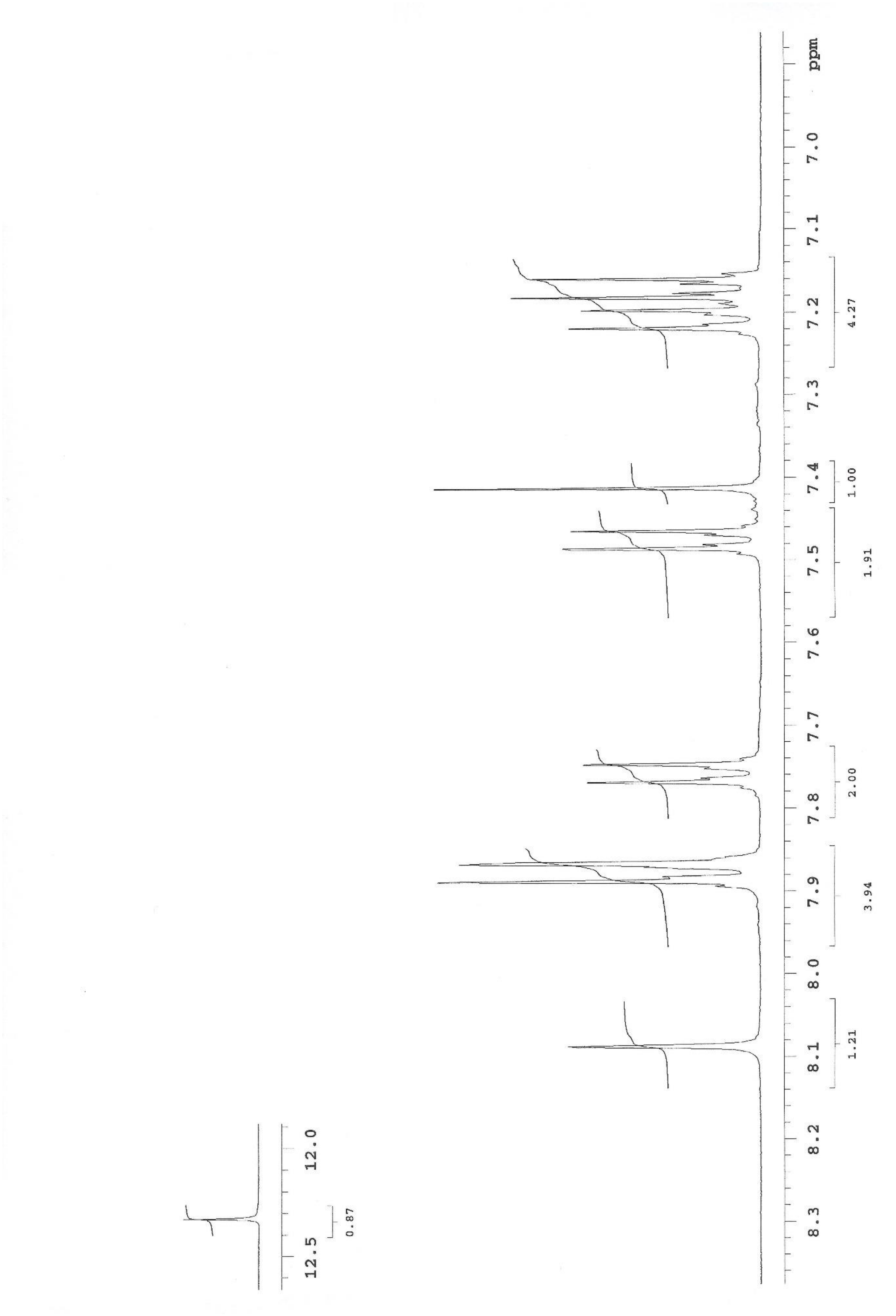

Figure S18. ^{1}H NMR Spectrum of compound **4**

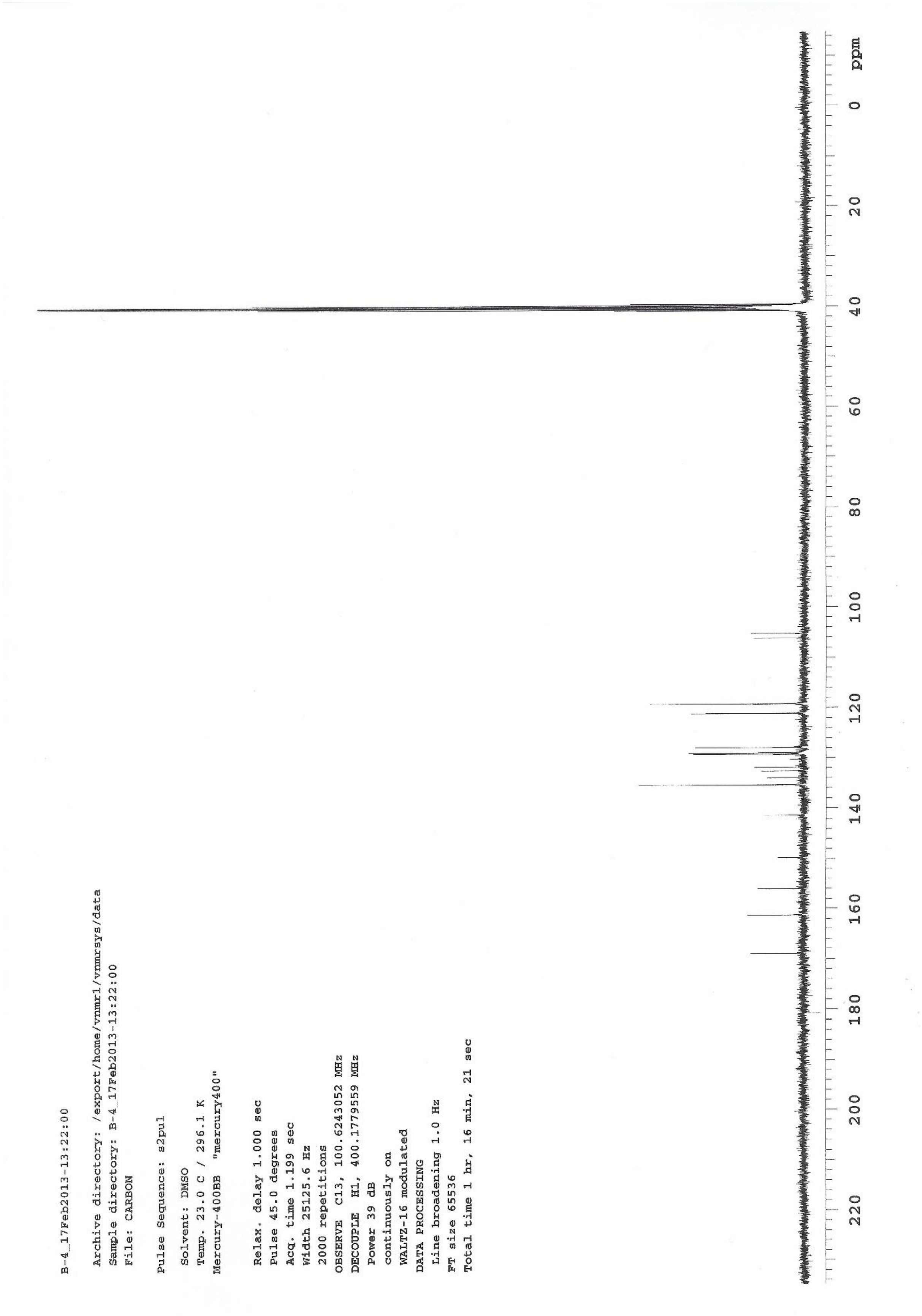

Figure S19. ^{13}C NMR Spectrum of compound **4**

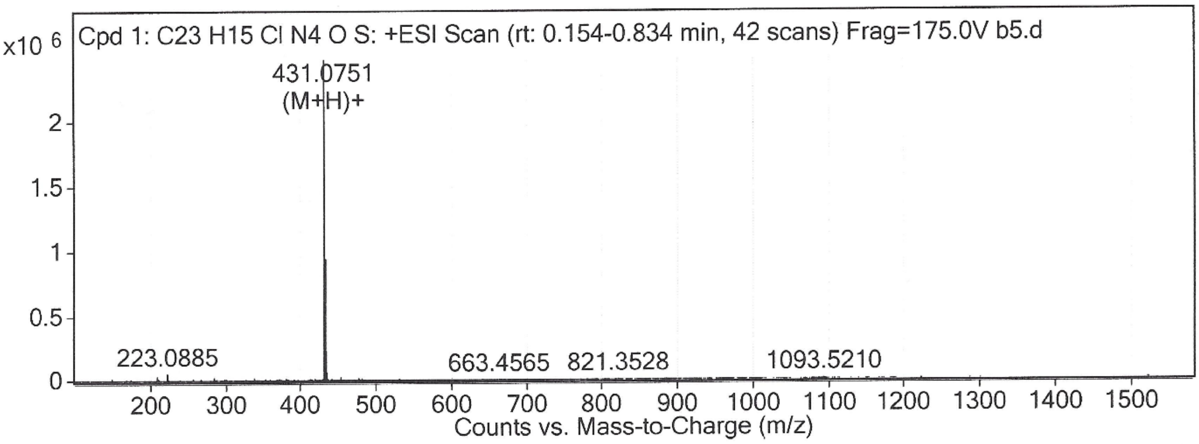

Figure S20. HRMS Spectrum of compound **4**

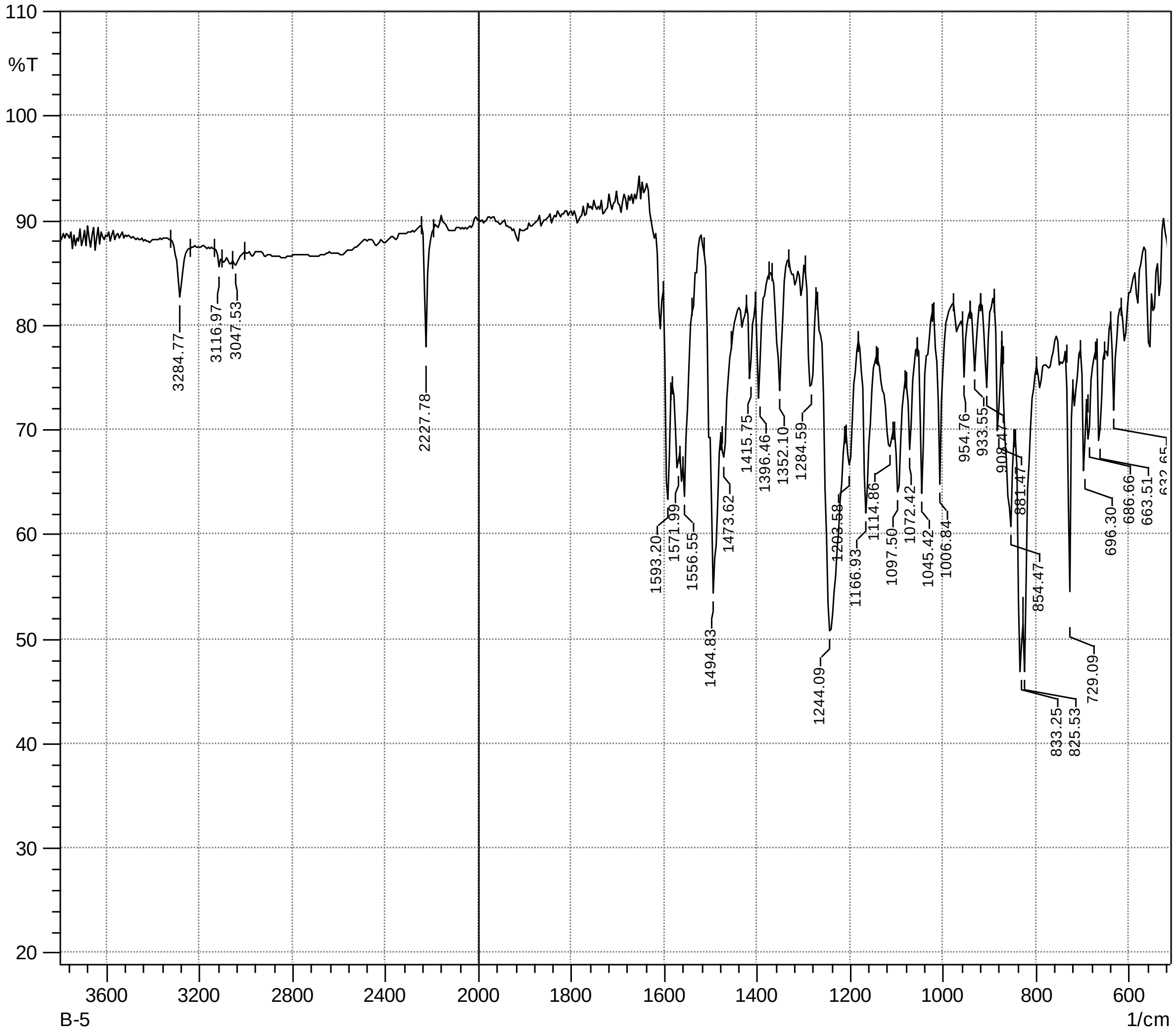

Figure S21. IR Spectrum of compound **5**

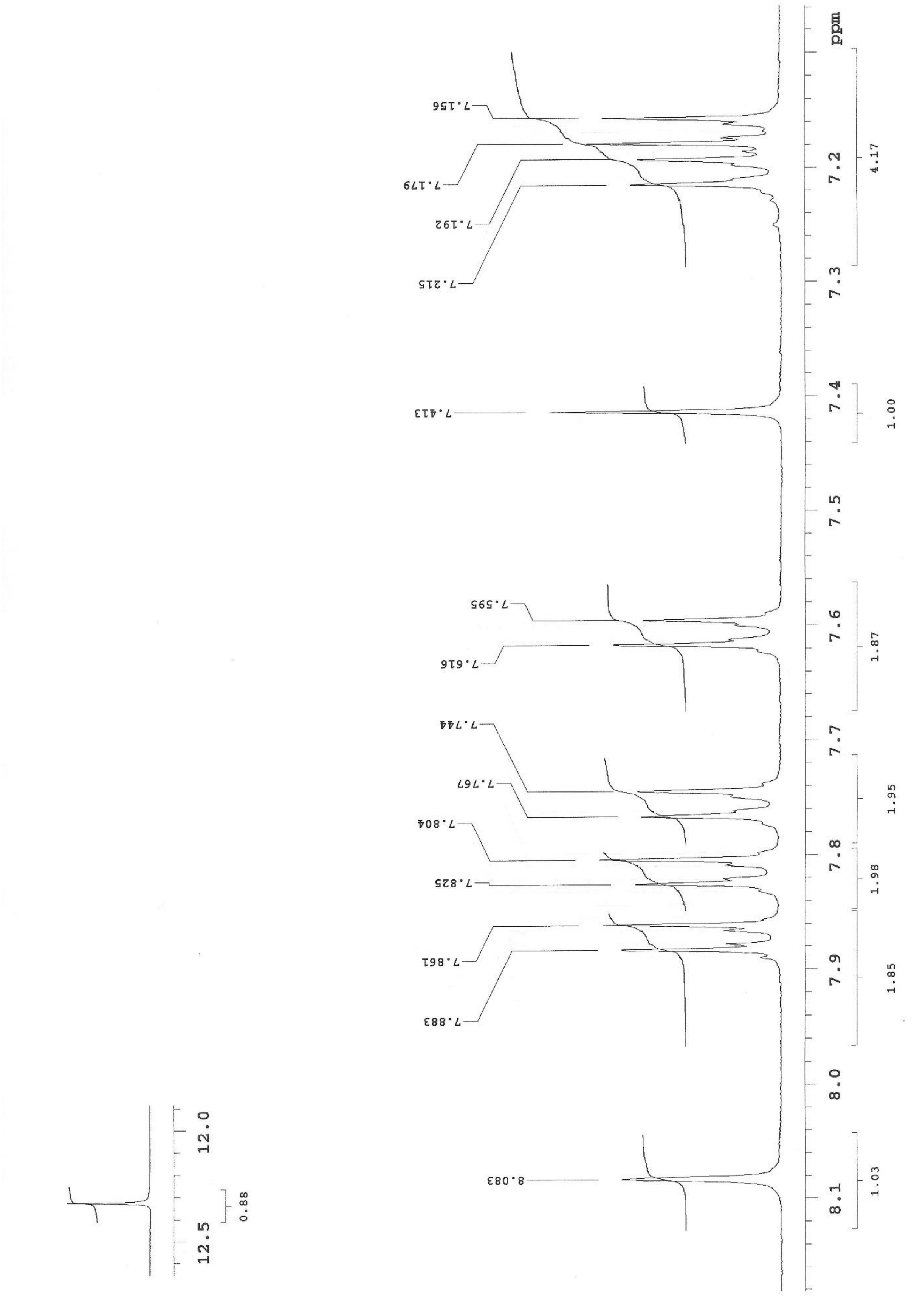

Figure S22. ^{1}H NMR Spectrum of compound **5**

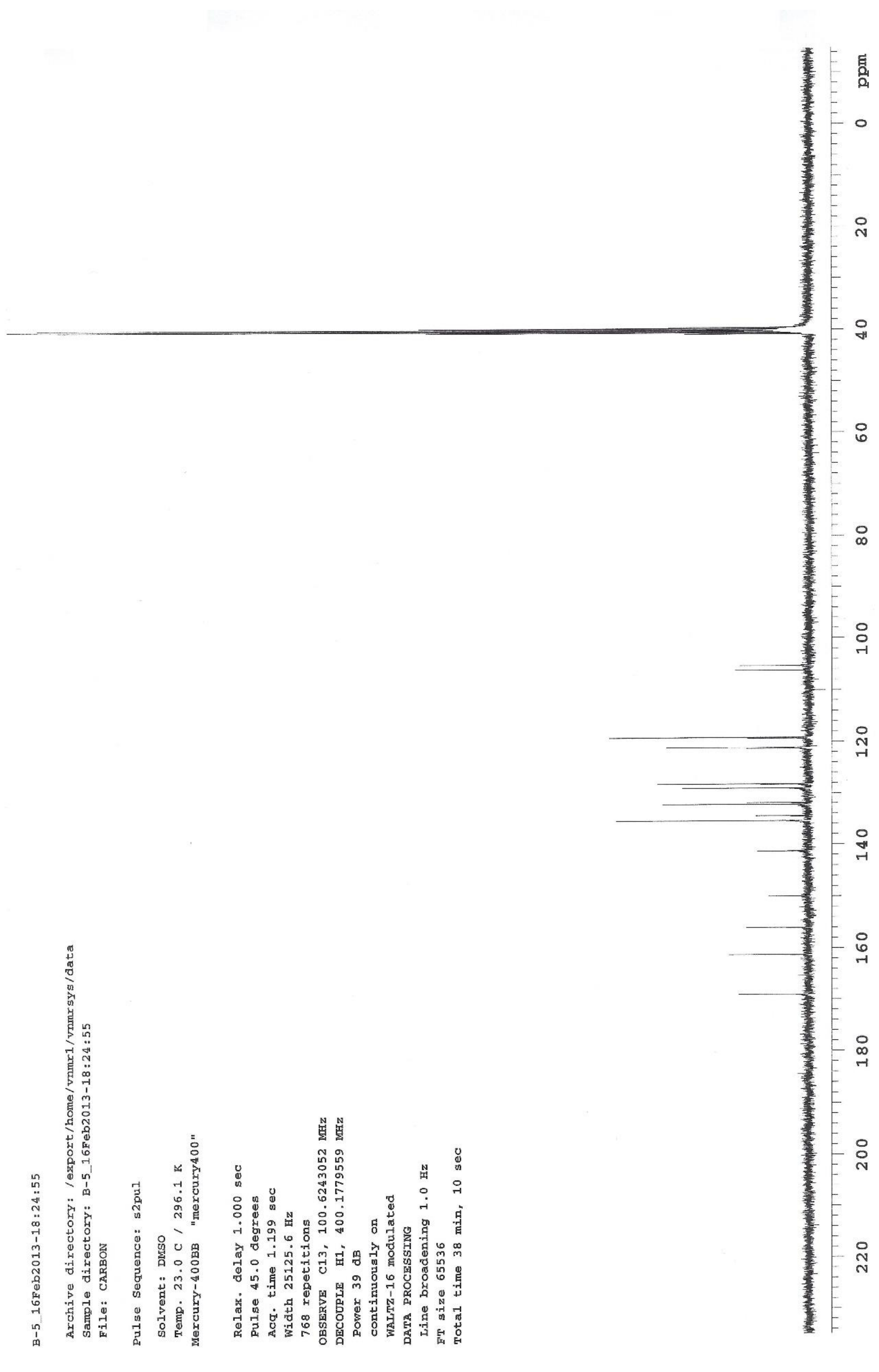

Figure S23. ^{13}C NMR Spectrum of compound **5**

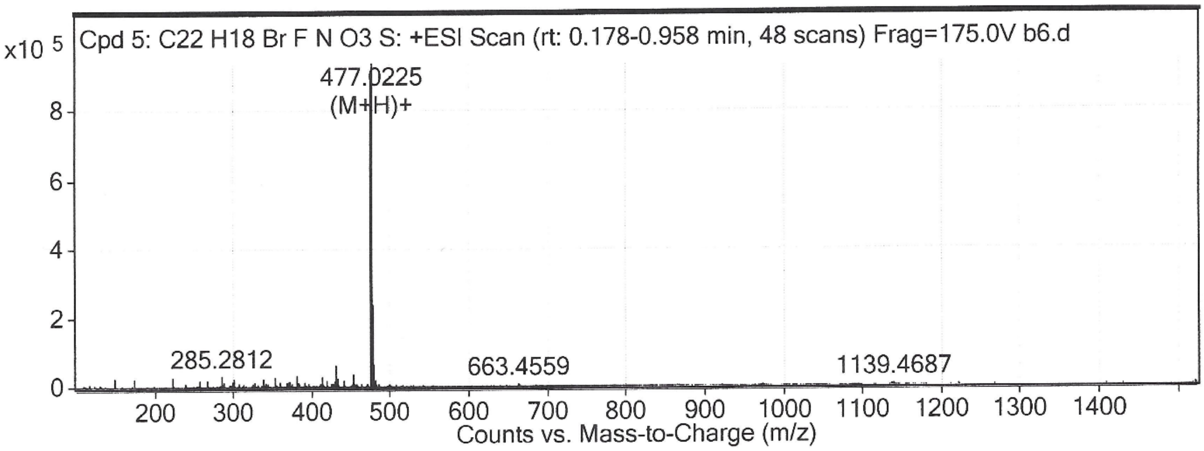

Figure S24. HRMS Spectrum of compound **5**

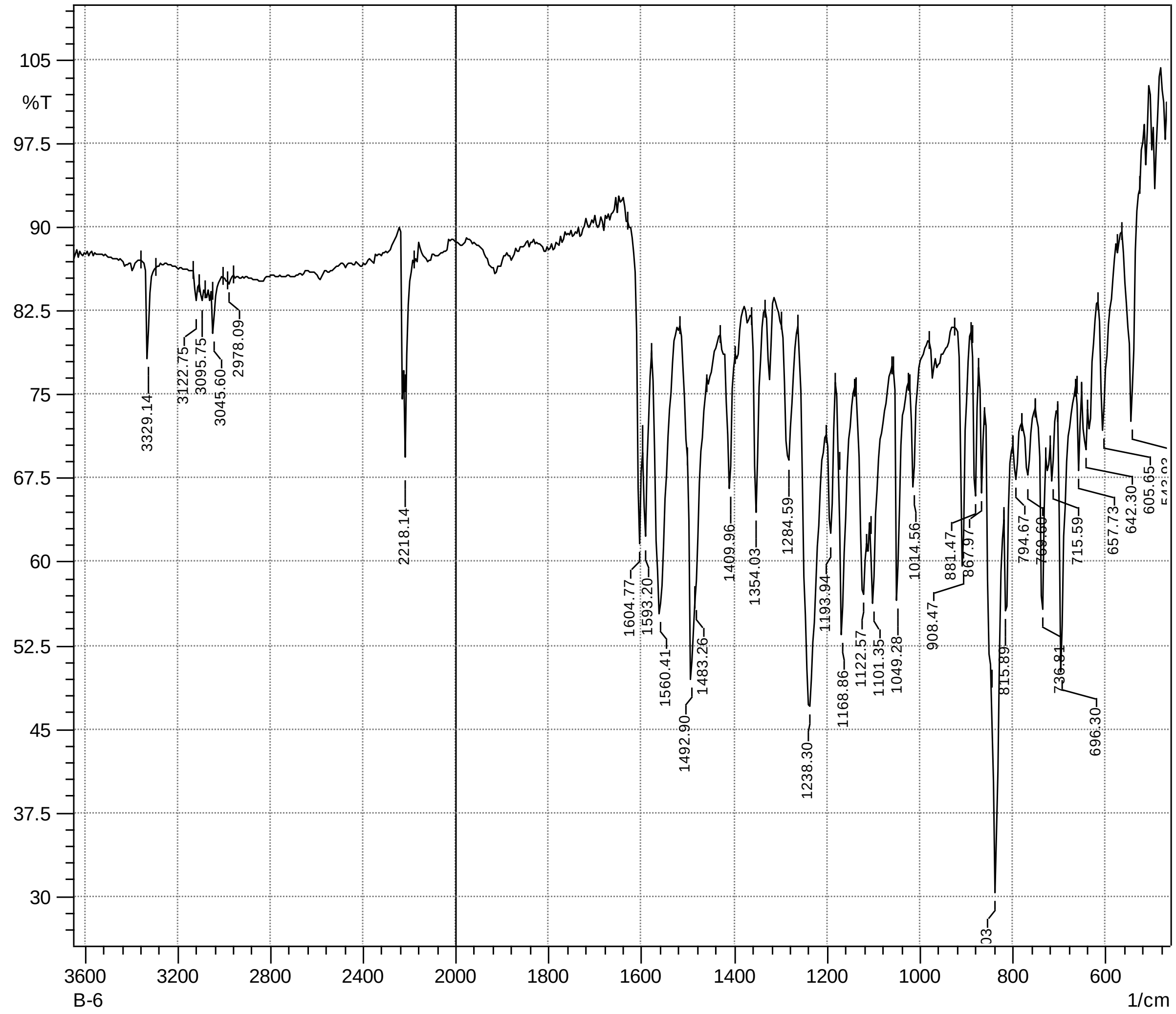

Figure S25. IR Spectrum of compound **6**

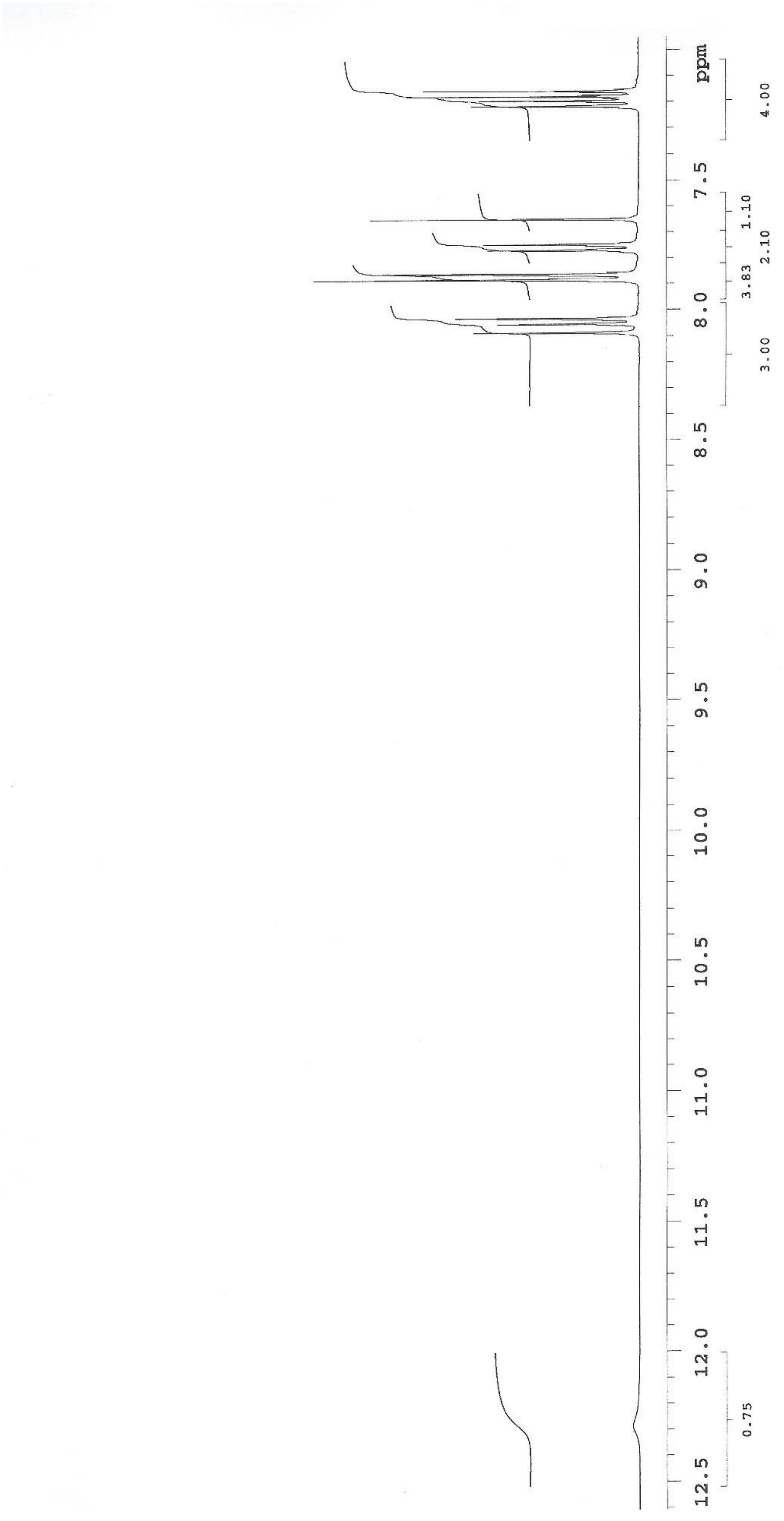

Figure S26. ^{1}H NMR Spectrum of compound **6**

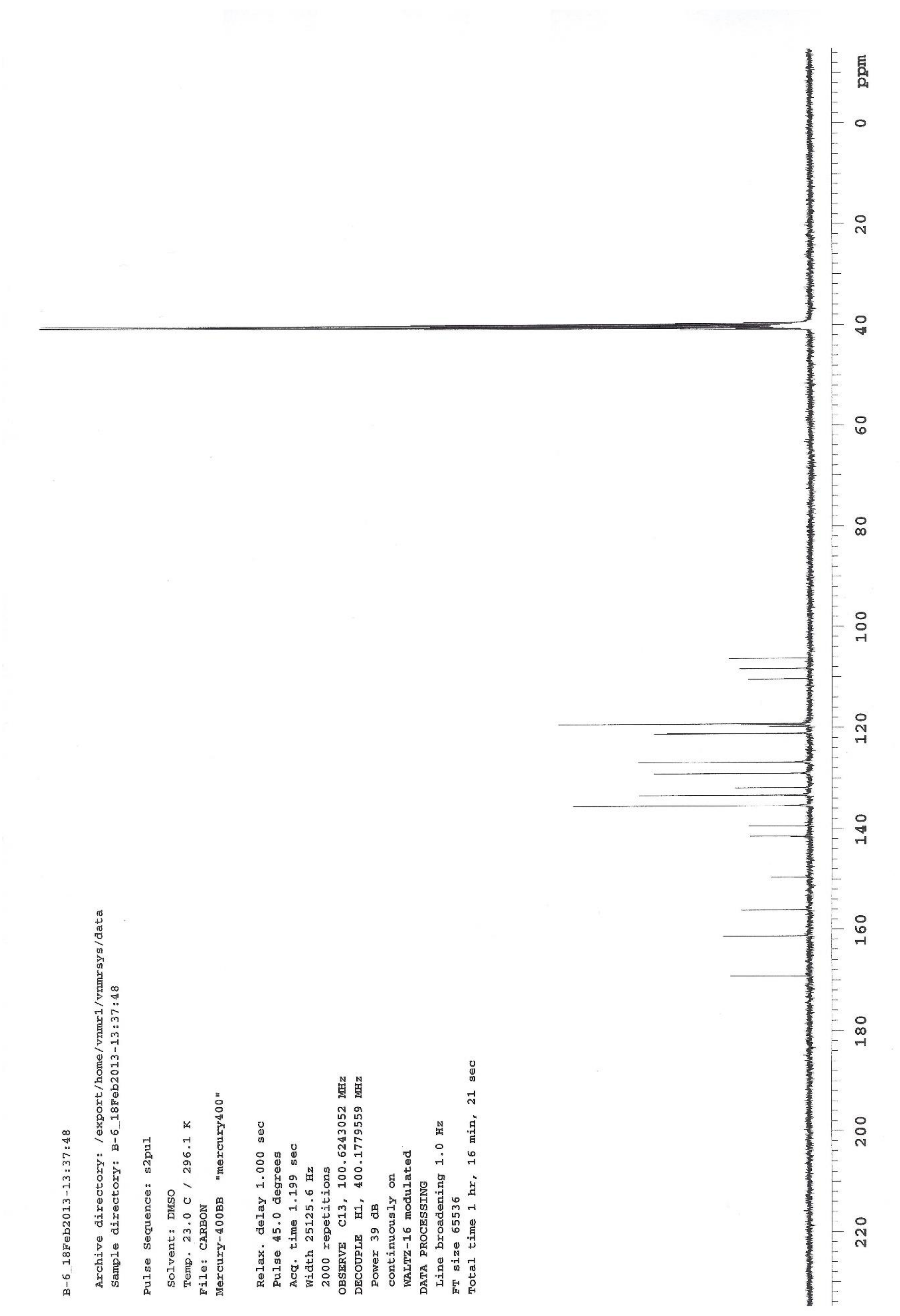

Figure S27. ^{13}C NMR Spectrum of compound **6**

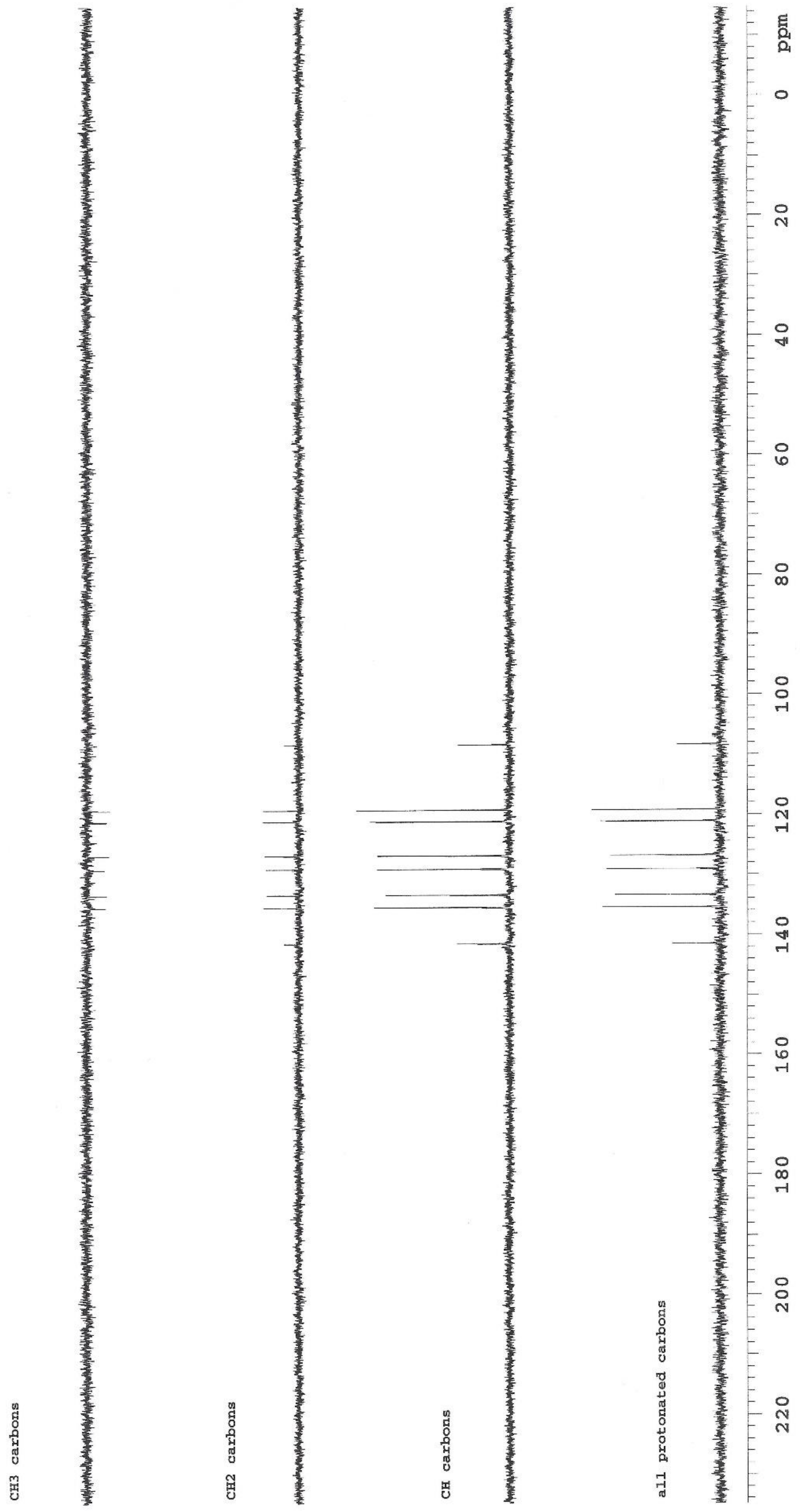

Figure S28. ^{13}C NMR (DEPT) Spectrum of compound **6**

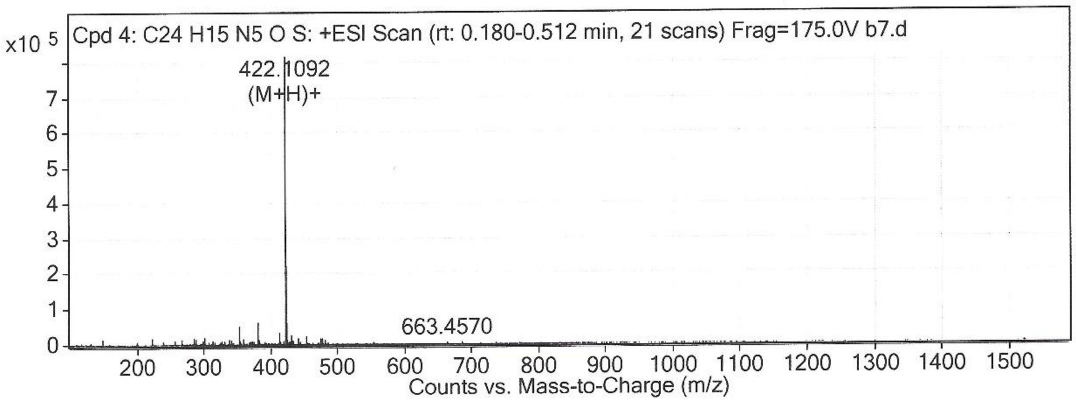

Figure S29. HRMS Spectrum of compound **6**

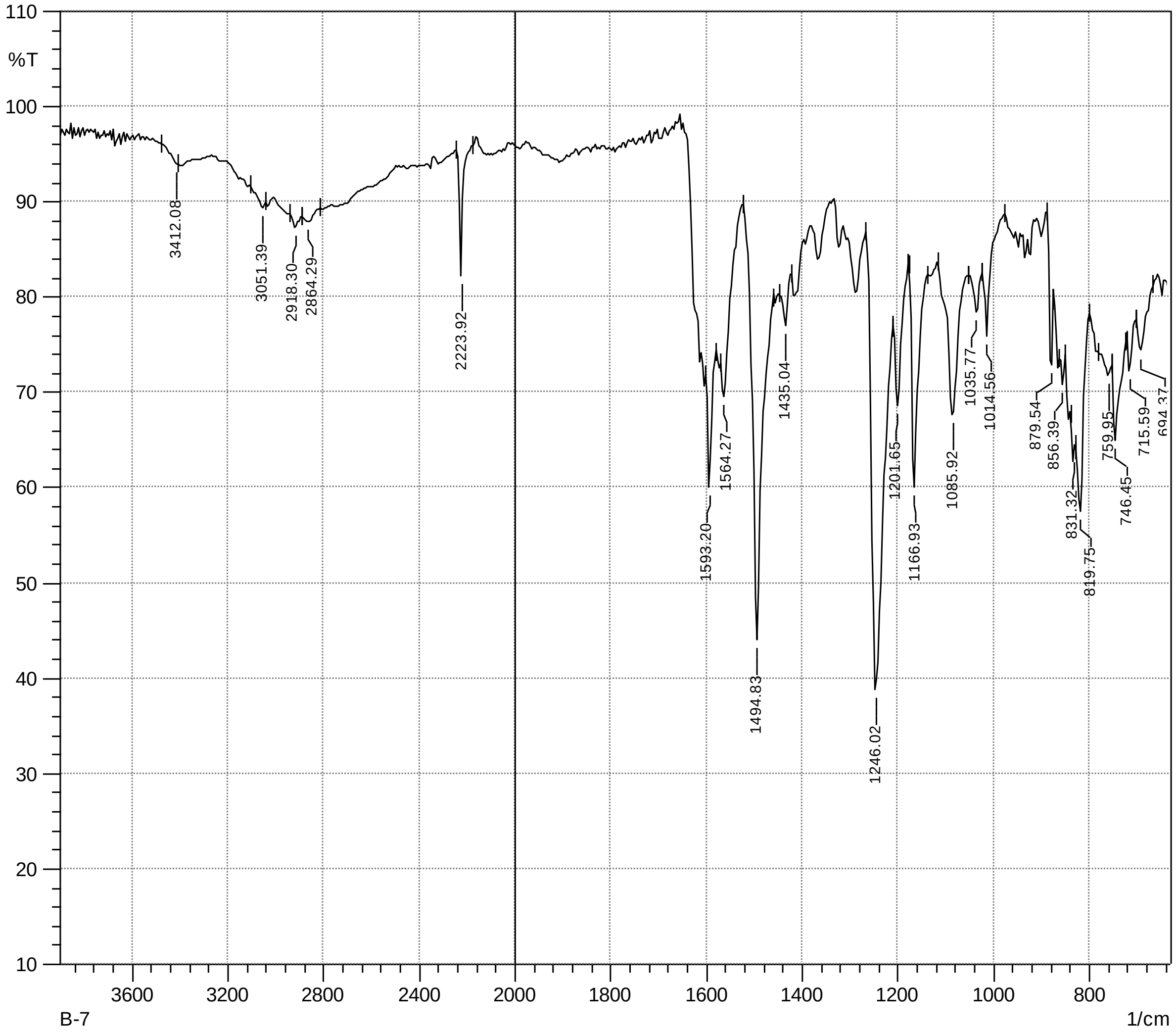

Figure S30. IR Spectrum of compound **7**

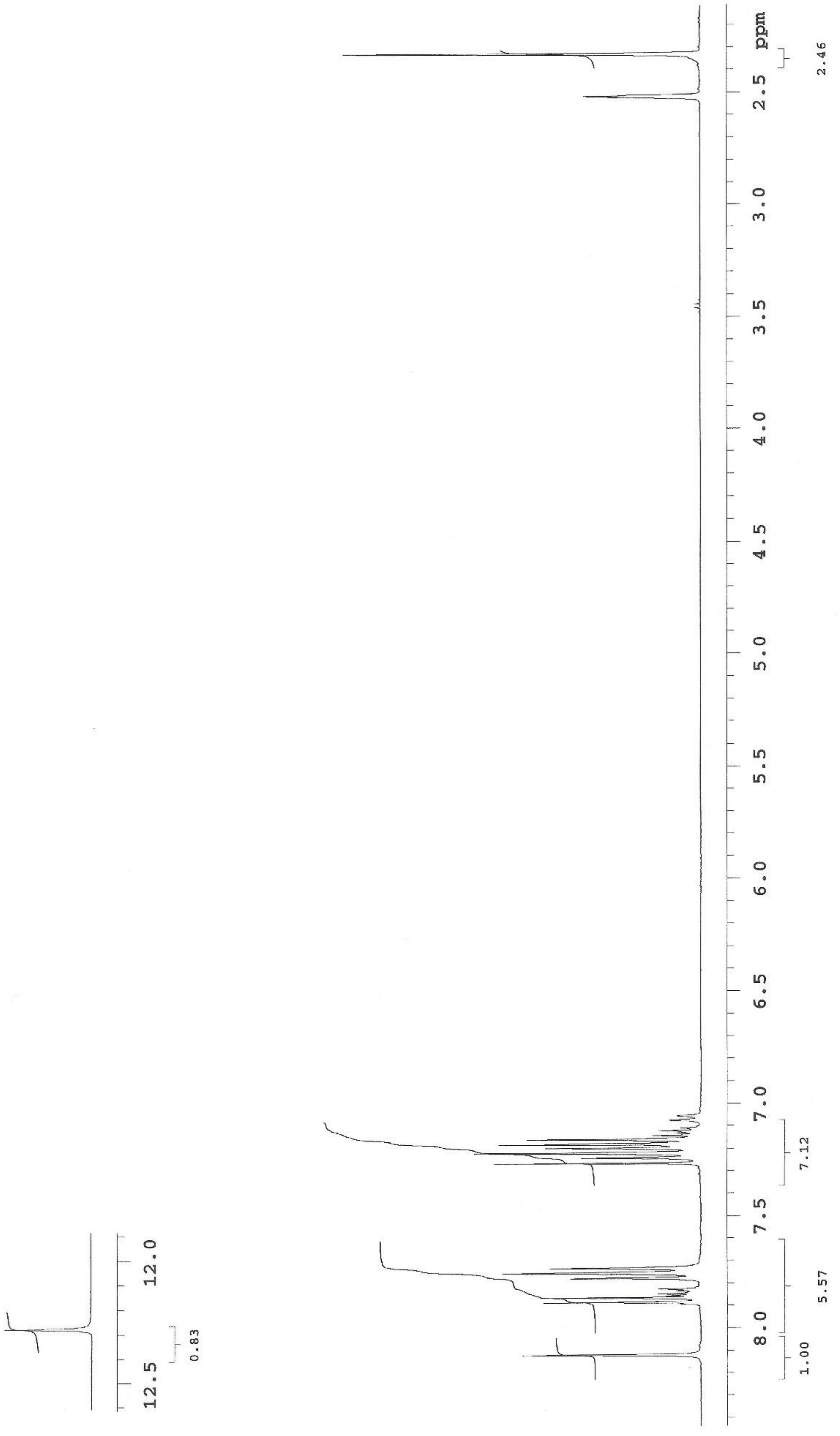

Figure S31. ^{1}H NMR Spectrum of compound **7**

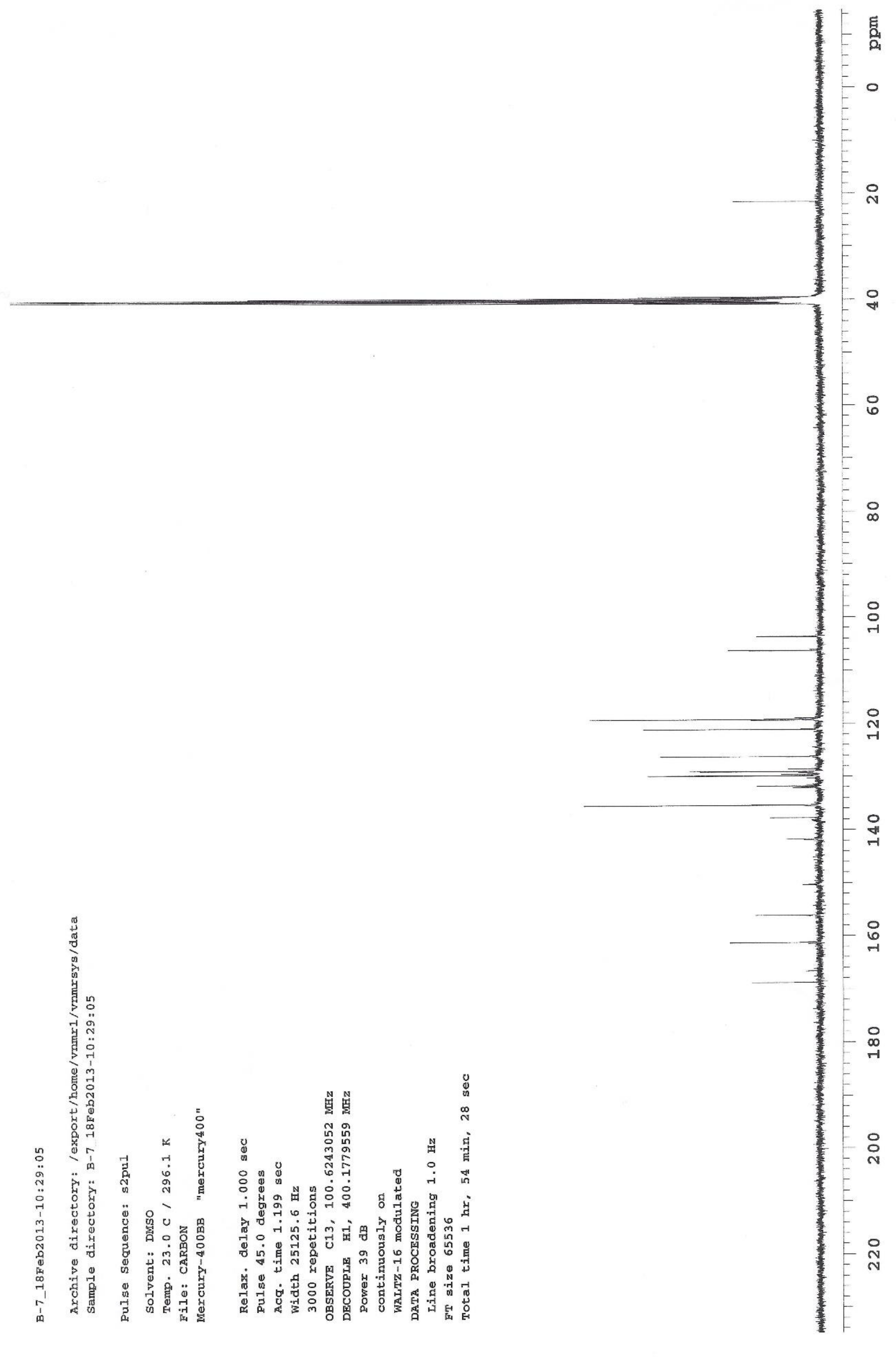

Figure S32. ^{13}C NMR Spectrum of compound **7**

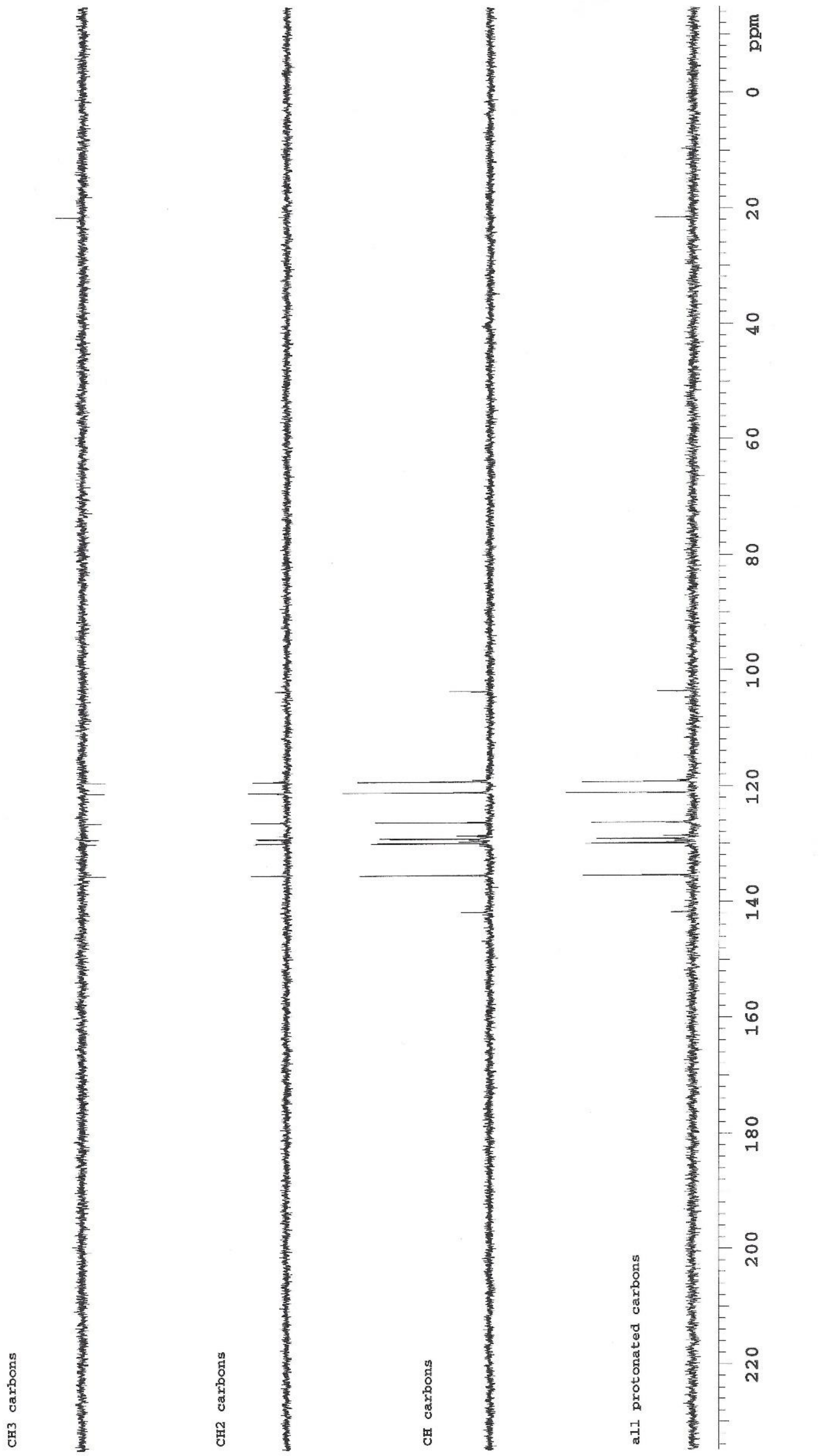

Figure S33. ^{13}C NMR (DEPT) Spectrum of compound **7**

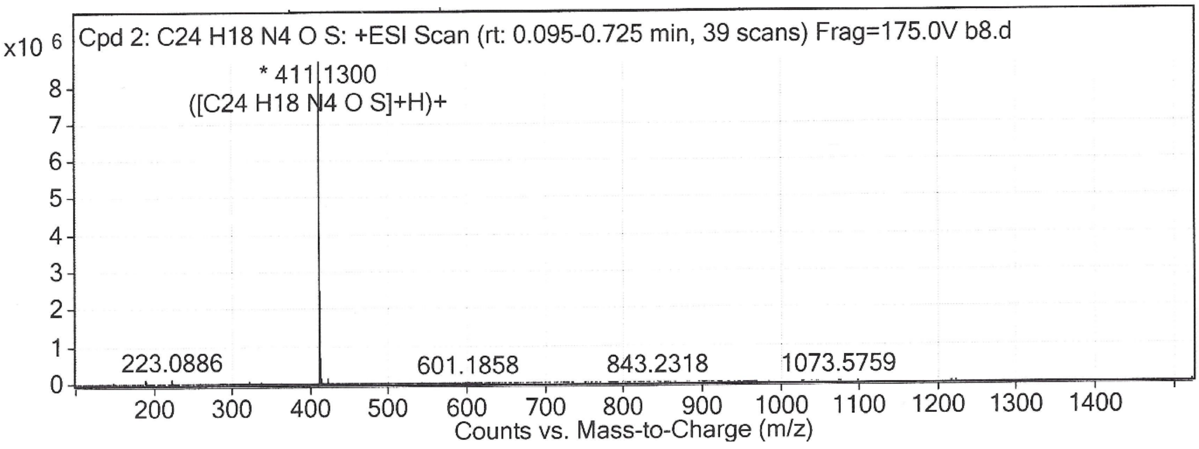

Figure S34. HRMS Spectrum of compound **7**

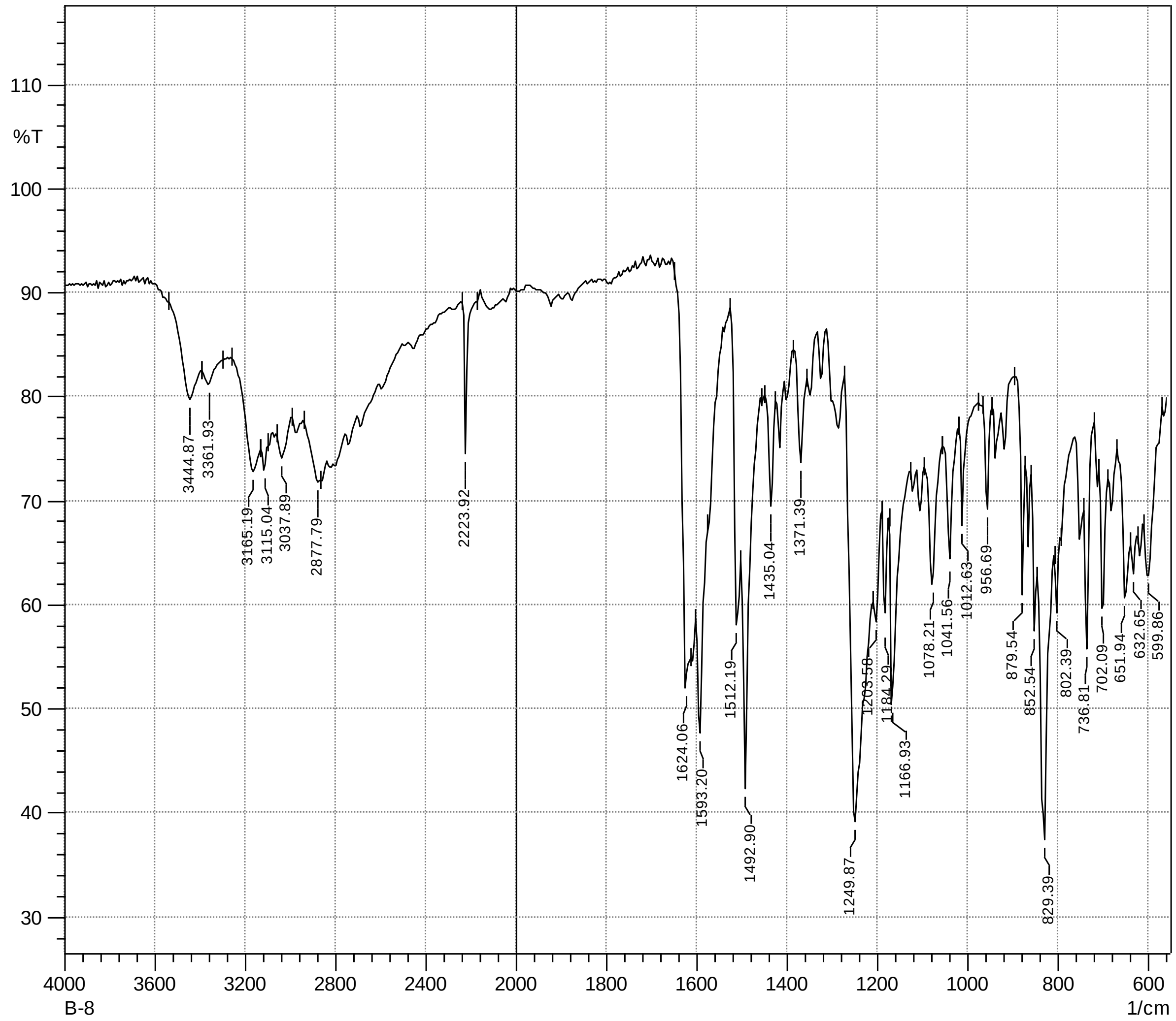

Figure S35. IR Spectrum of compound **8**

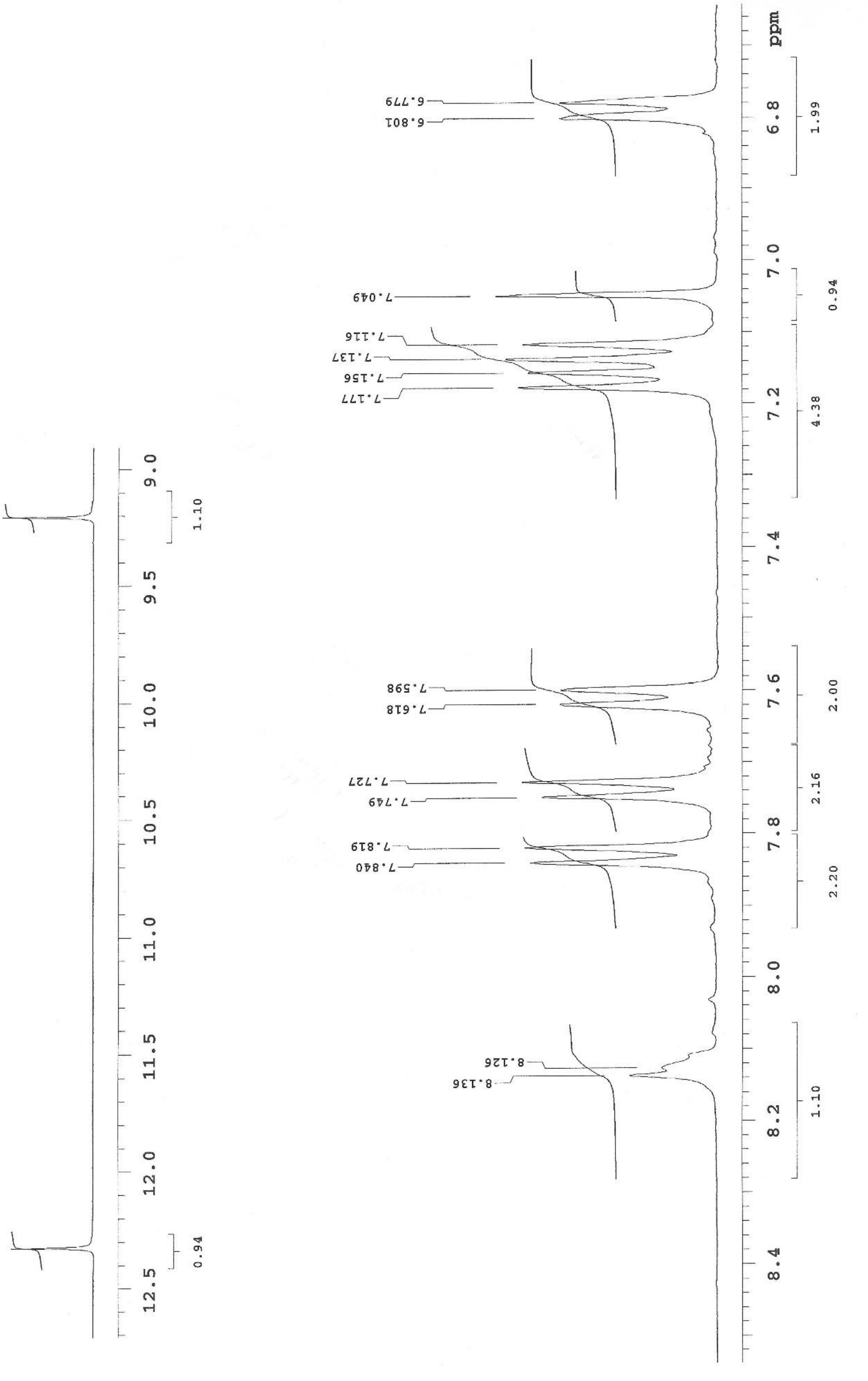

Figure S36. ^{1}H NMR Spectrum of compound **8**

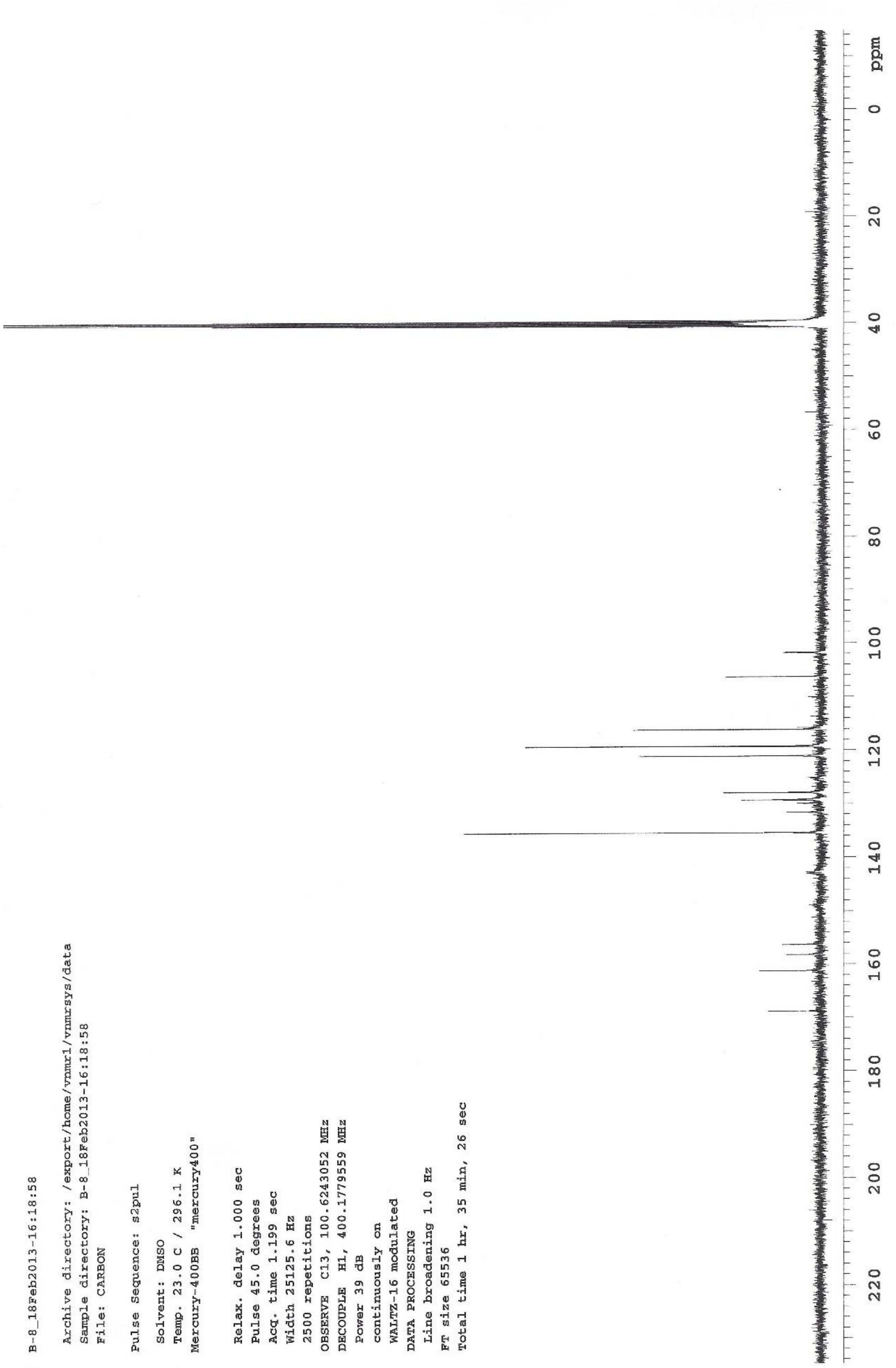

Figure S37. ^{13}C NMR Spectrum of compound **8**

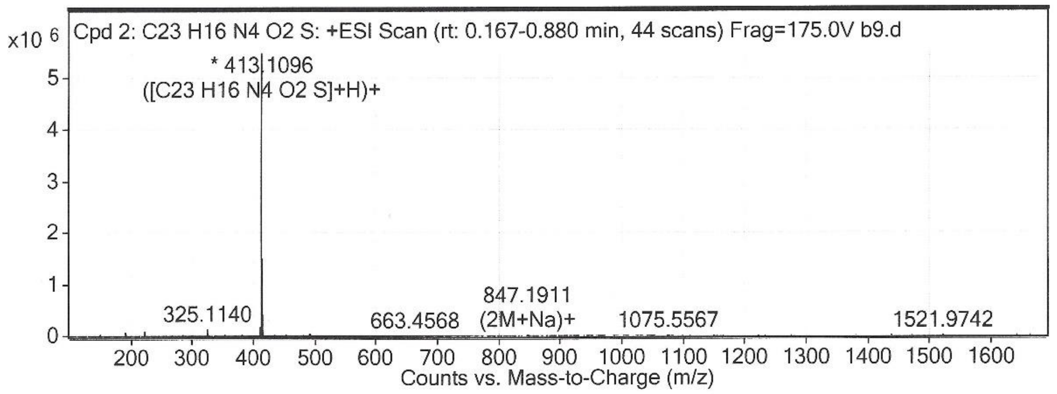

Figure S38. HRMS Spectrum of compound **8**

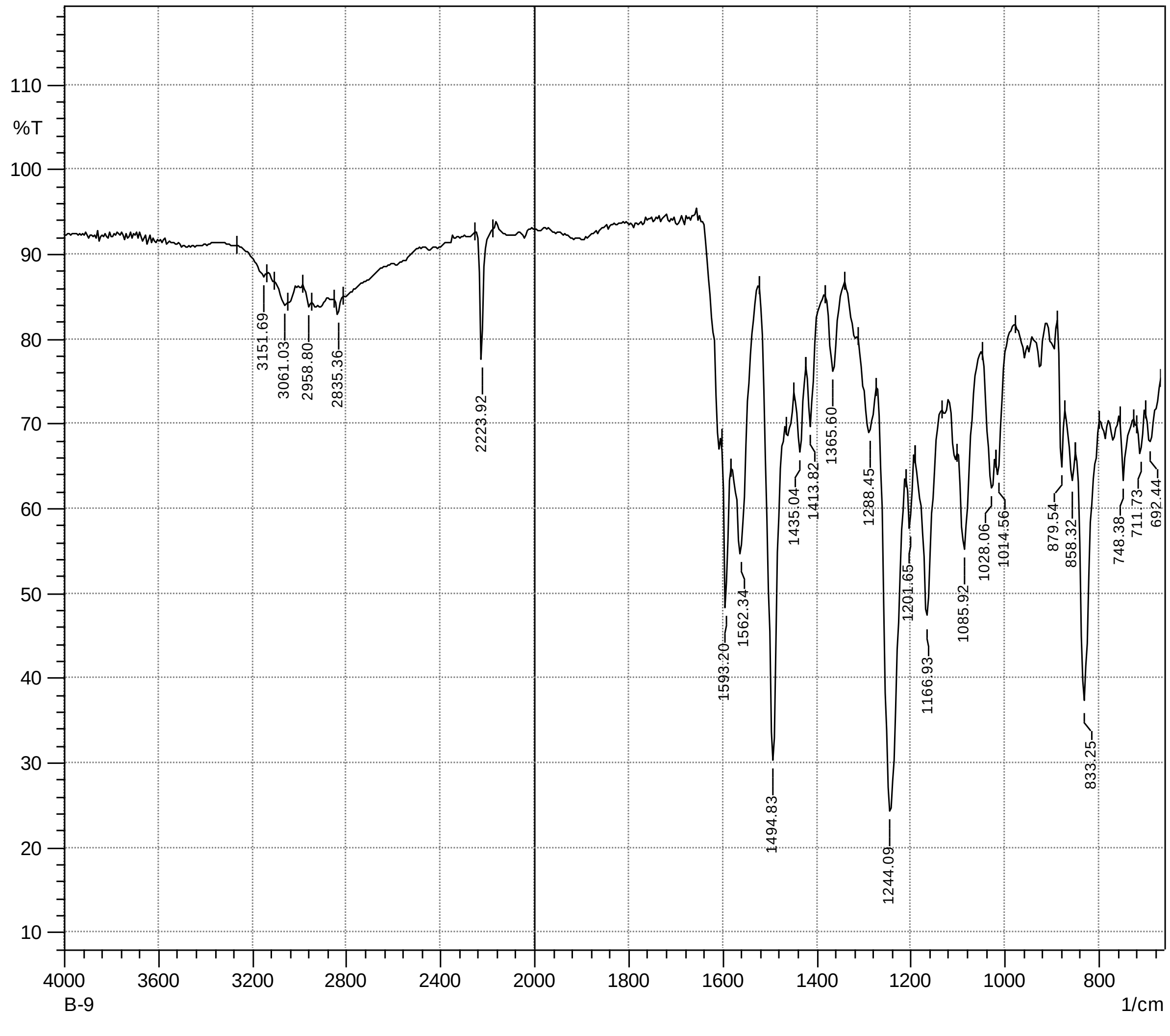

Figure S39. IR Spectrum of compound **9**

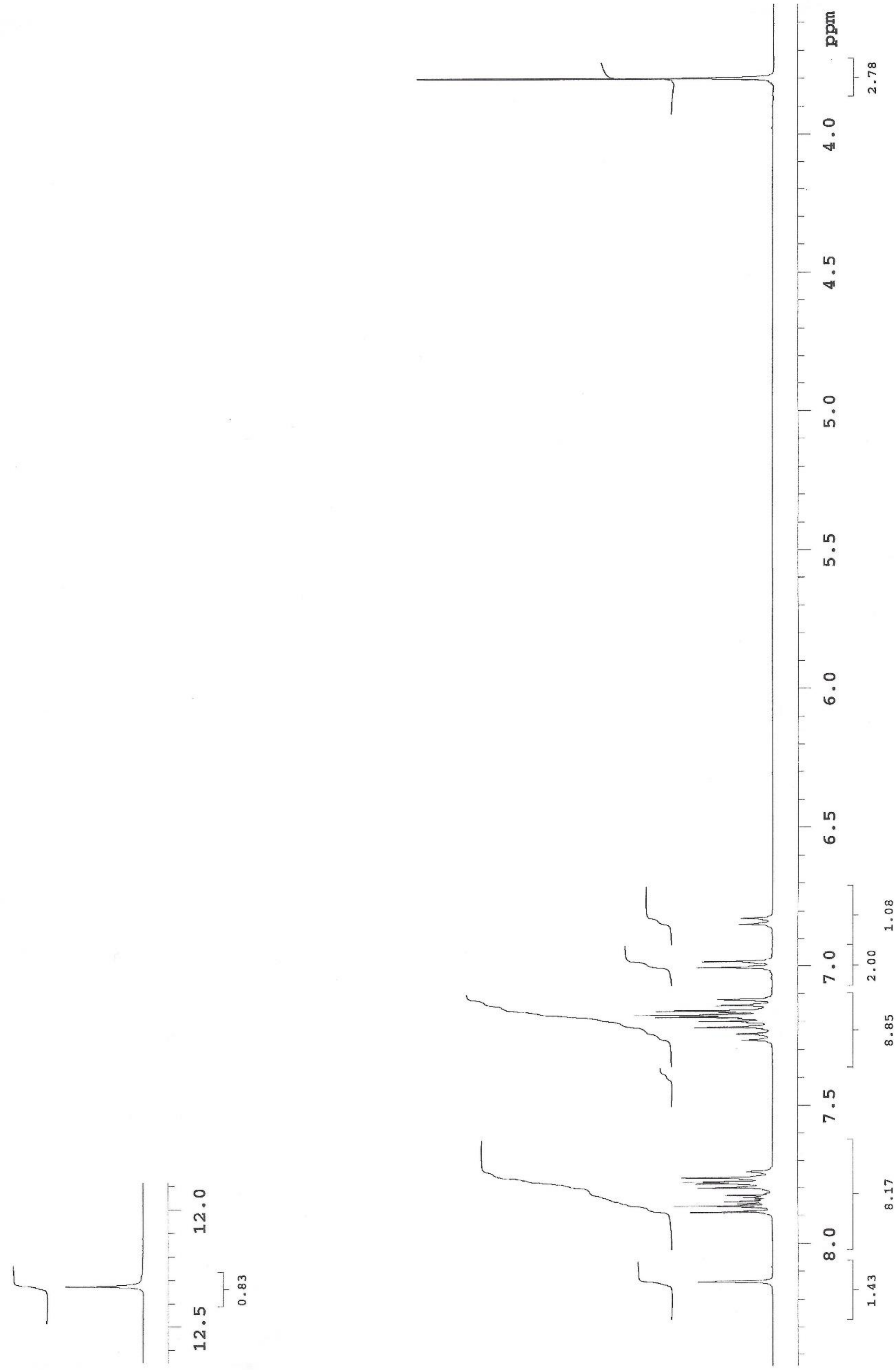

Figure S40. ^{1}H NMR Spectrum of compound **9**

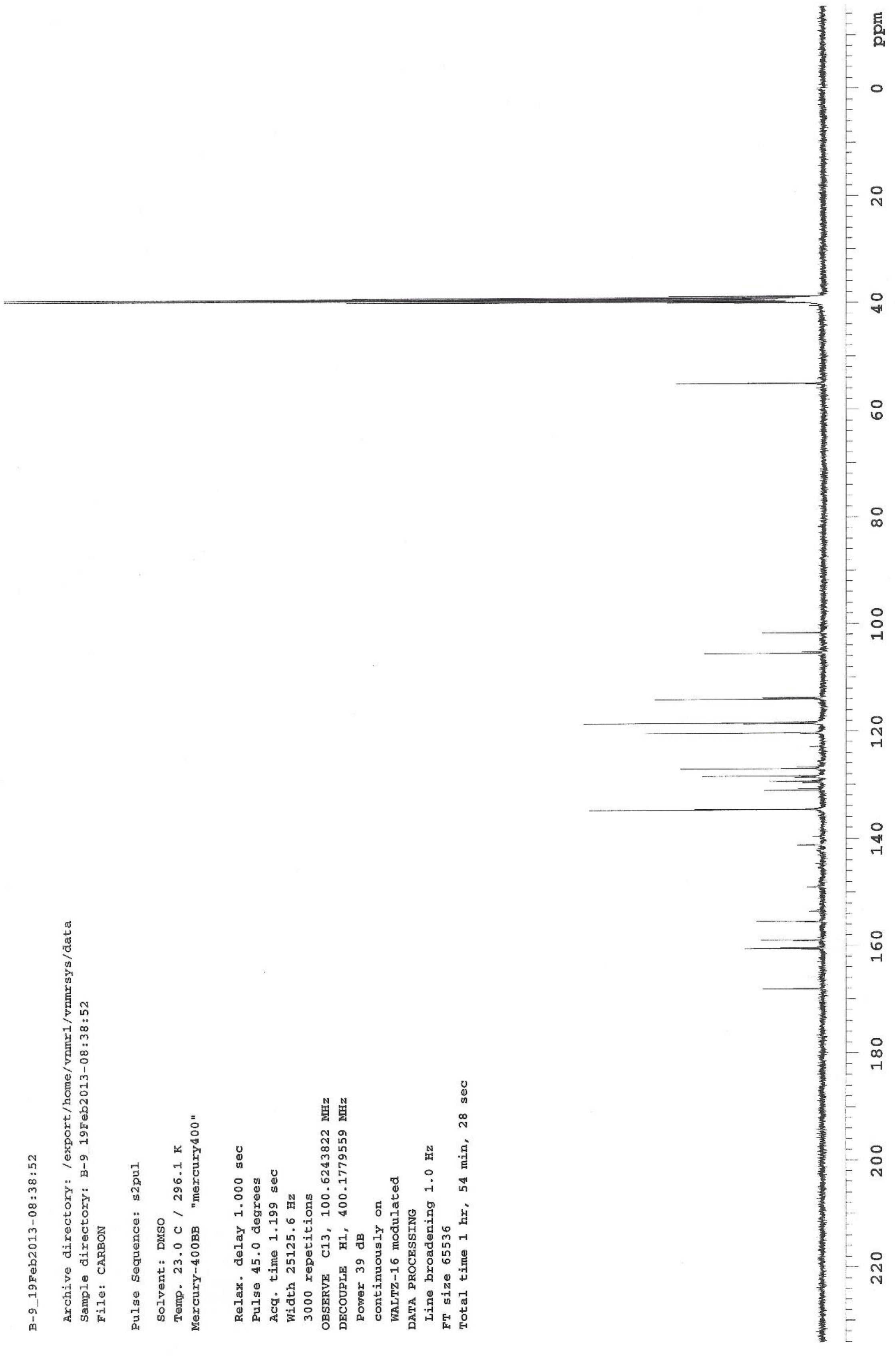

Figure S41. ^{13}C NMR Spectrum of compound **9**

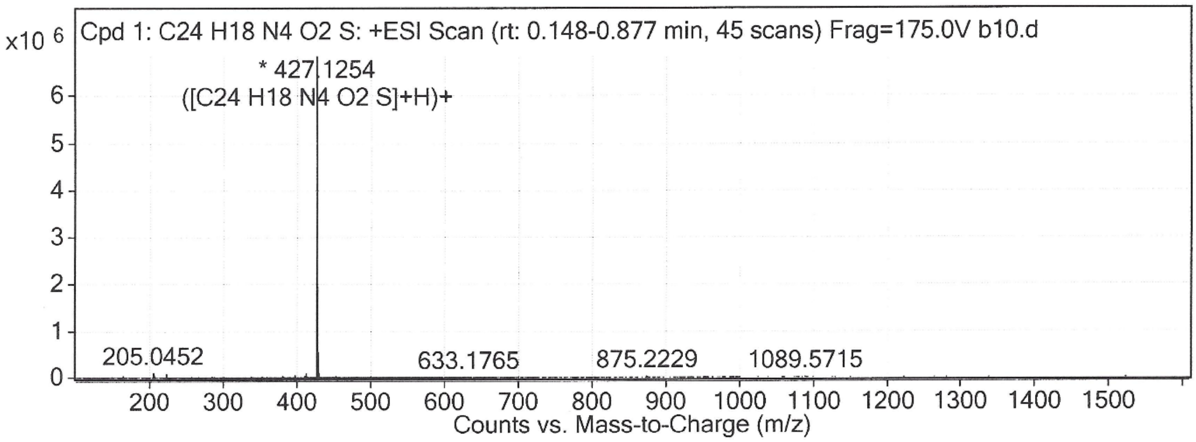

Figure S42. HRMS Spectrum of compound **9**

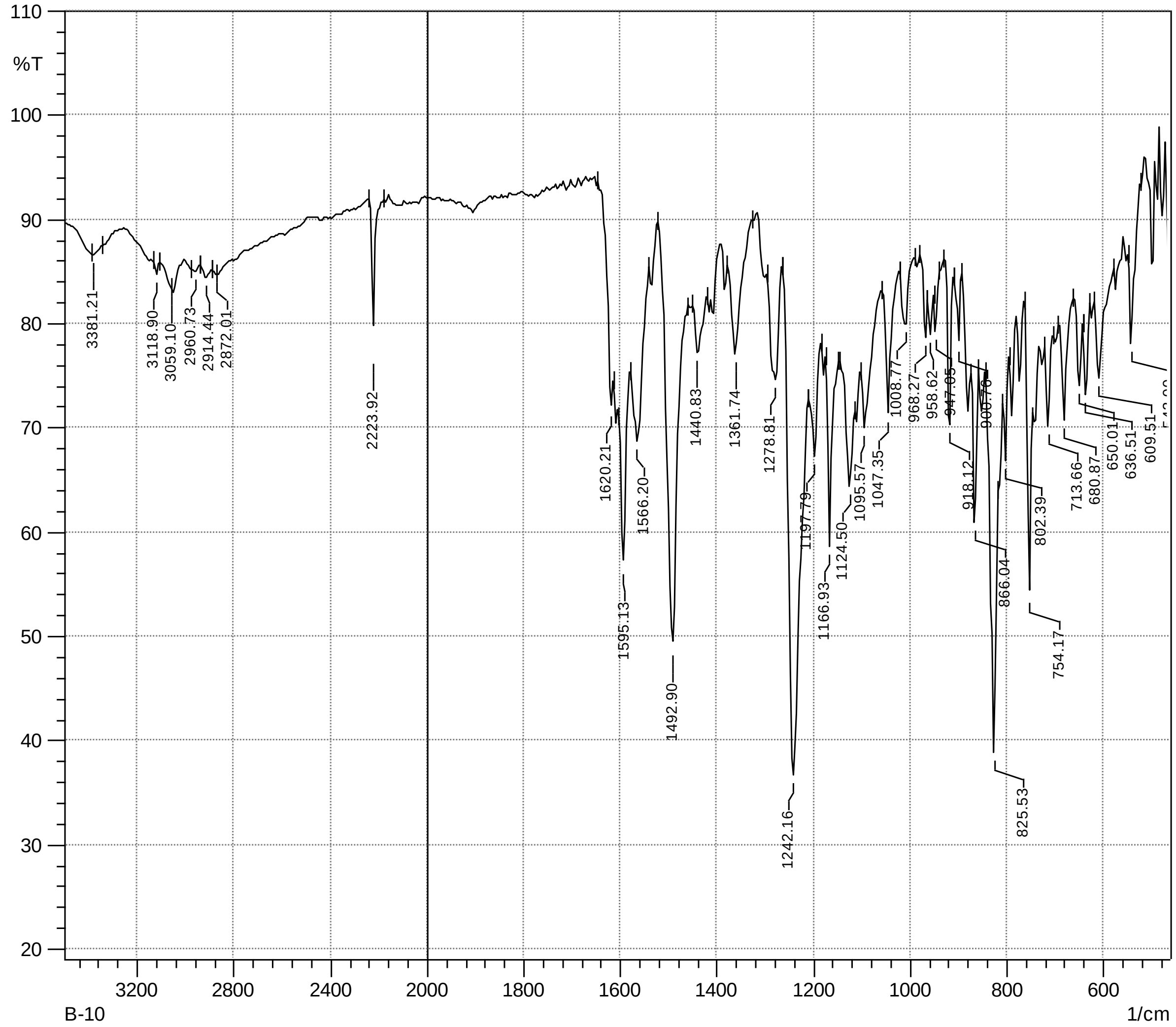

Figure S43. IR Spectrum of compound **10**

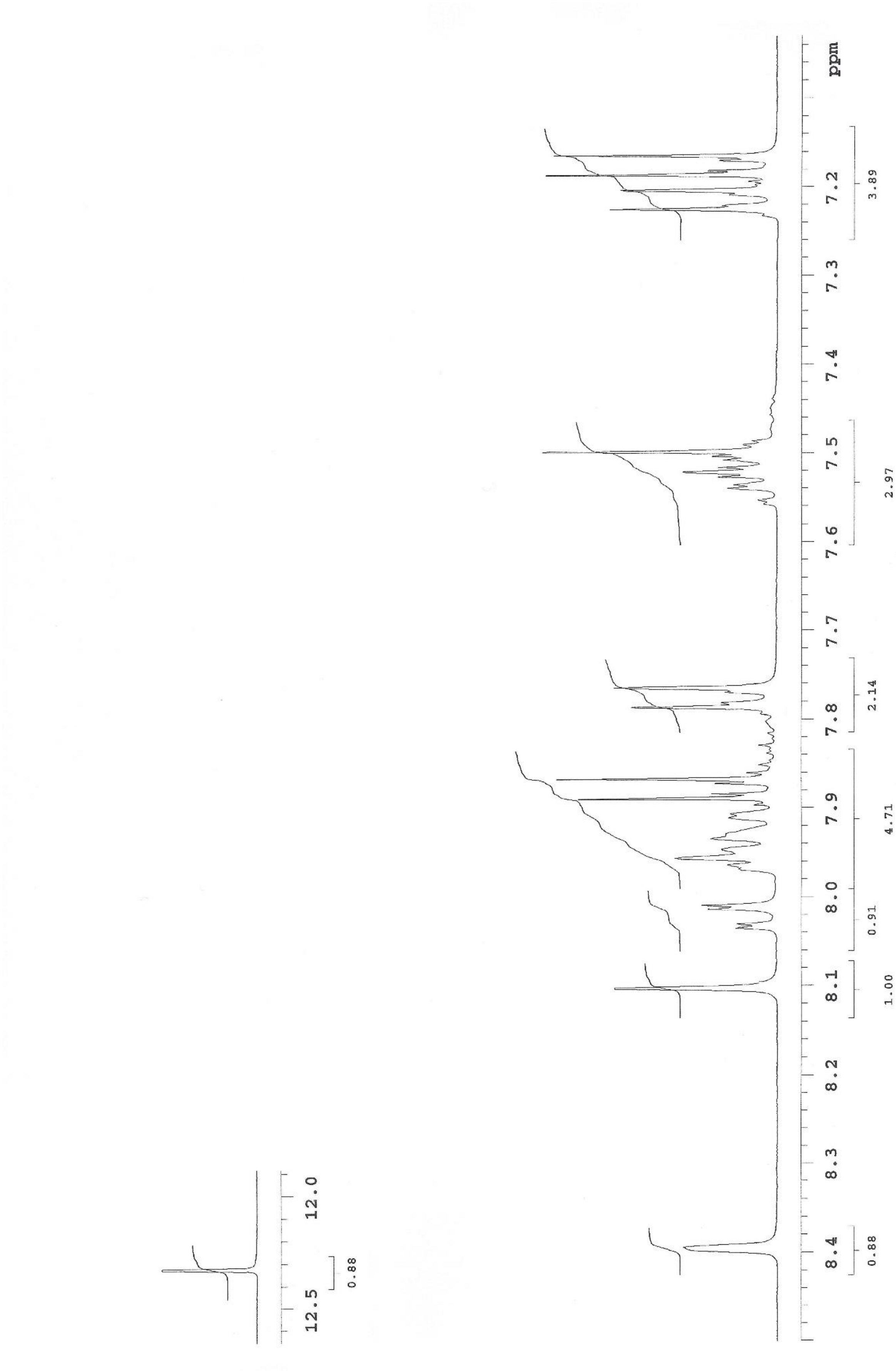

Figure S44. ^{1}H NMR Spectrum of compound **10**

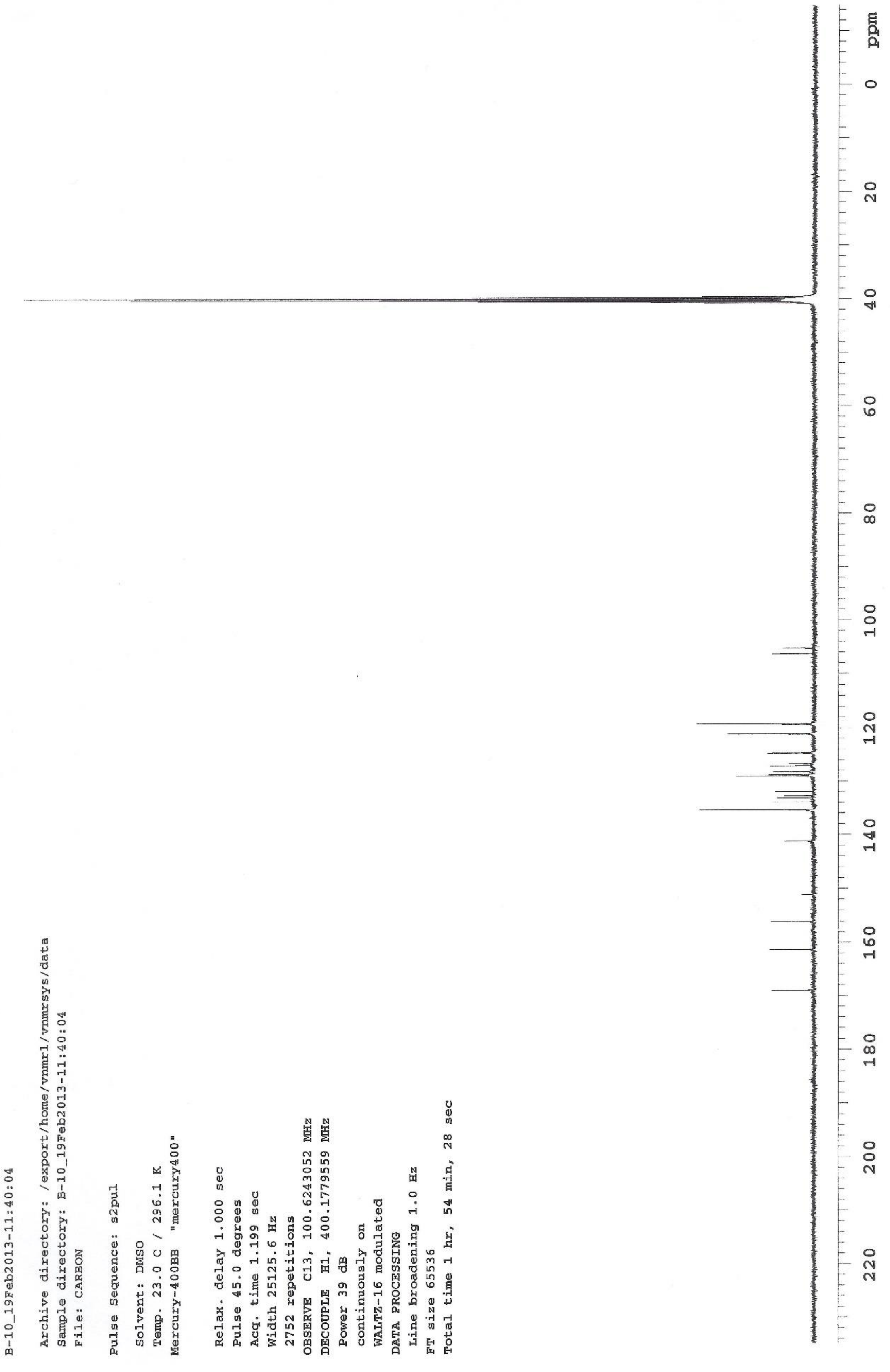

Figure S45. ^{13}C NMR Spectrum of compound **10**

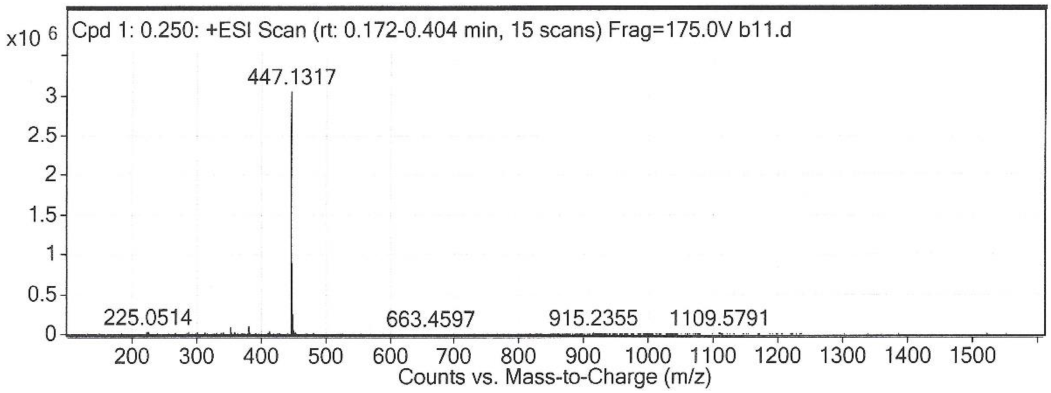

Figure S46. HRMS Spectrum of compound **10**
